# Climate stability and low population pressure predict peaceful interactions over 10,000 years of Central Andean history

**DOI:** 10.1126/sciadv.adt9007

**Published:** 2025-11-28

**Authors:** Weston C. McCool, Kurt M. Wilson, Elizabeth N. Arkush, Daniel A. Contreras, Brian F. Codding

**Affiliations:** ^1^ Department of Social Sciences, California Polytechnic State University, 1 Grand Avenue, San Luis Obispo, CA 93407, USA.; ^2^ Department of Anthropology, Lawrence University, 711 E. Boldt Way, Appleton, WI 54911, USA.; ^3^ Department of Anthropology, University of Pittsburgh, 3302 WWPH, Pittsburgh, PA 15260, USA.; ^4^ Department of Anthropology, University of Florida, 330 Newell Drive, Gainesville, FL 32603, USA.; ^5^ Department of Anthropology, University of Utah, 201 Presidents Circle, Salt Lake City, UT 84112, USA.

## Abstract

As anthropogenic climate change threatens to destabilize global societies and ecosystems, anticipating likely human responses becomes ever more urgent. A key global initiative is the promotion of peaceful relations. Nonetheless, studies that systematically evaluate factors that promote peace are limited, and research focuses on recent centuries when climate conditions were stable. Here, we couple evolutionary ecology theory with machine learning models to investigate the relative effects of climatological, demographic, and socio-political conditions on the persistence of peace over the 10,000-year Central Andean Holocene sequence. We find that stable climate conditions and low population density have a strong influence on peace, even when average climate conditions are not ideal for farming. Given that climate projection models predict increasing climate volatility in coming decades, our results suggest that future climate instability may weaken peaceful interactions, particularly among subsistence populations in marginal environments.

## Teaser

Ten thousand years of Central Andean history shows that peace endures when climate is stable and population density is low.

## INTRODUCTION

Researchers across the sciences have made concerted efforts to understand the conditions that promote human conflict (*1*–*3*), with particular focus on climate change as a stressor (*4*–*7*). While these studies offer insights into the conditions that heighten the risk of violence, it is equally essential to investigate the drivers of nonviolent interactions. Understanding the causes of positive-sum strategies becomes increasingly critical as modern societies strive to develop practices and institutions that foster amicable relations amidst the escalating impacts of global anthropogenic climate change (*8*). While some cross-regional research suggests a decline in violence over recent centuries (*9*, *10*), there is a paucity of studies that systematically quantify and evaluate factors that promote peace, and existing research focuses on the past few centuries when climatic conditions were relatively stable (*4*, *10*, *11*). Additional research is needed to anticipate likely responses to volatile climate conditions and identify socio-environmental conditions that foster nonviolent outcomes.

Archaeology is fundamental to this pursuit because it can couple general theory with quantitative models to evaluate the drivers of peace across diverse socio-environmental conditions and long timescales. Moreover, archaeological data can inform models of human-environment interactions during climatic intervals more volatile than those experienced in the past few centuries and therefore more comparable to future projected trends (*5*, *12*). To this end, the present study draws on evolutionary ecological theory to generate predictions regarding the conditions that promote peace and a recently published bioarchaeological database (*13*) that records skeletal evidence of violence for 7893 individuals throughout the Central Andes over a 10,000-year window. We investigate the relative effects of changing climatological, demographic, and socio-political conditions on the degree and duration of nonviolent interactions using Monte-Carlo sampling with a random forest (RF) regression machine learning modeling approach following Wilson *et al.* (*14*, *15*). We provide a theoretical framework and background summary followed by a high-resolution characterization of the long-term trends in peace and violence and assess how paleoclimate conditions, population dynamics, and socio-political complexity predict the persistence of peace.

**Fig. 1.**
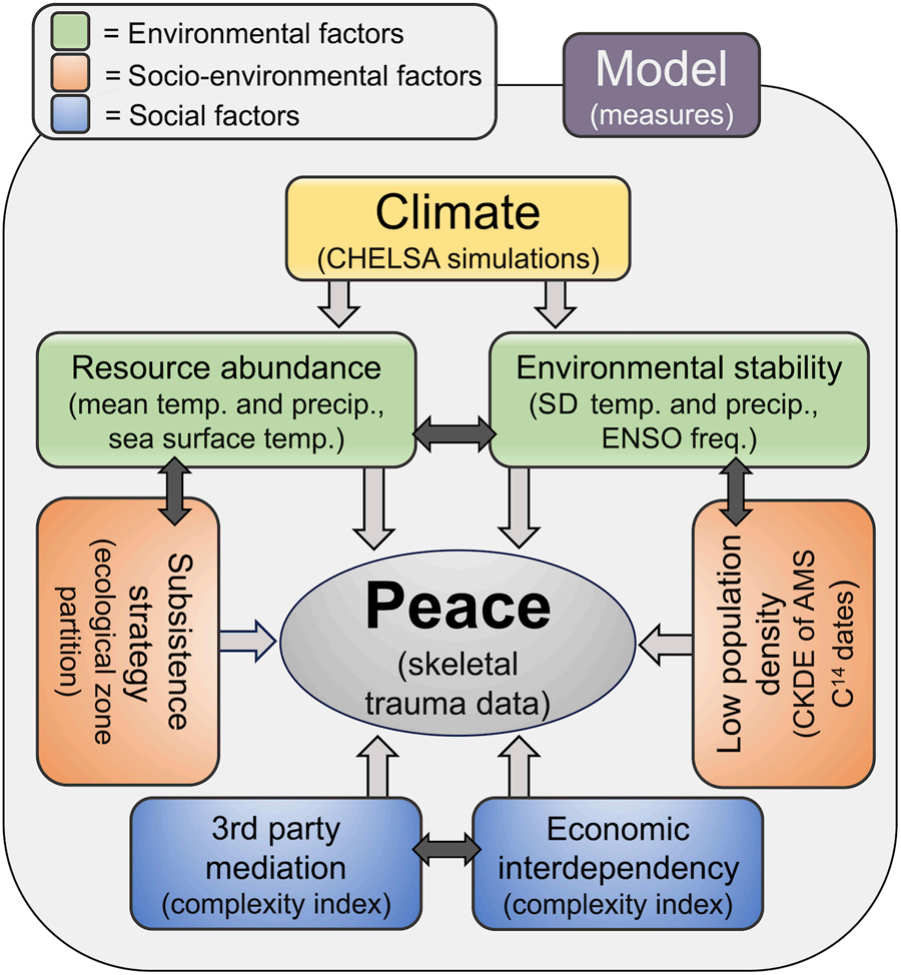
Systems diagram outlining our predictor and response variables and related proxy measures. Measures are generated separately for coastal and inland elevation zones to control for variation in baseline environmental conditions and macroscale economic strategies. Our model assumes linkages between all environmental and socio-environmental factors. While we assume relationships between social factors, it is beyond the scope of the current paper to hypothesize linkages between social and nonsocial factors. Predictor variables will be modeled to estimate their relative direct and interactive effects (see Materials and Methods). AWS, accelerator mass spectrometry.

Evolutionary ecology theory holds that individuals are neither inherently peaceful nor violent but have an evolved capacity for both and will use either strategy when the expected social and material benefits outweigh the costs (*3*). Thus, the frequency of peace and violence in a society depends on conditional payoffs within relevant socio-ecological constraints (*1*, *16*, *17*). Peaceful conditions ensue when the anticipated payoffs for positive-sum interactions outweigh the marginal benefits of violence. With respect to five key variables, we expect that peaceful interactions will prevail under five conditions (Fig. 1)

**Fig. 2.**
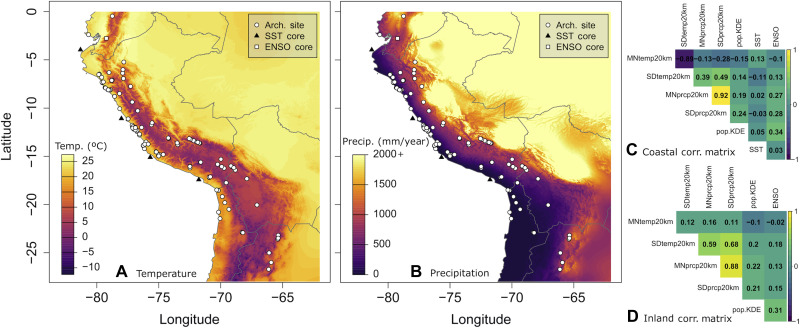
A map of the Central Andes (showing archaeological sites with skeletal samples, SST cores, ENSO core locations) and predictor covariance matrices. (**A**) As an example, spatially resolved (20 km) mean temperature for 1 kilo years before the present (k yr B.P.). (**B**) Spatially resolved (20 km) mean precipitation for 1 k yr B.P. (**C**) Covariance matrix for the coastal zone. (**D**) Covariance matrix for the inland zone.

1) Abundance: Resources are abundant and homogenously distributed, since these resources are seldom worth defending (*18*, *19*) or acquiring by force (*1*, *16*, *20*). We index resource abundance using spatial-temporal simulations of mean precipitation and temperature and estimates of sea surface temperature (SST; see Materials and Methods and Fig. 2). Peace should persist during cooler, wetter conditions on the coast and warmer, wetter conditions in the highlands, leading to resource abundance.

2) Stability: Environmental productivity remains consistent or follows predictable patterns (*21*), since individuals invest in subsistence strategies based on prior experience and may require time to shift investments toward alternatives when environments change. We index environmental stability using spatial-temporal simulations of SD in precipitation and temperature and estimates of El Niño–Southern Oscillation (ENSO) frequency (see Materials and Methods). Peace should persist during periods with less variable climate.

3) Demography: Population pressure is low, since high population pressure promotes competition by reducing per capita resource availability (*17*, *22*–*24*). We index relative population size using composite kernel density estimates (CKDE) of Central Andean radiocarbon dates. Peace should persist as long as CKDE values are low.

4) Complexity: Formal institutions centralize political decision-making within an elite subset of the population (state-level organization), suppressing intrasocietal conflict and retributive violence that may arise from environmentally or demographically driven factors (see above) (*20*, *25*–*27*). We index socio-political institutions using an ordinal measure of socio-political complexity, assuming that imperial political organization is more effective at suppressing violent conflict over a larger geography than smaller regional states.

5) Interactions: We predict that these factors will have important interactive effects that suggest relationships between proposed causal variables.

The Central Andes are characterized by remarkable diversity in geographic zones, ranging from hyperarid coastal regions to cold, high-elevation montane settings (*28*, *29*), and in cultures, from complex foragers to expansionist agrarian empires (*30*). During the Holocene, many populations transitioned from foraging to intensive agriculture and high elevation agropastoralism. After the onset of agriculture, the region experienced shifts between socio-politically integrated urban societies and “intermediate periods” when cross-regional systems broke down into localized configurations. During these transitions, demography, socio-political complexity, social practices, and subsistence economics varied markedly, often in parallel with fluctuating climatological and ecological conditions (*14*, *31*–*35*).

Andeanist scholars have used various kinds of evidence to document changing frequencies of violence throughout the Holocene (*12*, *31*). However, while numerous studies seek to understand local variation in peace and violence [e.g., (*17*, *36*–*38*)], cross-regional research remains limited as studies focus almost exclusively on single valleys or regions. Complementary studies have explored demographic dynamics (*14*, *34*, *39*) and proposed long-term trends in climate-driven socio-environmental change (*14*, *35*, *40*–*42*). These studies provide an outline of socio-political, climatological, and demographic conditions across the region over the past 10,000 years, making it possible to assess how these conditions relate to the frequency of both violent and nonviolent behaviors across vast spatio-temporal scales.

## RESULTS

Here, we report results for the coast and inland regions (see Materials and Methods) including the approximate probabilities of peaceful interactions (i.e., the absence of violent trauma) across the range of predictor values, which we term the PPI range (e.g., the PPI range for coastal terrestrial temperature is 0.67 for low temperature to 0.84 for high temperature). This is followed by a section on model performance.

### Coastal results

**Fig. 3.**
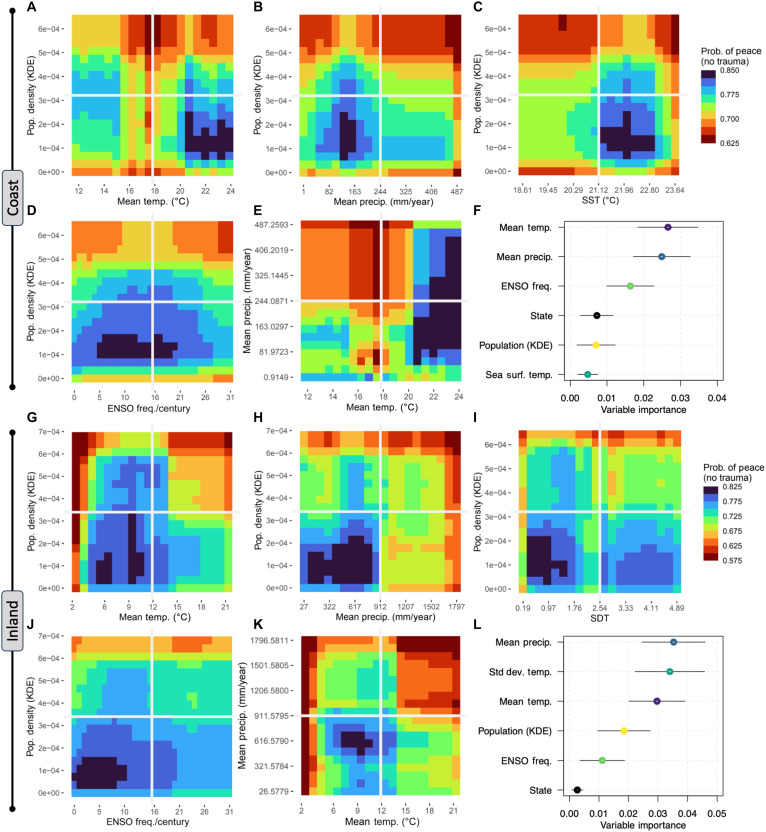
Summary of model results. (**A** to **E** and **G** to **K**) Predicted partial response of peace (blue) relative to violence (red) for important variable combinations. Results of the coastal model show that peaceful interactions are most likely when population density, mean temperature, and ENSO frequency are low and when land and SST are high. Results of the inland model show that peaceful interactions are most likely when population density, temperature, precipitation, and ENSO frequency are low. Partial dependence plots are shown in code S1 figures. The socio-political complexity (state) variable is omitted from plotted results because bivariate trends are flat and interactive effects are weak. (**F** and **L**) In the variable importance plots, colored dots provide the central tendency value for variable importance from the aggregate 1000 models, black lines are two SDs around the mean, and the white line provides the 95% CI on the mean. SDT, standard deviation temperature.

The probability of peaceful interactions (PPI) on the coast is highest when (i) terrestrial temperatures are warm (0.67 to 0.84 PPI range) and SSTs are warm (0.75 to 0.84 PPI range), (ii) mean annual precipitation is low to average (0.80 to 0.85 PPI range), (iii) ENSO events are of less to average frequency (0.80 to 0.85 PPI range), and (iv) population density is low (0.70 to 0.825 PPI range) (Fig. 3, A to F). State complexity has a weak effect (0.82 to 0.82 PPI range), suggesting that nonviolence is not strongly influenced by complexity. These results do not support resource abundance as a driver of peace, since nonviolence peaks when terrestrial and SSTs are warm and rainfall is minimal, which are the opposite of prediction one. However, these conditions do support prediction two, since peaceful interactions persist under stable climate conditions (low SDs), with warmer temperatures and reduced rainfall being linked to climate stability. Prediction three is also supported, with low population density correlating with peaceful interactions. Prediction four is not supported, as peace declines slightly as socio-political complexity increases (code S1, figures).

### Inland results

Peace-promoting climatological conditions away from the coast are characterized by (i) low precipitation (0.65 to 0.77 PPI range), (ii) low SD in temperatures (0.72 to 0.77 PPI range), (iii) a mean temperature sweet spot, where average temperatures are neither extremely hot nor cold (0.67 to 0.82 PPI range), and (iv) infrequent ENSO events (0.75 to 0.78 PPI range) (Fig. 3, G to L). The model suggests that initial population growth fostered peace but that violence increased with high population densities (0.67 to 0.80 PPI range) (code S1, figures). In addition, there is a small state-level effect (0.71 to 0.72 PPI range) showing that nonviolence declines slightly with increasing state control (code S1, figures). These results do not support prediction one, as reduced precipitation and cool temperatures likely negatively affected average environmental productivity. However, they do support prediction two: Climate stability increases where precipitation is low, temperature is less variable, and mean temperature is in a sweet spot, avoiding climate shocks from extreme cold or hot temperatures. Prediction three is also supported, with low population density correlating with more nonviolent interactions. Prediction four is not supported, as peace declines with increasing socio-political complexity.

### Model performance

Using 7983 individuals with the recorded presence/absence of violent trauma and the estimated paleoclimate, relative population density, and sociopolitical complexity conditions they experienced, RF logistic regression trains and tests models that predict the probability that individuals did not experience violent interactions during their lifetime (code S1, fig. S3). Iterated Monte Carlo analysis of 1000 coastal RF models produce a mean area under the curve (AUC) of 0.67 [95% confidence interval (CI), 0.59 to 0.74] with a mean square error (MSE) of 0.14 (95% CI, 0.13 to 0.15), while 1000 inland RF models produce a mean AUC of 0.69 (95% CI, 0.61 to 0.77) with a MSE of 0.16 (95% CI, 0.15 to 0.18), suggesting that models discriminate when individuals are likely or unlikely to experience violent trauma (code S1, fig. S21). Accuracy measured from complete data models shows a log-loss of 0.27 (95% CI, 0.25 to 0.28) and prediction probabilities of 0.77 (95% CI, 0.76 to 0.78) for both coastal and noncoastal model sets and suggests that the model reasonably predicts trauma/no trauma (code S1, fig. S22). Across both the coastal and inland model sets, climate variables have greater influence than socio-political and demographic variables (Fig. 3, F and L). Predicted probability of no trauma results visualized in three-dimensional (3D) partial response plots capture interactions between key predictor variables (Fig. 3), which are more informative than partial response plots showing bivariate relationships (code S1, figs. S24 to S30 and S37 to S43).

Autocorrelation tests between predictor variables demonstrate that mean precipitation is strongly correlated with precipitation variability (Fig. 2, B and D, and code S1, fig. S20), suggesting that increasing overall precipitation also indicates greater spatial precipitation instability. Mean temperature on the coasts is inversely correlated with temperature variability such that warmer temperatures are associated with greater uniformity or stability (Fig. 2B).

## DISCUSSION

Results suggest four key inferences: (i) Climate stability is more important than climate averages for maintaining peace, (ii) initial population increase can promote peace until a tipping point when additional growth leads to violence, (iii) the effect of socio-political complexity on frequency of violence is dampened by climate and demography over large spatiotemporal scales, and (iv) interactions between climatic and demographic variables best predict peace. These trends are evident in both the coastal and inland models (Fig. 3), which we discuss separately to account for the effects of different socio-environmental conditions.

### The coast

Coastal results support predictions two (stability), three (population density), and five (interactive effects) and do not support predictions one (abundance) and four (socio-political complexity). The lack of support for prediction one can be explained by examining the factors that influence mean environmental productivity on the Andean coast. Cold SSTs in the Humboldt Current off the coast of Peru increase nutrient loads, boosting overall marine productivity (*43*–*45*). In addition, increased precipitation enhances plant growth and quality, raising agricultural yields (*46*–*48*). Mildly warm temperatures increase air moisture, whereas excessively hot temperatures increase evaporation, reducing the already scarce moisture in arid coastal environments (*49*). Nonetheless, peaceful interactions peak where and when SSTs are warm, precipitation low, and temperatures high.

While initially counterintuitive, these circumstances support predictions two (stability) and five (interaction effects). Climate conditions that enhance average marine and terrestrial productivity also increase climate volatility, making conditions less predictable and raising economic risk (*21*). Consequently, the most violent conditions arise where high population densities in circumscribed coastal valleys interact with unpredictable climate, even when average conditions are favorable. Prior research shows that warmer SSTs are associated with a greater reliance on terrestrial agriculture, particularly maize (*15*). This suggests that terrestrial resources may have played an equal role in structuring average resource availability and predictability relative to marine resources, especially in more recent periods.

Also in support of predictions two and five, results show that infrequent ENSO events are associated with peaceful interactions. This may be due to ENSO events creating uncertainty in local climate as the Walker Circulation reverses, leading to a rapid influx of precipitation on the otherwise hyperarid coast. While this increase in moisture can sometimes result in coastal conditions favorable for plant growth (*48*), ENSO events are also linked to destructive events such as flooding, erosion, and catastrophic landslides (*35*). Several researchers have noted an antiphase phenomenon between coastal and highland climate regimes (*50*), suggesting that coastal aridity may coincide with increased runoff from highland precipitation into coastal drainages. While existing data do not allow for a test of this hypothesis, it could suggest support for prediction one if dry coastal conditions correlate with wet highlands conditions and attendant increases in surface water flow in coastal valleys. We hope to test this hypothesis with future research.

Last, results support prediction three, as the shift from nonviolent to violent interactions occurs as population density peaks in the coastal region. This may be due to the extreme circumscription of coastal environments, with initial positive effects resulting from economies of scale (*51*) that in turn promote further demographic growth, followed by a tipping point when resources became pressured, and violent competition escalated. To summarize, what appears clear is that stable climate conditions combined with low population density best predict coastal peace.

### Inland

Highland results show that cool, dry, and stable climatological conditions coupled with low population density best predict nonviolent interactions, supporting predictions two (stability), three (population density), and five (interactions) with no support for predictions one (abundance) and four (complexity). These results align with prior work showing that societal instability in the Andean highlands is partly driven by climate volatility (*21*). The negative relationship between the frequency of nonviolence and mean precipitation contrasts with several regional studies that link dry conditions to social instability and violence [e.g., (*36*–*38*, *52*)]. Mean precipitation positively covaries with the SD in precipitation and temperature, showing that wet upland climate conditions correlate with unpredictability. This likely led to nonlinear trends where favorable averages were accompanied by unanticipated extremes, causing harmful mismatches between economic strategies and experienced environmental conditions.

In addition, mean temperature and precipitation strongly interact (Fig. 3K), suggesting that peace is most enduring when precipitation and temperature levels are adequate for crop growth but low enough to avoid elevated volatility. Climate instability predicts a reduction in peaceful interactions, especially when volatility coincides with high population densities, indicating that these populations periodically experienced scalar stress and attendant resource strain (*17*). Given that inland environments are typically characterized by marginal, stochastic, and often circumscribed baseline conditions (*53*–*55*), climatic changes that mitigate these challenging circumstances may have fostered persistent nonviolent interactions. This suggests that while drought conditions affected peace and violence at some sites, drought itself is not a universal cause but has significant impacts when also associated with greater climatic instability (i.e., antiphase events) (*21*).

### Local constraints on climate resilience

Typically, human adaptations to climate downturns or shocks involve migrating to less affected areas (*56*–*59*) or intensifying food production (*56*, *60*, *61*). In the marginal and circumscribed Central Andean environments, groups invested heavily in economic infrastructure and landesque capital (*28*), incentivizing territoriality and making relocating to more productive or less dangerous areas exceedingly costly. This promotes economic intensification as the primary adaptive strategy, which provides short-term relief from population pressure but can reduce long-term resilience as populations continue to rise, economic efficiency decreases, and subsistence strategies become riskier. This “intensification trap” heightens a population’s vulnerability to climate instability, scalar stress, and resultant violence.

### The Leviathan

The weak effect of the complex state variable is intriguing and suggests several interpretations: (i) Compared to climatological and demographic factors, state or imperial political organization has a weak relative effect on violence and peace and does not have strong interaction effects with climate or population (code S1, figs. S49 to S58). (ii) This may indicate a need for more fine-grained data to capture variation between polities in socio-political centralization and institutional practices affecting violence and/or reflect interregional differences in how state-level societies are defined by researchers. We therefore contend that this result be considered tentative and recommend future research to further quantify and standardize cross-regional measures of socio-political factors to better investigate these processes.

### Predicting peace

Model results support the theory that peace and violence are strategies sensitive to conditional payoffs in local socio-ecological contexts. Regional trajectories fluctuate between violent and nonviolent interactions depending on local conditions, which are strongly keyed on climate change. We find that climate stability was more important than climate averages for predicting peace, which we argue is the result of precarious baseline environmental conditions making populations vulnerable to resource shortages owing to climate volatility and scalar stress. Future research will benefit from refined measures of spatio-temporal variation in SSTs and ENSO frequencies, demographic transitions on cross-regional scales, the quantification of socio-political processes at higher cross-regional resolutions, and additional skeletal trauma data from a variety of contexts.

### Future climate change

Climate projection models converge on the prediction that future climate regimes will be more volatile than those in recent centuries (*62*). Given the United Nations Sustainable Development Goal of promoting peaceful societies and institutions, it is evident that amicable relations must be built within a context of increasing climate instability, which our research shows consistently deteriorate peaceful interactions. This suggests that dense populations in marginal and stochastic environments will be particularly vulnerable to climate-driven violence. This is particularly true where risk mitigation strategies such as exchange, migration, and economic diversification are constrained because of sectarian or national borders, homogenizing market forces, or intensification traps. While additional research is needed, the general theory and empirical modeling framework developed here can be applied to other case studies to better understand the relationship between peace and climatic change, aiming to predict likely human responses to anthropogenic climate change in the near and distant future.

We must also acknowledge that our dataset does not capture the complete relational nuances of peace, as it cannot distinguish between forced nonviolence and true positive–sum interactions (*26*). Nevertheless, our model identifies major inflection points between violence and nonviolence, suggesting that local environmental and demographic conditions and exogenous climate factors are key predictors.

## MATERIALS AND METHODS

### Violent trauma database

Violence and nonviolence can accurately be assessed through population-level rates of cranial and facial trauma, the most common kind of trauma associated with interpersonal violence (*63*–*65*) and one that is frequently relied on by bioarchaeologists (*66*, *67*). We draw on a dataset compiled by Arkush (*13*) from published articles, reports, and dissertations reporting trauma on adults from archaeological contexts throughout the Central Andes. An early version of the dataset was published by Arkush and Tung (*31*), and the dataset was later expanded to include more recent published sources up to 2021. The dataset aggregates 92 different studies covering 7983 adults from 116 sites or site clusters, some multicomponent, spanning the Holocene (data S1). Multicomponent sites are subdivided by period, following source reports, with one record (row) per individual in a site and time period. The records give the number of adult individuals who present craniofacial trauma and those that do not. The dataset follows source reports in categorizing individuals as adults and subadults. Subadults present craniofacial injury much more rarely than adults in Andean samples, so they are excluded from our analysis to prevent variable proportions of subadults in excavated contexts from biasing overall rates. Almost all injuries counted in this dataset consist of blunt-force trauma. The dataset does not count trauma that was evidently not interpersonal violence with intent to harm (e.g., trepanation and perimortem defleshing). Unusual contexts such as massacre deposits or sacrifice victims were not included in the dataset. In all, the dataset contains 6284 individuals without trauma and 1699 with trauma (21.3%). For the coast region, there are 2889 individuals without trauma and 615 with trauma (17.6%), while for the inland region, there are 3395 individual without trauma and 1084 with trauma (24.2%).

### Climate data

Climate reconstructions of spatiotemporally varying local terrestrial precipitation (mm/year), temperature (mean annual °C), and variability in each are obtained using the CHELSA-TraCE21k downscaled past climate estimates, which are based on simulation and proxy matching (*68*, *69*). From the CHELSA dataset, we obtain climate estimates at ~1-km grid cell resolution for each century from 11950 to 0 years before the present. To account for the absence of radiocarbon dating of individuals in the dataset, climate variables are associated with each individual using a Monte Carlo sampling approach [following (*14*, *15*)] whereby we extract the mean precipitation, mean temperature, and SDs in precipitation and temperature for a 20-km radius from an individual’s spatial location for each century in which they may have been alive. This set of climate data is then sampled, with replacement, 1000 times, generating a probability distribution of the terrestrial climatic conditions most likely experienced, which is stored for use in the statistical analyses. This method estimates spatial variability in climate and checks for all the temporal combinations of spatial variability within an individual’s likely time window. We use a 20-km radius to capture the most likely locally accessed areas for each individual, which we validate for consistency by testing the correlation between terrestrial climate variables at 5-, 20-, and 50-km radii, showing that the climate conditions at each are tightly correlated (code S1, figs. S17 to S20) such that use of 20-km data reliably captures local climate.

Following Wilson *et al.* (*15*), SST and ENSO frequency data are acquired using published core data. Spatially varying SST is derived from alkenone-derived SST estimates from four ocean cores (M77/2_757, M77/2_024–5, M77/2_003–2, and M135_254–3) located at ~4°, 11°, 15°, and 17° south (*70*, *71*). Alkenone SST estimates in the region broadly reflect the warmer summer ocean temperatures and are not correlated with ENSO (*70*, *71*). SST estimates are associated with each individual spatially, using the nearest core to establish their experienced SST. ENSO frequency per century is estimated using the Laguna Pallcacocha sediment core (*72*). Given the lack of spatially varying quantifications of ENSO frequencies in the region, we use this single core for each individual. We implement the same Monte Carlo sampling procedure as above for SST and ENSO estimation, subsetting the cores to the temporal window of each individual and resampling 1000 times. For the analyses, SST is only included for coastal individuals. While SST is teleconnected to inland climate, that connection varies spatially in ways that are inconsistent across the latitudinal and longitudinal gradients of the inland portions of the study region, preventing clear alignment with predictions.

### Population estimates

To estimate relative past population sizes, we use a CKDE version of the “dates as data” approach (*73*–*76*), following Wilson *et al.* (*14*). For a full discussion of the method, its biases, implementation, controls, and appropriateness in this study, see code S1. The dates as data approach relies on the assumption that larger populations produce more dateable material, which will be dated, leading to larger counts of dates and greater values in summaries of date distributions (*34*, *77*–*81*). This approach has been critiqued almost as widely as it has been used, but it remains the only population proxy that is relatively readily available at multiple temporal and spatial scales, making it indispensable for regional and long-term analyses [see (*77*, *82*, *83*) and code S1 for overviews] with recent work providing key interpretive and methodological best practice advances [see (*75*, *77*, *82*–*84*)].

In keeping with accepted best practices for quality control, we drop dates with large errors (>100 years) due to their disproportionate impact on date summaries (*34*, *85*). Further, on the basis of the results of a sensitivity test (code S1) and following prior Andean dates as data research (*78*, *86*), we use hierarchical clustering, or binning, of dates using a 200-year cutoff to control for overrepresentation of single sites (*34*, *75*, *81*); although this may underrepresent large/urban sites, the aggregate patterns remain robust based on a binsense analysis (code S1). To avoid over or under subregional sampling and to align radiocarbon data with individuals, we assign each date to a coastal or inland category using the same criterion for each individual (see below), creating separate, aggregate, population density estimates for both regions. All terrestrial dates are calibrated using SHCal20, while shell-derived dates are calibrated using Marine20 with reservoir effects obtained from calib.org (*87*). Landscape taphonomy can also affect dates as data approaches, with debate continuing regarding applications of taphonomic loss corrections [e.g., (*88*–*90*)]. While a global correction may not be appropriate because of unintended effects [see code S1 (774 to 916) and figs. S8 to S12 for discussion and details], we validate model outputs by running a representative sample of coastal and inland models using taphonomically corrected (*89*) KDEs, showing that the analytical and interpretive outputs of models using nontaphonomically corrected and taphonomically corrected KDEs are functionally identical (code S1, figs. S59 to S76).

To acquire estimates of relative population size for each individual, we implement a Monte Carlo sampling procedure (code S1). We first create 1000 unique KDE estimates for each region using the rcarbon (*75*) package in the R statistical environment. Then, for each individual, we repeat the following process 1000 times: (i) randomly select 1 of the 1000 KDEs for their region, (ii) subset the KDE to the individual’s time range, and (iii) randomly select one year from the time range and extract and store the KDE value of that year. This results in 1000 unique population size estimates per individual, generating a probability distribution of experienced relative population pressure.

### Socio-political complexity

We classify socio-political complexity with a simple ordinal measure of 0 = nonstate, 1 = state, 2 = empire. Measure two is used exclusively for the Inca empire, which achieved much larger consolidated geographic control than Andean state precursors such as Wari or Chimú, and for which there is clearer evidence of effective internal suppression of warfare, based on regional shifts to less defensive settlement patterns (*31*). This coding scheme follows Arkush (*13*) with the exception of the distinct category for the Inca empire. Communities that are peripheral to and only possibly influenced by states are coded as nonstate (0) and include autonomous groups and regional systems without a clear state-level core. States are defined as regional polities with a clear political center or capital, as evidenced by settlement patterns and major disparities in wealth, status markers, and monumentality, with the caveat that, for several cases, the “state/nonstate” categorization is debatable on current evidence and should be considered provisional. Sites are categorized as a state (1) if they (i) fall within a state’s core heartland or are clearly state-associated colonies or (ii) are political entities spanning a large area with a centralized urban core. Sites are categorized as Inka empire (2) using evidence for Inka administrative control. Although this approach limits the complexity measurement to three levels, there is ongoing debate regarding the sociopolitical complexity of various societies in the Central Andes. Adding additional levels would increase precision but at the cost of accuracy. We argue that the current approach strikes a balance by maximizing the probability that each society is categorized correctly, even if the coarse nature of the measurement overlooks some important variation.

### Statistical models

We use statistical models to estimate the probability that an individual did not experience violent trauma during their lifetime by testing climate, demographic, and socio-political variables that might structure that probability. Models are partitioned into two geographic categories—coastal and inland—adapted from Wilson *et al.* (*14*) and Pulgar Vidal (*29*). The zone partition (coast/inland) was designed to (i) differentiate demographic estimates between coastal and inland zones and (ii) provide flexibility to include or exclude SST and ENSO frequency variables from inland sites, which have a more complicated and now less understood relationship with oceanic conditions. The coastal zone contains sites of <15 km from the coast and under 500 meters above sea level (masl). The mid-elevation/highland zone contains sites of >15 km from the coast or above 500 masl. Since our terrestrial climate, oceanic climate, and socio-political variables are estimated on the basis of proximity to skeletal samples (using Euclidean distance or a 20-km buffer), these measures are designed to account for continuous variation across environmental gradients.

To assess our predictions, we use RF regression (*91*–*93*), a machine learning ensemble decision tree method implemented via the ranger package (*94*) in the R statistical environment (*95*). We use RF because it can handle and identify interactions between variables, work with variables with autocorrelation, and evaluate nonlinear relationships through subsampling predictor variables to prevent overreliance on a single predictor, helping to limit overfitting (*96*). Regression estimates probability forests, which are more accurate (*97*) and enable us to more directly assess model discrimination (*98*) while continuing to address potential overfitting (*96*). Although RFs can handle correlated predictor variables, we test for autocorrelation before model analysis to identify any variables correlated at a strength of 0.7 or higher, as potentially problematic (*99*). Autocorrelation tests are performed for coastal and inland samples separately. In both regions, mean precipitation is strongly positively correlated with the SD of precipitation, while on the coasts, mean temperature is also strongly inversely correlated with SD in temperature (Fig. 2, B and D). Therefore, we leave SD in precipitation out of both final model sets, and we remove SD in temperature from the coastal models, favoring mean precipitation and temperature as they capture both environmental productivity and stability/instability.

Two sets of RF models are created for the analysis, one set for coastal and one for inland individuals. To control for the temporal uncertainty of individuals in our dataset, we propagate temporal uncertainty into the RF modeling approach through running 2000 unique models, 1000 each for coastal and inland data subsets using each of the 1000 resampled data points per individual. In effect, this treats every observation from the Monte Carlo sampling procedure as a “true” model, enabling us to evaluate the aggregate output of the 1000 models for underlying patterns that span the per individual possible climate and population combinations. The individual trauma record data are imbalanced (~75 to 80% individuals have no trauma), but this is not prohibitive for logistic regression. An additional potential concern is that individuals from the same site share similar predictor variable values, and some sites have >100 individuals, while others only have a few. While the larger sites should carry some additional weight in the model, reflecting larger populations, excessive imbalance could potentially overfit results to large sites. To control for this, in each of the 1000 model runs, we randomly select a maximum of 30 individuals per site to be incorporated into the model. This ensures that, in each model, all sites are represented, that greater weight is provided to individuals from sites with larger sample sizes reflecting larger populations, but that we limit the effect of large sites to ensure that model signals reflect patterns from all individuals. Comparison of models using all individuals and those subsampled as described here shows no meaningful difference in outcomes.

To directly assess our predictions, we must generate partial responses (i.e., how a change in a predictor variable affects the likelihood of trauma or no trauma) for all RF models. Partial response output is an estimation of the probability an individual with a given value of an independent variable will have or not have trauma. Model performance is assessed using MSE and AUC based on splitting the data into stratified training and testing subsets, preserving the ratio of yes and no trauma across the split. To evaluate overall model effectiveness, a second version of each of the 1000 models is run incorporating all data from which we calculate log-loss and log-loss as probability for model accuracy. For each of the 4000 models, we save the MSE, AUC, log-loss, log-loss as probability (measure of accuracy), permutation variable importance (increase in error rate/inaccuracy for dropping the variable), and partial response of the probability of an individual having no trauma to a change in each predictor variable, while the others variables’ effects are held constant. We also generate 3D partial response plots for coastal and inland areas to show the interactive effects of climate variables and population on the probability of no trauma with a second set of 3D response plots evaluating the interactions of climate and sociopolitical complexity (see code S1). Because of time and computational intensity, we are unable to run the 3D responses for every model. Partial responses are generated using the pdp package in R (*100*). The output of this procedure is, for each region, 1000 unique estimates of the ability of the climate, population, and sociopolitical complexity variables to explain whether an individual experienced trauma or not, with temporal uncertainty included. Assessment of predictions relies on the aggregated model performance metrics and partial responses. Visualizations of all 2000 model single variable 2D partial responses are provided in code S1. 3D partial responses visualized in our results are the aggregate results of the 200 models for which 3D partial response were generated.

## Supplementary Materials

### This PDF file includes:

Data S1 Code S1
