# Supplementary material for: Climate stability and low population pressure predict peaceful interactions over 10,000 years of Central Andean history
Source: Sci Adv. 2025 Nov 28;11(48):eadt9007. doi: 10.1126/sciadv.adt9007 (PMC12662215; doi:10.1126/sciadv.adt9007)
Supplement: Supplementary file 1 — Data S1 Code S1 [file sciadv.adt9007_sm.pdf]

Science Advances
AAAS

Supplementary Materials for

Climate stability and low population pressure predict peaceful interactions over 10,000 years of Central Andean history

Weston C. McCool *et al.*

Corresponding author: Weston C. McCool, wmccool@calpoly.edu

Sci. Adv. **11**, eadt9007 (2025)
DOI: 10.1126/sciadv.adt9007

This PDF file includes:

Data S1
Code S1

data S1: Full dataset. For references see published dataset in Arkush (2022) (*13*).

ID	Long	Lat	Start_dat	End_dat	dat_type	trauma	N
127	-69.173	-15.997				4	19
189	-70.19	-18.534	-800	600	uniform	23	163
139	-69.668	-19.804	-1000	600	uniform	19	45
196	-70.173	-20.791	-500	600	uniform	1	9
4	-76.26	-13.893	-8340	-6220	uniform	0	2
112	-69.726	-16.233	-6000	-4500	uniform	1	13
174	-76.702	-10.278	-7000	-3500	uniform	0	1
6	-76.754	-12.433	-5400	-5000	uniform	0	69
5	-69.557	-17.735	-5000	-4540	uniform	0	1
87	-71.762	-13.591	-4400	-3100	uniform	0	3
171	-79.336	-7.88	-5000	-2000	uniform	1	7
14	-75.217	-14.516	-3800	-3000	uniform	1	2
9	-70.323	-18.485	-4500	-1800	uniform	23	96
10	-70.056	-21.453	-3800	-2000	uniform	1	27
60	-80.67	-2.388	-3400	-1500	uniform	4	27
13	-70.322	-18.485	-3200	-1250	uniform	19	85
11	-69.872	-19.177	-2400	-1900	uniform	0	13
188	-69.102	-16.075	-3000	-1200	uniform	1	12
12	-78.17	-8.491	-2500	-1700	uniform	0	12
173	-79.108	-8.099	-1500	-1200	uniform	2	16
93	-78.754	-7.096	-1500	-1200	uniform	0	2
115	-77.07	-11.902	-1500	-1200	uniform	1	16
175	-76.702	-10.278	-1800	-800	uniform	0	3
172	-79.013	-6.307	-1200	-800	uniform	1	3
38	-79.563	-7.363	-1200	-800	uniform	0	9
95	-78.799	-7.164	-1200	-800	uniform	0	1
62	-76.82	-12.278	-1100	-800	uniform	1	18
116	-76.556	-12.798	-1500	-400	uniform	0	19
162	-79.299	-7.91	-1200	-500	uniform	1	6
97	-78.799	-7.128	-800	-500	uniform	1	2
131	-79.013	-6.307	-800	-500	uniform	3	63
39	-79.563	-7.363	-800	-500	uniform	0	8
56	-68.816	-16.43	-1500	100	uniform	0	7
130	-68.944	-16.179	-800	200	uniform	5	104
94	-69.173	-15.997	-800	200	uniform	4	18
177	-76.249	-13.865	-430	-200	uniform	49	124
98	-78.763	-7.128	-500	0	uniform	0	3
180	-76.032	-13.507	-300	-150	uniform	1	4
192	-72.174	-13.384	-400	100	uniform	5	7
64	-75.206	-14.547	-400	100	uniform	5	20
77	-78.892	-8.1	-400	100	uniform	5	15
40	-79.563	-7.363	-400	100	uniform	1	18
150	-76.159	-14.206	-350	50	uniform	3	3
178	-76.249	-13.865	-350	50	uniform	10	47
176	-76.127	-13.744	-300	100	uniform	2	17

114	-69.981	-15.301	-200	50	uniform	0	1
41	-76.909	-12.25	-100	100	uniform	9	61
117	-76.914	-12.212	-200	200	uniform	8	17
71	-78.891	-8.1	0	300	uniform	14	73
145	-76.914	-12.212	1	300	uniform	2	21
21	-68.21	-22.92	-200	600	uniform	5	99
27	-75.005	-14.968	1	450	uniform	2	13
151	-72.248	-13.349	-200	700	uniform		
140	-74.94	-14.819	1	650	uniform	0	1
17	-74.207	-13.157	100	600	uniform	1	11
76	-75.036	-14.843	1	700	uniform	1	19
88	-79.299	-7.91	250	600	uniform	11	30
89	-78.567	-8.788	300	450	uniform	3	6
83	-79.608	-6.804	200	600	uniform	0	9
15	-75.22	-14.544	100	750	uniform	1	19
186	-72.48	-16.206	200	700	uniform	67	100
118	-77.123	-11.941	300	600	uniform	6	14
61	-78.75	-0.461	300	600	uniform	1	6
28	-75.003	-14.97	450	550	uniform	1	38
126	-65.923	-26.005	400	600	uniform	19	50
184	-78.99	-8.134	300	850	uniform	5	38
185	-78.992	-8.133	300	850	uniform	0	23
132	-65.393	-23.593	575	680	uniform	0	13
29	-75.003	-14.97	550	750	uniform	4	30
59	-79.585	-7.32	500	900	uniform	4	40
22	-68.21	-22.92	400	1000	uniform	59	369
169	-69.557	-17.735	600	1000	uniform	87	206
158	-68.272	-22.889	500	1000	uniform	4	47
45	-67.073	-20.047	600	1000	uniform	1	3
57	-68.816	-16.43	600	1000	uniform	0	5
31	-70.923	-17.2	600	900	uniform	4	180
165	-70.926	-17.167	750	1000	uniform	2	15
36	-71.673	-13.634	600	1000	uniform	3	36
42	-71.716	-13.617	650	1000	uniform	1	3
30	-75.003	-14.967	750	900	uniform	9	98
19	-72.479	-16.013	650	800	uniform	13	39
16	-75.207	-14.547	750	1000	uniform	0	7
167	-72.488	-16.053	700	850	uniform	25	74
168	-72.488	-16.053	900	1050	uniform	7	30
80	-74.232	-13.098	600	1000	uniform	23	79
143	-76.909	-11.985	600	1000	uniform	1	6
99	-76.55	-12.696	600	1000	uniform	0	8
152	-75.855	-13.735	600	1000	uniform	5	23
153	-78.148	-10.061	800	1000	uniform	1	54
141	-74.94	-14.819	653	1025	uniform	0	4
119	-77.099	-12.07	600	800	uniform	12	52
144	-77.153	-11.77	500	1000	uniform	4	115

120	-77.088	-11.931	600	800	uniform	4	12
157	-76.91	-12.037	600	1000	uniform	4	16
74	-73.473	-13.792	850	1000	uniform	2	26
23	-68.21	-22.92	850	1200	uniform	16	44
54	-79.799	-6.468	900	1100	uniform	1	32
55	-79.305	-7.91	900	1100	uniform	2	27
113	-69.336	-20.492	900	1280	uniform	6	71
163	-70.837	-17.126	950	1250	uniform	1	6
164	-70.837	-17.126	1250	1476	uniform	0	19
24	-69.557	-17.735	950	1400	uniform	46	148
34	-77.822	-5.223	1200	1500	uniform	13	27
37	-71.929	-13.533	1000	1400	uniform	47	199
43	-77.915	-6.375	1100	1470	uniform	69	330
63	-69.921	-16.064	1100	1450	uniform	7	48
69	-77.472	-11.116	1000	1400	uniform	17	218
70	-68.944	-16.179	1000	1400	uniform	4	9
75	-73.515	-13.61	1150	1250	uniform	138	243
81	-77.276	-7.747	1000	1450	uniform	2	25
100	-66.093	-25.01	900	1450	uniform	38	205
101	-77.02	-12.145	1000	1470	uniform	7	26
102	-77.139	-9.373	1100	1470	uniform	8	24
103	-67.892	-17.323	1100	1450	uniform	35	334
104	-65.358	-23.183	1250	1430	uniform	53	129
109	-71.639	-15.614	1150	1450	uniform	90	148
122	-77.024	-12.185	1000	1450	uniform	13	31
135	-66.125	-26.711	1000	1450	uniform	3	47
138	-79.843	-6.518	1100	1400	uniform	1	6
142	-74.94	-14.819	1000	1476	uniform	0	1
148	-76.76	-12.302	1000	1300	uniform	1	4
156	-77.89	-6.01	1000	1450	uniform	67	135
160	-75.036	-14.843	1000	1400	uniform	3	36
181	-79.078	-8.106	1300	1532	uniform	4	25
187	-74.637	-14.809	950	1450	uniform	82	238
193	-70.252	-15.828	1200	1400	uniform	2	10
91	-71.615	-15.61	1000	1532	uniform	10	18
58	-77.858	-6.936	900	1532	uniform	14	114
66	-72.441	-13.226	800	1532	uniform	37	225
85	-76.383	-12.007	1100	1532	uniform	75	175
25	-68.21	-22.92	1300	1400	uniform	3	36
166	-65.359	-23.484	1000	1532	uniform	2	35
106	-75.588	-11.737	1350	1532	uniform	4	36
26	-68.21	-22.92	1400	1532	uniform	8	208
65	-72.541	-13.159	1400	1532	uniform	3	62
68	-71.848	-13.544	1400	1532	uniform	50	219
92	-77.85	-6.934	1400	1485	uniform	5	23
105	-77.079	-12.069	1470	1532	uniform	11	241
110	-76.876	-12.074	1470	1532	uniform	14	84

123	-76.9	-12.255	1470	1532	uniform	15	47
149	-77.099	-12.07	1470	1532	uniform	0	12
155	-75.539	-11.523	1450	1532	uniform	8	14
159	-76.888	-12.03	1400	1532	uniform	32	138
182	-79.472	-7.292	1400	1532	uniform	2	43

code S1: Data Generation and Analysis

Kurt M. Wilson & Weston C. McCool

2025-03-24

- Data Generation
  - Step 1. Terrestrial Climate Data
  - Step 2. Oceanic Climate Data
  - Step 3. Individual Trauma Data
  - Step 4. Population Density
- Analysis
  - Step 1. Checking the Data
  - Step 2. Prep Model Analysis
  - Step 3. Random Forest Model Analysis
  - Step 4. Analyse Model Output
  - Sociopolitical Complexity Interaction
- Taphonomic Correction

This is the supplemental analytical code and data generation for the manuscript "Climate stability and low population pressure predict peaceful interactions over 10,000 years of Central Andean history" by Weston C. McCool, Kurt M. Wilson, Elizabeth Arkush, Daniel A. Contreras, and Brian F. Codding. For questions about the main text, please contact Weston C. McCool at weston.mccool@anthro.utah.edu. For questions about the code, please contact Kurt M. Wilson at kurt.wilson@lawrence.edu.

Load the necessary Libraries

```
library(terra)#working with raster climate data
library(doParallel)#parallel processing for resampling & RF analyses
library(foreach)#parallel processing for resampling & RF analyses
library(arrow)#use for large databases
library(rcarbon)#14c date calibration & KDE generation
library(data.table)#faster data table functions
library(corrplot)#check for autocorrelation between predictor vars
library(fst)#file writing and reading for analysis
library(ranger)#random forest analysis
library(pROC)#calculating precision recall AUC
library(pdp)#predicted partial response
library(ape)#Moran I spatial autocorrelation
library(viridis)#color-blind safe plotting color palettes
library(scales)#working with alpha transparency in plotting
library(sf)#working with spatial data
library(yardstick)#model performance evaluation
library(dplyr)#data manipulation
library(ggplot2)#plotting 3 dimensional interaction plots
library(elevatr)#extract elevation in masl
library(caTools)#split function for test / train splits
library(funModeling)#use in making taphonomic correction function
```

Data Generation

Step 1. Terrestrial Climate Data

We make use of the CHELSA spatially downscaled climate simulation data (1km resolution) available from https://chelsa-climate.org with data downloaded at https://envicloud.wsl.ch/#/?prefix=chelsa%2Fchelsa_V2%2FGLOBAL%2F (Karger et al. 2020, 2023). For this we downloaded the weblink filepaths and used those to read the tif files for climate per century into R directly from the internet. The filepaths download as a text file when multiple filepaths are chosen at once.

We begin by getting the temperature data, first putting the text filepaths into a csv to work with.

```
terra.bio1.paths <- readLines("./data/Bio1_paths.txt")

#make them a dataframe
terra.bio1.pth.df <- data.frame(path = terra.bio1.paths)

#write to csv
write.csv(terra.bio1.pth.df, "./data/Bio1_paths.csv")

#and in the CSV extract the file no and add the year (CE/BCE) and yBP for each file. Now I can save the tif files
using the file number and it will be correctly related to the year.
```

Next we can use the filepaths to download the 117 tif files we need for temperature century scale observations.

```
#Read in the weblink paths for each tif file from CHELSA for 10k to 0 yBP because can read the data in from the C
HELSA website directly without downloading it first
terra.bio1.paths <- read.csv("./data/Bio1_paths.csv")

#run a for loop to get all of the CHELSA tif files for bio1 (temperature)
for (i in 1:length(terra.bio1.paths$file.no)) {
  tif.terra <- rast(terra.bio1.paths$path[i])#read in the tif from CHELSA website
  tif.terra.crop <- crop(tif.terra, y = ext(-82,-62,-28,0))#crop to our study area
  tif.name <- paste("./data/Bio1_temp_tifs/", terra.bio1.paths$file.no[i], ".tif", sep="")
  writeRaster(tif.terra.crop, filename = tif.name)  #save with a unique name
}
```

Having run this code, we have all 117 tif files to get from 10k BCE to 0 CE. Now we want to link these as a raster stack by time.

```
#get the list of all of the temp tif files
fn <- list.files(path = "./data/Bio1_temp_tifs/",
                 pattern = "tif",
                 full.names = FALSE)#obtain a list of all of the files (individual tifs) in the folder and place
them in a single vector

#make into a dataframe
fn <- data.frame(file.no = fn)

#order the dataframe to be decreasing so taht the tif files are in proper order. The proper order can be verified
using the CHELSA technical specifications document downloadable here https://chelsa-climate.org/downloads/
fn <- fn[order(fn$file.no, decreasing = TRUE),]

#Write the file names out to a CSV file because ordering didn't quite work and manually reorder so the names go f
rom 17 down to -99
write.csv(fn, "./data/tiffnames.csv")

#read the fine names into R
Bio1_fn <- read.csv("./data/Bio1_tiffnames.csv")

#make the object just the tif.no character string
Bio1_fn <- Bio1_fn$tif.no

#record this as a full filepath with a link to the directory where the tif files are stored
Bio1_filepath <- paste("./data/Bio1_temp_tifs/", Bio1_fn, sep="")

#Read in all of the tif files as a raster stack (1 raster with 117 layers)
Bio1_tiffs <- rast(lapply(Bio1_filepath, terra::rast))

#Write this new raster as its own file
writeRaster(Bio1_tiffs, "./data/Bio1_temp.tif")
```

Repeat these steps for bio 12 (precipitation). First work with the text filepaths.

```
terra.bio12.paths <- readLines("./data/Bio12_paths.txt")

#make them a dataframe
terra.bio12.pth.df <- data.frame(path = terra.bio12.paths)

#write to csv
write.csv(terra.bio12.pth.df, "./data/Bio12_paths.csv")

#and in the CSV extract the file no and add the year (CE/BCE) and yBP for each file. Now I can save the tif files
using the file number and it will be correctly related to the year.
```

Then get the tif files themselves

```
#Read in the weblink paths for each tif file from CHELSA for 10k to 0 yBP because can read the data in from the C
HELSA website directly without downloading it
terra.bio12.paths <- read.csv("./data/Bio12_paths.csv")

for (i in 1:length(terra.bio12.paths$file.no)) {
  tif.terra <- rast(terra.bio12.paths$path[i])#read in the tif from CHELSA website
  tif.terra.crop <- crop(tif.terra, y = ext(-82,-62,-28,0))#crop to our study area
  tif.name <- paste("./data/Bio12_precip_tifs/", terra.bio12.paths$file.no[i], ".tif", sep="")
  writeRaster(tif.terra.crop, filename = tif.name)  #save with a unique name
}
```

We now have all 117 tif files for precipitation to get from 10k BCE to 0 CE. Now we want to link these as a raster stack by time.

```
fn <- list.files(path = "./data/Bio12_precip_tifs/",
                 pattern = "tif",
                 full.names = FALSE)#obtain a list of all of the files (individual tifs) in the folder and place
them in a single vector

fn <- data.frame(file.no = fn)

fn <- fn[order(fn$file.no, decreasing = TRUE),]

write.csv(fn, "./data/Bio12_tiffnames.csv")#manually reorder so the names go from 17 down to -99

#read the fine names into R
Bio12_fn <- read.csv("./data/Bio12_tiffnames.csv")

#make the object just the tif.no character string
Bio12_fn <- Bio12_fn$tif.no

#record this as a full filepath with a link to the directory where the tif files are stored
Bio12_filepath <- paste("./data/Bio12_precip_tifs/", Bio12_fn, sep="")

#Read in all of the tif files as a raster stack (1 raster with 117 layers)
Bio12_tiffs <- rast(lapply(Bio12_filepath, terra::rast))

#Save the newly created raster file
writeRaster(Bio12_tiffs, "./data/Bio12_precip.tif")
```

Read in the full raster stack for temp & precip

```
Bio1_tifstack <- rast("./data/Bio1_temp.tif")
Bio12_tifstack <- rast("./data/Bio12_precip.tif")
```

We can plot a couple of panels to see how these look.

```
par(mfrow = c(1,3))
plot(Bio1_tifstack$`CHELSA_TraCE21k_bio01_-10_V1.0`)#3000 bp
text(x = -78, y = -27, labels = "3000 yBP", cex = 0.75)
plot(Bio1_tifstack$CHELSA_TraCE21k_bio01_5_V1.0)#1500bp
text(x = -78, y = -27, labels = "1500 yBP", cex = 0.75)
mtext("Temperature", side = 3, line = 0)
plot(Bio1_tifstack$CHELSA_TraCE21k_bio01_15_V1.0)#500bp
text(x = -78, y = -27, labels = "500 yBP", cex = 0.75)
```

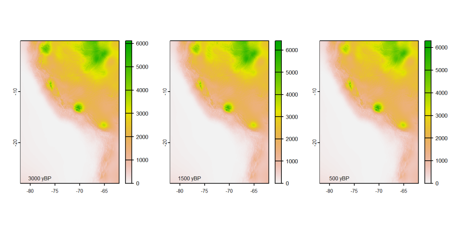


```
par(mfrow = c(1,3))
plot(Bio12_tifstack$`CHELSA_TraCE21k_bio12_-10_V1.0`)#3000 bp
text(x = -78, y = -27, labels = "3000 yBP", cex = 0.75)
plot(Bio12_tifstack$CHELSA_TraCE21k_bio12_5_V1.0)#1500bp
text(x = -78, y = -27, labels = "1500 yBP", cex = 0.75)
mtext("Precipitation", side = 3, line = 0)
plot(Bio12_tifstack$CHELSA_TraCE21k_bio12_15_V1.0)#500bp
text(x = -78, y = -27, labels = "500 yBP", cex = 0.75)
```

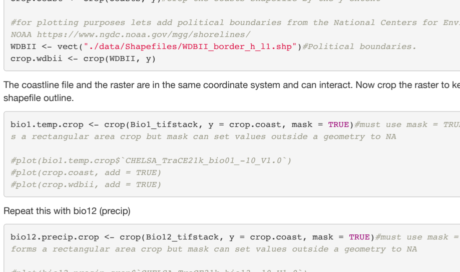

Supplemental Figures 1 & 2. Example CHELSA climate reonstructions of temperature and precipitation spatially at 3k yBP, 1.5k yBP, and 0.5k yBP.

Now that we have the rasterstack tif files, we want to: 1) NA out any cell values that are in the oceans because we don't want to use that temp/precip value for individuals who live near the coasts - we just want terrestrial data. 2) Sample the raster for appropriate date for each individual. Repeat for each site. 1k repetitions each.

```
#This uses global coastal shapefiles from the National Centers for Environmental Information via NOAA https://ww
w.ngdc.noaa.gov/mgg/shorelines/
coasts <- vect("./data/Shapefiles/GSHHS_l_L1.shp")
y <- ext(-85,-50,-35,5)#xmin, xmax, ymin, ymax. Set a y extent that captures the study area.
crop.coast <- crop(coasts, y)#crop the coasts shapefile by the y extent

#for plotting purposes lets add political boundaries from the National Centers for Environmental Information via
NOAA https://www.ngdc.noaa.gov/mgg/shorelines/
WDBII <- vect("./data/Shapefiles/WDBII_border_h_l1.shp")#Political boundaries.
crop.wdbii <- crop(WDBII, y)
```

The coastline file and the raster are in the same coordinate system and can interact. Now crop the raster to keep only the values inside the coast shapefile outline.

```
bio1.temp.crop <- crop(Bio1_tifstack, y = crop.coast, mask = TRUE)#must use mask = TRUE because crop only perform
s a rectangular area crop but mask can set values outside a geometry to NA

#plot(bio1.temp.crop$`CHELSA_TraCE21k_bio01_-10_V1.0`)
#plot(crop.coast, add = TRUE)
#plot(crop.wdbii, add = TRUE)
```

Repeat this with bio12 (precip)

```
bio12.precip.crop <- crop(Bio12_tifstack, y = crop.coast, mask = TRUE)#must use mask = TRUE because crop only per
forms a rectangular area crop but mask can set values outside a geometry to NA

#plot(bio12.precip.crop$`CHELSA_TraCE21k_bio12_-10_V1.0`)
#plot(crop.coast, add = TRUE)
#plot(crop.wdbii, add = TRUE)
```

Let's write the masked rasters to new objects to avoid the amount of time it takes to do the masking procedure

```
writeRaster(bio1.temp.crop, "./data/Bio01_temp_mask.tif")

writeRaster(bio12.precip.crop, "./data/Bio12_precip_mask.tif")
```

Read in the written rasters

```
bio1.temp.crop <- rast("./data/Bio01_temp_mask.tif")
bio12.precip.crop <- rast("./data/Bio12_precip_mask.tif")

#Plot to see the difference
par(mfrow = c(1,3))
plot(bio1.temp.crop$`CHELSA_TraCE21k_bio01_-10_V1.0`)#3000 bp
text(x = -78, y = -27, labels = "3000 yBP", cex = 0.75)
plot(bio1.temp.crop$CHELSA_TraCE21k_bio01_5_V1.0)#1500bp
text(x = -78, y = -27, labels = "1500 yBP", cex = 0.75)
mtext("Temperature", side = 3, line = 0)
plot(bio1.temp.crop$CHELSA_TraCE21k_bio01_15_V1.0)#500bp
text(x = -78, y = -27, labels = "500 yBP", cex = 0.75)
```

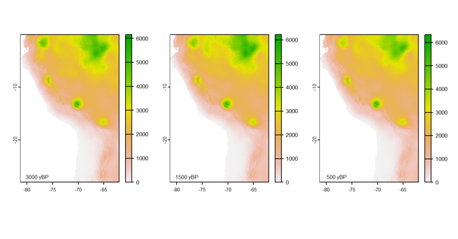


```
par(mfrow = c(1,3))
plot(bio12.precip.crop$`CHELSA_TraCE21k_bio12_-10_V1.0`)#3000 bp
text(x = -78, y = -27, labels = "3000 yBP", cex = 0.75)
plot(bio12.precip.crop$CHELSA_TraCE21k_bio12_5_V1.0)#1500bp
text(x = -78, y = -27, labels = "1500 yBP", cex = 0.75)
mtext("Precipitation", side = 3, line = 0)
plot(bio12.precip.crop$CHELSA_TraCE21k_bio12_15_V1.0)#500bp
text(x = -78, y = -27, labels = "500 yBP", cex = 0.75)
```

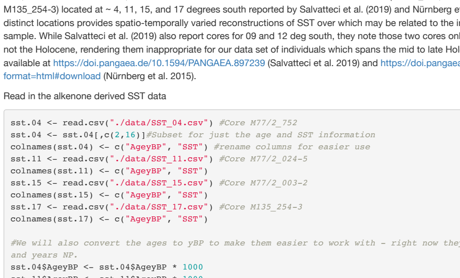

Supplemental Figures 3 & 4. CHELSA climate rasters at 3k yBP, 1.5k yBP, and 0.5k yBP for Temperature and Precip cropped to just the land areas of the study.

Step 2. Oceanic Climate Data

For our coastal analysis portions we will want reconstructions of sea surface temperature (SST). For both coastal and non-coastal individuals we will want El Nino Southern Oscillation frequency estimate data. This is because SST impacts marine resource availability and heterogeneity (see Wilson et al 2023 for a summary) which we expect to impact peace / violence on the coasts. ENSO impacts both the coastal and non-coastal environments through bringing precipitation to some areas and starving others of precip. These teleconnections across the elevational gradient of the Andes are documented and expected to impact people's options and decision-making.

First, for SST reconstructions, we use alkenone based SST estimates from four ocean cores (M77/2_757, M77/2_024-5, M77/2_003-2, and M135_254-3) located at ~ 4, 11, 15, and 17 degrees south reported by Salvatteci et al. (2019) and Nürnberg et al. (2015). Using cores from four distinct locations provides spatio-temporally varied reconstructions of SST over which may be related to the individuals in the stable isotope sample. While Salvatteci et al. (2019) also report cores for 09 and 12 deg south, they note those two cores only capture the de-glacial period and not the Holocene, rendering them inappropriate for our data set of individuals which spans the mid to late Holocene. The data for these cores are available at https://doi.pangaea.de/10.1594/PANGAEA.897239 (Salvatteci et al. 2019) and https://doi.pangaea.de/10.1594/PANGAEA.848848?format=html#download (Nürnberg et al. 2015).

Read in the alkenone derived SST data

```
sst.04 <- read.csv("./data/SST_04.csv") #Core M77/2_752
sst.04 <- sst.04[,c(2,16)]#Subset for just the age and SST information
colnames(sst.04) <- c("AgeyBP", "SST") #rename columns for easier use
sst.11 <- read.csv("./data/SST_11.csv") #Core M77/2_024-5
colnames(sst.11) <- c("AgeyBP", "SST")
sst.15 <- read.csv("./data/SST_15.csv") #Core M77/2_003-2
colnames(sst.15) <- c("AgeyBP", "SST")
sst.17 <- read.csv("./data/SST_17.csv") #Core M135_254-3
colnames(sst.17) <- c("AgeyBP", "SST")

#We will also convert the ages to yBP to make them easier to work with - right now they are in decimals for thous
and years NP.
sst.04$AgeyBP <- sst.04$AgeyBP * 1000
sst.11$AgeyBP <- sst.11$AgeyBP * 1000
sst.15$AgeyBP <- sst.15$AgeyBP * 1000
sst.17$AgeyBP <- sst.17$AgeyBP * 1000
```

To estimate ENSO, we add the Laguna Pallcacocha core-based proxy record from Ecuador to track the frequency of ENSO events per century (Moy et al. 2002). The record extends back ~14,000 years and is available here https://www.ncei.noaa.gov/access/paleo-search/).

```
#read in the ENSO data
enso.dat <- read.csv("./data/Moy_et_al.csv")
colnames(enso.dat)[1] <- "AgeyBP" #Change the name so it can be used in the sampling loop below
```

Step 3. Individual Trauma Data

The next steps are to get individual data setup.

```
#read in the individual data
indv.data <- read.csv("./data/indv_data.csv")
```

Plot the spatial variation in temp and precip at 1000 yBP with individuals over top to see the variation we may have.

```
#get the unique individual locations
indvs.loc <- indv.data[!duplicated(indv.data$Site.and.location),]

temp1k <- bio1.temp.crop$`CHELSA_TraCE21k_bio01_10_V1.0`
prcp1k <- bio12.precip.crop$`CHELSA_TraCE21k_bio12_10_V1.0`
#for visualization purposes, we set all cells over 2000 mm / yr precip to be 2000 mm. This is only in the plots b
elow - not the analysis.
prcp1k <- clamp(prcp1k, lower = 0, upper = 2000, values = TRUE)

par(pty = "s")
terra::plot(temp1k, col = inferno(50, begin = 0.10), xlim = c(-89,-62), ylim = c(-27,0), main = "Temperature 1k y
BP")
plot(crop.coast, add = TRUE, border = "grey40")
plot(crop.wdbii, add = TRUE, col = "grey40")
points(x=indvs.loc$Longitude, y=indvs.loc$Latitude, pch = 21, bg = "white", col = "black")
points(x =-81.320500, y = -3.950167, pch = 17, col = "black")
points(x =-71.769000, y = -17.420500, pch = 17, col = "black")
points(x =-75.688000, y = -15.103500, pch = 17, col = "black")
points(x =-78.015200, y = -11.083500, pch = 17, col = "black")
points(x =-79.23333, y = -2.76667, pch = 15, col = "black")
```

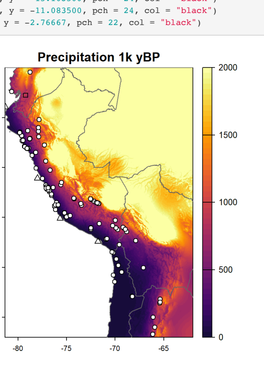


```
#par(pty = "s")
terra::plot(prcp1k, col = inferno(50, begin = 0.10), xlim = c(-89,-62), ylim = c(-27,0), main = "Precipitation 1k
yBP")
plot(crop.coast, add = TRUE, border = "grey40")
plot(crop.wdbii, add = TRUE, col = "grey40")
points(x=indvs.loc$Longitude, y=indvs.loc$Latitude, pch = 21, bg = "white", col = "black")
points(x =-81.320500, y = -3.950167, pch = 24, col = "black")
points(x =-71.769000, y = -17.420500, pch = 24, col = "black")
points(x =-75.688000, y = -15.103500, pch = 24, col = "black")
points(x =-78.015200, y = -11.083500, pch = 24, col = "black")
points(x =-79.23333, y = -2.76667, pch = 22, col = "black")
```

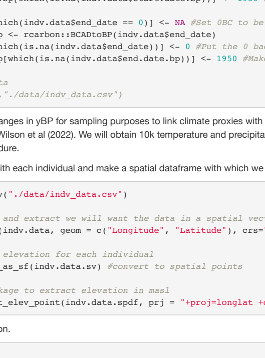

Supplemental Figures 5 & 6. Spatial variation in temperature and precipitation at 1k yBP with the trauma sample locations plotted in white circles. On the temperature map (Fig 5), SST cores are black triangles and the ENSO core is a black square. On the precipitation map (Fig 6), SST cores are white triangles and the ENSO core is a white square.

The CHELSA data is in yBP, but the peace data is in BCE/CE. We can do a quick conversion to make sure both datasets are in the same units.

```
#repeat this for the individuals
indv.data <- indv.data[which(!is.na(indv.data$start_date)),]#Drop individuals for whom we have no date range
indv.data$start_date[which(indv.data$start_date == 0)] <- NA #Set 0BC to be NA b/c rcarbon can't handle the 0
indv.data$start.date.bp <- rcarbon::BCADtoBP(indv.data$start_date)
indv.data$start_date[which(is.na(indv.data$start_date))] <- 0 #Put the 0 back in
indv.data$start.date.bp[which(is.na(indv.data$start.date.bp))] <- 1950 #Make 0 BCAD 1950 yBP

indv.data$end_date[which(indv.data$end_date == 0)] <- NA #Set 0BC to be NA b/c rcarbon can't handle the 0
indv.data$end.date.bp <- rcarbon::BCADtoBP(indv.data$end_date)
indv.data$end_date[which(is.na(indv.data$end_date))] <- 0 #Put the 0 back in
indv.data$end.date.bp[which(is.na(indv.data$end.date.bp))] <- 1950 #Make 0 BCAD 1950 yBP

#Save the updated data
#write.csv(indv.data,"./data/indv_data.csv")
```

Now we can use the date ranges in yBP for sampling purposes to link climate proxies with sites and individuals. We will do this adapting the procedure documented in Wilson et al (2022). We will obtain 10k temperature and precipitation estimates for each site and for each individual in a site via a resampling procedure.

First, read in the data set with each individual and make a spatial dataframe with which we extract elevation.

```
indv.data <- read.csv("./data/indv_data.csv")

#To work with buffer and extract we will want the data in a spatial vector format
indv.data.sv <- vect(indv.data, geom = c("Longitude", "Latitude"), crs="+proj=longlat +datum=WGS84")

#we can also extract elevation for each individual
indv.data.spdf <- st_as_sf(indv.data.sv) #convert to spatial points

#use the elevatr package to extract elevation in masl
indv.data.spdf <- get_elev_point(indv.data.spdf, prj = "+proj=longlat +datum=WGS84 +no_defs", src = "aws")
```

Plot the elevation distribution.

```
par(pty = "s")
hist(indv.data.spdf$elevation, xlab = "Elevation (masl)", main = "")
```

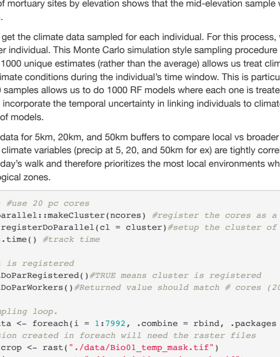

Supplemental Figure 7. Histogram of the elevation for individuals. This suggests we have a coastal cluster of individuals and then an inland / highland cluster. This helps inform our split of the individuals into a coastal and inland category. The zone partition (coast/inland) is designed to: 1) differentiate demographic estimates between coastal and inland zones (Wilson et al. 2022 show that coastal and inland population estimates differ), and 2) provide flexibility to include or exclude Sea Surface Temperature (SST) and ENSO frequency variables from inland sites. For each skeletal sample, we will estiamte local climate conditions within a 5 km buffer (with similar analyses conducted for 20 km and 50 km buffers, which all significantly covary - see below) to capture the variation across elevation and ecological gradients. Introducing a mid-elevation zone would not affect estimates for terrestrial climate, oceanic climate, or socio-political complexity. It would however require generating additional population estimates for the mid-elevation zone. As Wilson et al. (2022, Nat. Sci. Rep.) demonstrated, population estimates for mid-elevation and highland zones broadly co-vary. Based on this, we determine that creating a single elevation partition was appropriate. Importantly, this approach reduces computational demands (analysis requires renting time on the burdened University of Utah high performance supercomputer) and avoids generating an excessive number of interpretive plots that would need to be included in the main text, which would decrease interpretability. Finally, the plot of mortuary sites by elevation shows that the mid-elevation sample would contain a small relative sample size, which may in itself cause overfitting.

Now we want to get the climate data sampled for each individual. For this process, we will also implement parallel processing. We want to get 1000 samples per individual. This Monte Carlo simulation style sampling procedure captures the possible suite of climate conditions for each indvidual. Using 1000 unique estimates (rather than the average) allows us treat climate as a probability distribution, with more samples at the most frequent climate conditions during the individual's time window. This is particularly needed as these individuals are not radiocarbon dated. Keeping all 1000 samples allows us to do 1000 RF models where each one is treated as 'true' and to evaluate the aggregate output of 1000 models that fully incorporate the temporal uncertainty in linking individuals to climate. Patterns that exist in spite of this uncertainty are consistent across the suite of models.

Here we extract data for 5km, 20km, and 50km buffers to compare local vs broader climate. Correlation testing (further below) demonstrates the 5, 20, and 50km climate variables (precip at 5, 20, and 50km for ex) are tightly correlated. In all analyses we only use the 20km data as this represents ~1-2 day's walk and therefore prioritizes the most local environments while accounting for local mobility and accessing nearby elevational ecological zones.

```
ncores <- 20 #use 20 pc cores
cluster <- parallel::makeCluster(ncores) #register the cores as a cluster
doParallel::registerDoParallel(cl = cluster)#setup the cluster of cores for use in parallel processing
start <- Sys.time() #track time

#check if it is registered
foreach::getDoParRegistered()#TRUE means cluster is registered
foreach::getDoParWorkers()#Returned value should match # cores (20 in this case)

#Now the sampling loop.
indv.clim.data <- foreach(i = 1:7992, .combine = rbind, .packages = "terra") %dopar% { #7992 individuals
  #each session created in foreach will need the raster files
  bio1.temp.crop <- rast("./data/Bio01_temp_mask.tif")
  bio12.precip.crop <- rast("./data/Bio12_precip_mask.tif")
  #each session needs the indv data
  indv.data <- read.csv("./data/indv_data.csv")
  #select the focal indv for this iteration
  indv.data <- indv.data[i,]
  #To work with buffer and extract we will want the data in a spatial vector format
  indv.data.sv <- vect(indv.data, geom = c("Longitude", "Latitude"), crs="+proj=longlat +datum=WGS84")
  #each session needs the SST and ENSO data. Here SST information comes from Salvatecci et al 2019, Nurnberg et a
l 2015 and the ENSO data is from Moy et al 2002
  sst.04 <- read.csv("./data/SST_04.csv") #Core M77/2_752
  sst.04 <- sst.04[,c(2,16)]#Subset for just the age and SST information
  colnames(sst.04) <- c("yearBP", "SST") #rename columns for easier use
  sst.11 <- read.csv("./data/SST_11.csv") #Core M77/2_024-5
  colnames(sst.11) <- c("yearBP", "SST")
  sst.15 <- read.csv("./data/SST_15.csv") #Core M77/2_003-2
  colnames(sst.15) <- c("yearBP", "SST")
  sst.17 <- read.csv("./data/SST_17.csv") #Core M135_254-3
  colnames(sst.17) <- c("yearBP", "SST")
  enso.dat <- read.csv("./data/Moy_et_al.csv")#Moy Laguna Pallcacocha ENSO frequency data
  colnames(enso.dat)[1] <- "yearBP" #Change the name so it can be used more easily in the sampling loop below

  #We will also convert the ages to yBP to make them easier to work with - right now they are in decimals for tho
usand years.
  sst.04$yearBP <- sst.04$yearBP * 1000
  sst.11$yearBP <- sst.11$yearBP * 1000
  sst.15$yearBP <- sst.15$yearBP * 1000
  sst.17$yearBP <- sst.17$yearBP * 1000

  #get the correct SST core for the individual
    ifelse(indv.data$Latitude >= -7.5, SSTcore <- sst.04, ifelse(indv.data$Latitude <= -7.5 & indv.data$Latitude
> -12, SSTcore <- sst.11, ifelse(indv.data$Latitude <= -12 & indv.data$Latitude > -16, SSTcore <- sst.15, SSTcore
<- sst.17)))

  #for Late Horizon individuals with data between 550 and 418 ybp, we need to only use the cores that have data e
xtending that late in time.
    #the sst11 & 15 cores are lacking data after ~650 yBP. So we need later period sites near
    #those two cores to use either the sst4 or sst17 cores as the nearest proxy
    if (indv.data$end.date.bp == 418 & indv.data$start.date.bp <= 651) {ifelse(indv.data$Latitude > -12, SSTcore
<- sst.04, SSTcore <- sst.17)} #Since we only have 2 cores, we split individuals as those north of 12 degree sout
h and those south of that latitude.

  #Get temp data for each individual.
  mnt.5km <- terra::extract(bio1.temp.crop, y = terra::buffer(indv.data.sv, width = 5000), fun = mean, na.rm = TR
UE)
  mnt.20km <- terra::extract(bio1.temp.crop, y = terra::buffer(indv.data.sv, width = 20000), fun = mean, na.rm =
TRUE)
  mnt.50km <- terra::extract(bio1.temp.crop, y = terra::buffer(indv.data.sv, width = 50000), fun = mean, na.rm =
TRUE)
  sdt.5km <- terra::extract(bio1.temp.crop, y = terra::buffer(indv.data.sv, width = 5000), fun = sd, na.rm = TRU
E)
  sdt.20km <- terra::extract(bio1.temp.crop, y = terra::buffer(indv.data.sv, width = 20000), fun = sd, na.rm = TR
UE)
  sdt.50km <- terra::extract(bio1.temp.crop, y = terra::buffer(indv.data.sv, width = 50000), fun = sd, na.rm = TR
UE)
  #Get precip data for each individual
  mnp.5km <- terra::extract(bio12.precip.crop, y = terra::buffer(indv.data.sv, width = 5000), fun = mean, na.rm =
TRUE)
  mnp.20km <- terra::extract(bio12.precip.crop, y = terra::buffer(indv.data.sv, width = 20000), fun = mean, na.rm
= TRUE)
  mnp.50km <- terra::extract(bio12.precip.crop, y = terra::buffer(indv.data.sv, width = 50000), fun = mean, na.rm
= TRUE)
  sdp.5km <- terra::extract(bio12.precip.crop, y = terra::buffer(indv.data.sv, width = 5000), fun = sd, na.rm = T
RUE)
  sdp.20km <- terra::extract(bio12.precip.crop, y = terra::buffer(indv.data.sv, width = 20000), fun = sd, na.rm =
TRUE)
  sdp.50km <- terra::extract(bio12.precip.crop, y = terra::buffer(indv.data.sv, width = 50000), fun = sd, na.rm =
TRUE)
  #Make a dataframe with all the climate data for each year
  dat <- data.frame(yearBP = seq(from = 300, to = 11900, by = 100),
                    MNtemp5km = t(mnt.5km[,2:118]),
                    MNtemp20km = t(mnt.20km[,2:118]),
                    MNtemp50km = t(mnt.50km[,2:118]),
                    SDtemp5km = t(sdt.5km[,2:118]),
                    SDtemp20km = t(sdt.20km[,2:118]),
                    SDtemp50km = t(sdt.50km[,2:118]),
                    MNprcp5km = t(mnp.5km[,2:118]),
                    MNprcp20km = t(mnp.20km[,2:118]),
                    MNprcp50km = t(mnp.50km[,2:118]),
                    SDprcp5km = t(sdp.5km[,2:118]),
                    SDprcp20km = t(sdp.20km[,2:118]),
                    SDprcp50km = t(sdp.50km[,2:118]))
  #Subset the climate data just to the site's possible years
  #need to deal with Inca period sites that don't fit the climate data in yBP so grab the data for 500 & 400 yBP
  if (indv.data.sv$end.date.bp == 418 & indv.data.sv$start.date.bp <= 550) {
  clim.dat <- data.frame(subset(dat, yearBP>=400 & yearBP <=500))
  SSTcore <- data.frame(subset(SSTcore, yearBP >=400 & yearBP <= 500, select = SST))
  enso.dat <- data.frame(subset(enso.dat, yearBP >=400 & yearBP <= 500, select = ENSO.no))
  } else {#for all other sites, just use their range
  clim.dat <- data.frame(subset(
          dat, yearBP>=indv.data.sv$end.date.bp & yearBP <= indv.data.sv$start.date.bp))
  SSTcore <- data.frame(subset(
          SSTcore, yearBP>=indv.data.sv$end.date.bp & yearBP <= indv.data.sv$start.date.bp, select = SST))
  enso.dat <- data.frame(subset(
            enso.dat, yearBP >= round(indv.data.sv$end.date.bp, digits = -2) & yearBP <= round(indv.data.sv$star
t.date.bp, digits = -2), select = ENSO.no))
  }
  #Sample the climate data 1k times
  result.clim <- clim.dat[sample(nrow(clim.dat), replace = TRUE, 1000),]
  result.clim$CVprcp5km <- result.clim$SDprcp5km / result.clim$MNprcp5km#get coef variation in precip due to like
ly autocorrelation
  result.clim$CVprcp20km <- result.clim$SDprcp20km / result.clim$MNprcp20km
  result.clim$CVprcp50km <- result.clim$SDprcp50km / result.clim$MNprcp50km
  result.clim$SST <- SSTcore[sample(nrow(SSTcore),replace = TRUE, 1000),]
  result.clim$ENSO <- enso.dat[sample(nrow(enso.dat), replace = TRUE, 1000),]
  rownames(result.clim) <- c()#remove rownames (this is the tif layer name)
  #Replicate the site's information 1k times
  indv.results <- do.call("rbind", replicate(1000,indv.data, simplify = FALSE))
  #Combine the site data with the resampled climate for the site
  indv.results <- cbind(indv.results, result.clim)
  #Put all of this site data into a dataframe that will eventually hold the 1k replicates for each site
  return(indv.results)
  #remove session objects to save RAM
  rm(bio1.temp.crop)
  rm(bio12.precip.crop)
  rm(indv.data)
  rm(indv.data.sv)
  rm(mnt.5km)
  rm(mnt.20km)
  rm(mnt.50km)
  rm(mnp.5km)
  rm(mnp.20km)
  rm(mnp.50km)
  rm(sst.04)
  rm(sst.11)
  rm(sst.15)
  rm(sst.17)
  rm(sdt.5km)
  rm(sdt.20km)
  rm(sdt.50km)
  rm(sdp.5km)
  rm(sdp.20km)
  rm(sdp.50km)
  rm(dat)
  rm(clim.dat)
  rm(SSTcore)
  rm(enso.dat)
  rm(result.clim)
  rm(indv.results)
}

stopCluster(cluster)

end <- Sys.time()#record end time
end - start #check how long it took to run the code

#Write this out as a fst data file for faster read and write speed + use in analysis
write.fst(indv.clim.data, "./data/indv.data.full.fst")

#could also do this with feather via the arrow package
#write_feather(indv.clim.data, "./data/indv.data.resampled.feather", compression = "zstd")

#note - this output will be very large
```

With this done we have 1k climate estimates (precip, temp, temp variability, precip variability, sea surface temperature, and ENSO) per individual at 5, 20, and 50km buffers tied to their spatial location for evaluation of our theoretical predictions.

Step 4. Population Density

Estimating past relative population density is a challenging problem that has been critiqued nearly as widely as it has been employed (see Carleton and Grocutt 2021, Contreras and Codding 2024, Crema 2022, Robinson et al. 2019, McLaughlin et al. 2021, and Codding et al. 2024 for recent reviews, summaries, and overviews of critiques and examples). Around the world scholars have been repeatedly testing and evaluating the assumptions of dates as data - that larger populations in the past will have produced more radiocarbon dateable material than smaller populations leading archaeologists to find more dateable material from periods with larger populations and, proportionally, radiocarbon date more of such material - and its various problems (e.g., spatial inconsistencies in archaeological work, temporal biases in periods of interest, taphonomic preservation concerns, research budget differences between projects, states, and/or countries, determining the appropriate calibration curves per material, inter-laboratory variation in radiocarbon methods inclusive of improved methods over time, and more), finding routinely that it is an imperfect yet useful relative tracker of past population size (e.g., McLaughlin et al. 2021; Robinson et al. 2019; Timpson et al. 2014; Williams et al. 2015; Wirtz et al. 2024; Goebel et al. 2021; Crema 2022; Chaput and Gajewski 2016; Zahid et al. 2016). While direct validations of dates as data are difficult, multiple studies have provided validations that the assumption that more dates will derive from periods with larger past populations holds across multiple validation attempts (Brown 2015; Carleton 2021; Carleton and Grocutt 2021; Contreras and Codding 2024; Contreras and Meadows 2014; Robinson et al. 2021) including with ethnographic population density (Codding et al. 2024). Within the study region, multiple scholars have found the ‘dates as data’ approach an imperfect, though viable, proxy of population in the study region (e.g., Riris 2018; Riris and Arroyo-Kalin 2019; Roscoe et al. 2021; Gayo et al. 2015). We follow these approaches as we have in prior work (Wilson et al. 2022) and use ‘dates as data’ as a marker for relative past population size.

In doing so, we contend that though there are undoubtedly biases affecting dates as data (recently reviewed in Crema 2022 and Contreras and Codding 2024 and mentioned above) they do not overly affect (i.e., skew) the patterns we observe and the best practices we follow add confidence to the utility of the relative patterns we observe. First, a common first step (Rick 1987; Riris 2018; Wilson et al. 2022) in dates as data work in the Central Andes has been partitioning the data into coastal and highland groups (as we do below) to help minimize the effects of differential research attention in space. The eastern slope of the Central Andes and the Peruvian Amazon remain significantly underrepresented in the radiocarbon record relative to the coasts and western slopes and there has been differential emphasis on radiocarbon dating between the coasts and the western slopes. Partitioning the data means that coastal patterns are compared only with other coastal data, balancing the research emphases in space and time - the same for the western slope partition. Though radiocarbon dates are largely absent from the eastern slopes and the Peruvian Amazon so too is trauma data - for the same reasons of research intensity, difficulty, and accessibility - which therefore does not bias our results.

Two other biases for radiocarbon dates as data may be most relevant to our research problem here: differential research attention by period of time, and landscape taphonomy. We address landscape taphonomy more below. With respect to differential research attention, until recently less dating attention has focused on time periods considered to have robust material culture chronologies (see Contreras 2022), and research attention has arguably focused more on “horizons” than “intermediate periods” - however sample sizes for some archaeological data such as stable isotopes (Wilson et al. 2022) and trauma (this study) are largest in the Late Intermediate Period. Despite this potential underrepresentation of later period, the dates as data population patterns match to the population pattern expectations for both the coastal and non-coastal regions (see below), suggesting larger populations later in time than earlier despite potential biases that arguably would favor the inverse pattern. As dates as data represent relative, not absolute, population sizes, in consequence, we presume that if anything we are underestimating absolute population densities from later periods (the last 2-3kya) but we still capture the relative pattern of larger populations later in time.

The first step towards evaluating appropriateness (below) is building the population density proxy. For this we will use a kernel density estimate version of the dates as data approach following Wilson et al. (2022), creating 1000 different, resampled, kernel desnity estimates that capture the uncertainty in possible reconstructions of past relative population. For additional review of KDE use see Crema (2022) and Brown (2017). These 1000 KDEs are then resampled 1000 times for each individual to provide an estimate of the experienced, relative, past population size for each individual that captures the range of possibilites (e.g., uncertainty) in reconstruction.

The 14c data comes from Dr. Daniel Contreras’ database building efforts.

```
#read in the data
Andes_14c <- read_csv_arrow("./data/CentralAndes14C_alldates.csv")

Andes_14c <- Andes_14c[which(!is.na(Andes_14c$C14_age) & !is.na(Andes_14c$C14_error)),]#Drop several dates that don't have a known C14age or error

Andes_14c <- Andes_14c[which(Andes_14c$C14_error < 101 & Andes_14c$C14_error >= 1),]#Rely only on dates with errors 100 or less for date quality purposes

#drop dates without coordinates since we need to be able to establish coastal / non-coastal
Andes_14c <- Andes_14c[which(!is.na(Andes_14c$Lat)),]
Andes_14c <- Andes_14c[which(!is.na(Andes_14c$Lon)),]
```

Now we need to be able to categorize each date as coastal or non-coastal to create distinct population density reconstructions. This is because our analysis runs coastal and non-coastal separately (see below). We generate coastal and non-coastal relative population sizes as spatial aggregates rather than disaggregating further in space to emphasize the temporal patterns, and uncertainty, in past population. The further and further a set of radiocarbon dates is disaggregated in space, the more susceptible the pattern may be to various biases (Carleton and Groucutt 2021), whereas across large spatiotemporal areas dates as data has proven robust (see Codding et al. 2024; Crema and Bevan 2021; Crema 2022; Carleton and Groucutt 2021; DiNapoli et al. 2021; Robinson et al. 2019 for recent reviews). This is because, at our analytical spatial extent, increased populations in one area at one point in time typically correspond with increased populations in other, similar, areas at that point in time even if not all areas have been heavily studied archaeologically (and vice versa). The problem of space in dates as data is well known with established methods for attempting to generate local, spatial, reconstructions (e.g., Crema et al. 2017; Chaput et al. 2015) which includes aggregating to larger spatial zones that minimizes sampling error (see Crema 2022; Hinz 2020) based on two assumptions: a) typically, regionally, increased populations in one area at one point in time correspond with increased populations in other, similar, areas at that point in time even if not all areas have been heavily studied archaeologically (and vice versa) and b) large sample sizes across large spatial extents can aggregate out some of the regional biases.

```
#Here we split the dates as coastal or non-coastal for recreating the population estimates for each region.
coasts <- vect("./data/Shapefiles/GSHHS_l_L1.shp")
y <- ext(-85,-50,-35,5)#xmin, xmax, ymin, ymax. Set a y extent that captures the study area.
crop.coast <- crop(coasts, y)#crop the coasts shapefile by the y extent

#for plotting purposes lets add political boundaries
WDBII <- vect("./data/Shapefiles/WDBII_border_h_l1.shp")#Political boundaries.
crop.wdbii <- crop(WDBII, y)

dem <- rast("./data/DEM/PnCB_DEM.tif")#read the new geotiff file in. This is a DEM of Peru, North Chile, and Bolivia constructed from Shuttle imagery from NASA.

points_14c <- matrix(c(Andes_14c$Lon, Andes_14c$Lat), ncol = 2)#make a matrix of points
elevation_14c <- extract(x = dem, y = points_14c, method = "simple")#use the extract function to obtain the elevation - meters above sea level - for each site

Andes_14c <- cbind(Andes_14c, elevation_14c)#add the elevations to the isotope dataframe
colnames(Andes_14c)[16] <- "elev.masl"

#write this out for easier use
#write.csv(Andes_14c, "./data/Andes_14c_CoHi.csv")

#read in the data
Andes_14c <- read.csv("./data/Andes_14c_CoHi.csv")

crop_ext <- ext(dem)#extract the DEM extents to crop the coast shapefile to the DEM extents
```

Next we need to crop the coast shapefile to the extent of the DEM for generating 14c dates’ distance to coast, calculate distance to coast, and categorize dates as coastal or non-coastal.

```
coasts_crop <- st_as_sf(coasts)
coasts_crop <- st_crop(coasts_crop, crop_ext)#crop the coast shapefile to the DEM extents
coasts_crop_line <- st_cast(coasts_crop, "LINESTRING")#convert from polygon to linestring because otherwise we calculate distance from points to a space within the polygon (inherently 0).
dates_sf <- st_as_sf(Andes_14c, coords = c("Lon", "Lat"), crs = 4326)#convert the isodat_test dataframe to a simple feature collection (sfc)
dist2coast.dates <- st_distance(coasts_crop_line, dates_sf)#compute euclidean distance (m) between the lines in the linestring coasts2_crop_line file and the site points as a spatial file
dist2coast_dates.df <- as.data.frame(dist2coast.dates)#Produce a dataframe with the output of the distance calculations. We only care about the 1st distance calculation for each site (beacuse this is the distance to the bounding line). The other 245 calculations are to islands and other anomolies, not the direct coastline.
dist.dates <- dist2coast_dates.df[1,]#select only the distance to the first object (the coastline)
dist.dates <- t(dist.dates)#transpose so that there is 1 column with the distance and a row for each individual
Andes_14c <- cbind(Andes_14c, dist.dates)#add the distance to coast measure to the dataframe as a new column
colnames(Andes_14c)[17] <- "dist2coast"#rename the distance to coast column as dist2coast
Andes_14c$d2coast_km <- Andes_14c$dist2coast/1000 #convert to km

#Make a factor variable for coastal vs non-coastal dates
Andes_14c$elev.factor <- "Coastal" #set all dates to be coastal. Dates from lower than 500 masl and less than 15km from the coast should be left as coastal. We use 500 masl as that is the traditional definition of the costa region from Purra Vidal
Andes_14c$elev.factor[Andes_14c$elev.masl <= 500 & Andes_14c$d2coast_km > 15] <- "Non-Coastal" #Dates from lower than 500 masl but greater than 15km from the coast should be mid-elevation
Andes_14c$elev.factor[Andes_14c$elev.masl >= 500] <- "Non-Coastal"#Dates existing above 500 masl and below 3500 masl should be mid-elevation

#Write the data to a new csv for easy use.
write.csv(Andes_14c, "./data/Andes_14c_CoHi.csv")#write a new dataframe that leaves out sites for which we do not have gps points and includes elevations
```

Now we can calibrate the radiocarbon dates will use.

```
#read in the 14c data
Andes_14c <- read.csv("./data/Andes_14c_CoHi.csv")

#Calibrate the dates separately for coast and non-coast
Coast_14c <- Andes_14c[which(Andes_14c$elev.factor == "Coastal"),]
NCoast_14c <- Andes_14c[which(Andes_14c$elev.factor == "Non-Coastal"),]

dat_cal.C <- calibrate(Coast_14c$C14_age,
                      errors = Coast_14c$C14_error,
                      calCurves = Coast_14c$CalCurve,
                      resOffsets = Coast_14c$Res_Offset,
                      resErrors = Coast_14c$Res_Offset_Error,
                      ids = Coast_14c$LabID_conform,
                      verbose = FALSE)

dat_cal.NC <- calibrate(NCoast_14c$C14_age,
                      errors = NCoast_14c$C14_error,
                      calCurves = NCoast_14c$CalCurve,
                      resOffsets = NCoast_14c$Res_Offset,
                      resErrors = NCoast_14c$Res_Offset_Error,
                      ids = NCoast_14c$LabID_conform,
                      verbose = FALSE)
```

```
#run the coastal binsense assessment
binsense(dat_cal.C, y = Coast_14c$Site, h = c(0, 100, 200, 500), timeRange = c(10000, 0), verbose = FALSE)
```

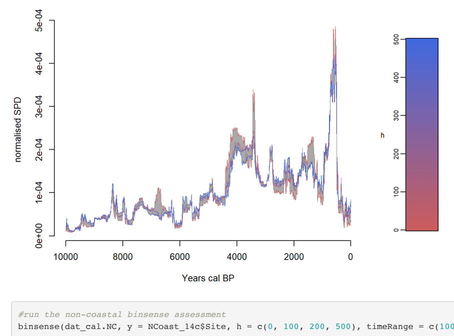


```
#run the non-coastal binsense assessment
binsense(dat_cal.NC, y = NCoast_14c$Site, h = c(0, 100, 200, 500), timeRange = c(10000, 0), verbose = FALSE)
```

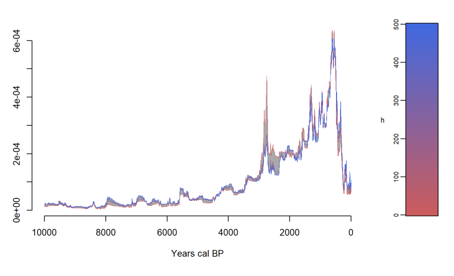

Supplemental Figures 8 & 9. Binsense evaluations of the impact of binning dates at between 0 and 500 years. Figure 8 shows the coastal binsense analysis while Figure 9 provides the non-coastal analysis. Based on the binsense evaluation, binning may produce an influential effect on our data as represented by the variation in relative spike size between a bin of 0 years and a bin of 500 years.

The purpose of binning is to account for ‘overdating’ of individual sites - the phenomenon where one site, or researcher(s), heavily dated an individual site leading to it distorting the relative population pattern of the past. This problem derives from the dates as data assumption that larger populations in the past will have produced more radiocarbon dateable material than in periods with smaller populations, leading to archaeologists finding more of the dateable material from periods with larger populations and, therefore, radiocarbon dating more material from periods with larger populations. If one site, or set of sites, is more heavily dated than the majority / average site, it may produce an artificially large population spike at one point in time that is not representative of the overall population at that point. Binning dates can account for this by limiting the use of dates from a site to one date from within a certain range (e.g., a bin of 200 means if there are 10 dates from one site that overlap in a 200 year window only one of those dates will be used for the spd). Binning, however, may then come to under represent variation in site size. For instance, a site with multiple sectors that has 20 dates likely should represent a larger population than a small hunter-gatherer camp site which has 20 dates capturing a highly detailed soil profile. Sans data on site size and dating density for all radiocarbon dates in the region, and With arguments plausible for both binning or not-binning, we elect to follow Riris (2018), Riris and Arroyo-Kalin (2019), and Roscoe et al (2021) as other scholars who have used 14C spd approaches in the Andes and generate bins of 200 years. This potentially limits the influence of well-dated large sites but, as the binsense plots show, the relative patterns across binning of 0 to 500 years remain the same and thus any bin-size is providing the same relative pattern representing past populations.

Further, following these past dates as data scholars working in the Central Andes we elect not to implement the Surovell et al. (2009) taphonomic loss equation [updated in Bluhm and Surovell 2019]. Surovell et al. (2009) argued that the dates as data assumption is limited by the potential loss of older landscapes that would have held older radiocarbon dateable material. The loss of older landscapes removing the material that could be dated would result in systematically lower relative population estimates than should exist earlier in time. The argument’s logic is sound and plausible, with recent work by Contreras and Codding (2024) demonstrating how a local, empirical, taphonomic correction can be built and applied with enough resources. Their work also highlighted that the uniform, volcanic, correction as proposed in Surovell et al. (2009) and Bluhm and Surovell (2019) does not, “just under-correct or over-correct, but may do one or the other for different spans of time, depending on local landscape taphonomy,” that in particular downweights the last 4000 years (Contreras and Codding 2024:16). As a result, they highlight that it may be more parsimonious to correct data only for earlier periods while leaving later period data uncorrected, though acknowledge there is no set manner in which to determine where the point at which correction should begin / stop resides, and suggest that projects working dominantly within the last 4000 years may be better off not applying a global correction. Their results, and that of prior work in the Central Andes (Wilson et al. 2022, Riris 2018; Riris and Arroyo-Kalin 2019; Roscoe et al. 2021), thus leaves it an open question as to whether taphonomic corrections should or should not be applied. While we do not have a local, empirical, correction for the Andes we do explore the impacts of applying or not applying a global taphonomic correction to our population proxy.

As an initial check, we visualize from when, in time, our samples derive.

```
par(pty = "s")
hist((indv.data$start.date.bp + indv.data$end.date.bp / 2), xlab = "Years BP", main = NA)
```

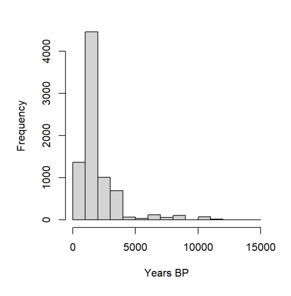

Supplemental Figure 10. Histogram of each individual’s mean date to visualize data distribution in time. Notably, the majority of the data in our dataset comes from within the past 4,000 years - after the point at which taphonomic correction is expected to be most influential / required. *Mean dates are only used for this visualization - they are not used in the analysis as the analysis relies upon iterative resampling of conditions from across each individual’s full potential date range.*

Even though the plurality of our data comes late in time, we still evaluate the application of taphonomic correction to the data. First, for creating the relative population reconstruction, we create 200 year bins for the kernel density estimates.

```
bins.C <- binPrep(Coast_14c$Site, dat_cal.C, h = 200, method = "complete")
bins.NC <- binPrep(NCoast_14c$Site, dat_cal.NC, h = 200, method = "complete")
```

We employ Kernel Density Estimates in the analysis to address concerns about incorporation of temporal uncertainty brought about by using Summed Probability Distributions. Therefore we will generate 1000 unique KDEs that we will subsample in Monte Carlo style for each site and individual following Wilson et al (2022) as prior work by Hinz (2020) has also found Monte Carlo method helps provide confidence envelopes that decrease false-positive results.

```
Coast.kde.sampledates <- sampleDates(dat_cal.C, bins = bins.C, nsim = 1000, boot = TRUE)
Coast.ckde <- ckde(Coast.kde.sampledates, timeRange = c(20000,0), bw = 50)


NCoast.kde.sampledates <- sampleDates(dat_cal.NC, bins = bins.NC, nsim = 1000, boot = TRUE)
NCoast.ckde <- ckde(NCoast.kde.sampledates, timeRange = c(20000,0), bw = 50)

#Save the ckde file so that we can read it in without having to rerun the code each time
save(Coast.ckde, file = "./data/Coast.ckde.1000.Rdata")
save(NCoast.ckde, file = "./data/NCoast.ckde.1000.Rdata")
```

We can visualize this data to observe reconstructed relative population patterns by coast and non-coast. Visualizing each of the 1000 KDEs shows the uncertainty in the reconstruction without applying taphonomic loss corrections.

```
#Load the ckde data
load("./data/Coast.ckde.1000.Rdata")
load("./data/NCoast.ckde.1000.Rdata")

par(mfrow = c(1,2))
plot(Coast.ckde, xlim = c(0, 10000), type = "multiline")
abline(v=3749, lty = 2, col = "grey50", xpd = FALSE)
abline(v=2849, lty = 2, col = "grey50", xpd = FALSE)
abline(v=2449, lty = 2, col = "grey50", xpd = FALSE)
abline(v=1350, lty = 2, col = "grey50", xpd = FALSE)
abline(v=950, lty = 2, col = "grey50", xpd = FALSE)
abline(v=480, lty = 2, col = "grey50", xpd = FALSE)
abline(v=418, lty = 2, col = "grey50", xpd = FALSE)
mtext("LH", side = 1, line = -1, at = 449, cex = 0.75)
mtext("LIP", side = 3, line = -1, at = 715, cex = 0.75)
mtext("MH", side = 1, line = -1, at = 1150, cex = 0.75)
mtext("EIP", side = 3, line = -1, at = 1899.5, cex = 0.75)
mtext("EH", side = 1, line = -1, at = 2649, cex = 0.75)
mtext("IP", side = 3, line = -1, at = 3299, cex = 0.75)
mtext("PC", side = 1, line = -1, at = 4250, cex = 0.75)
mtext("Coast", side = 3, line = -1, at = 6800, cex = 0.75)

plot(NCoast.ckde, xlim = c(0, 10000), type = "multiline")
abline(v=3749, lty = 2, col = "grey50", xpd = FALSE)
abline(v=2849, lty = 2, col = "grey50", xpd = FALSE)
abline(v=2449, lty = 2, col = "grey50", xpd = FALSE)
abline(v=1350, lty = 2, col = "grey50", xpd = FALSE)
abline(v=950, lty = 2, col = "grey50", xpd = FALSE)
abline(v=480, lty = 2, col = "grey50", xpd = FALSE)
abline(v=418, lty = 2, col = "grey50", xpd = FALSE)
mtext("LH", side = 1, line = -1, at = 449, cex = 0.75)
mtext("LIP", side = 3, line = -1, at = 715, cex = 0.75)
mtext("MH", side = 1, line = -1, at = 1150, cex = 0.75)
mtext("EIP", side = 3, line = -1, at = 1899.5, cex = 0.75)
mtext("EH", side = 1, line = -1, at = 2649, cex = 0.75)
mtext("IP", side = 3, line = -1, at = 3299, cex = 0.75)
mtext("PC", side = 1, line = -1, at = 4250, cex = 0.75)
mtext("Non-Coast", side = 3, line = -1, at = 6800, cex = 0.75)
```

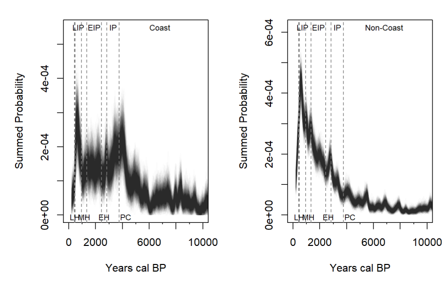

Supplemental Figure 11. Kernel Density Estimates of taphonomically uncorrected relative past population patterns for both the coast and non-coastal samples. The coastal pattern is suggestive of an early population spike around 4000 years calBP that plateaued until just pre-contact where another rapid population expansion occurred. The non-coastal data suggests exponential growth with acceleration beginning around 4000 years calBP and continuing up until roughly Spanish contact. While broad-scale paleodemographic reconstructions in the Central Andes are limited, the long-standing patterns in terms of site densities, population aggregations, and population in-filling, as well as local reconstructions of population patterns (e.g., Ikehara 2016), are in-line with these patterns.

We can also create and visualize what the population curves would look like with a taphonomic loss correction applied.

```
#Eq. 4 (combined function) from Bluhm and Surovell (2019)
correct <- function(x){(21149.57*(x+1788.03)^-1.26)} #generate the function to apply the correction

#calculate the expected proportion of sites lost per date:
lostpop <- correct(x = seq(20000,0,-1))

#apply the correction equation to each of the 1000 KDEs for each region
Coast.ckde.corr <- Coast.ckde
for(i in 1:1000){
  Coast.ckde.corr$res.matrix[,i] <- Coast.ckde.corr$res.matrix[,i] / lostpop
}

NCoast.ckde.corr <- NCoast.ckde
for(i in 1:1000){
  NCoast.ckde.corr$res.matrix[,i] <- NCoast.ckde.corr$res.matrix[,i] / lostpop
}
```

Visualize the corrected kdes.

```
par(mfrow = c(1,2))
plot(Coast.ckde.corr, xlim = c(0, 10000), type = "multiline")
abline(v=3749, lty = 2, col = "grey50", xpd = FALSE)
abline(v=2849, lty = 2, col = "grey50", xpd = FALSE)
abline(v=2449, lty = 2, col = "grey50", xpd = FALSE)
abline(v=1350, lty = 2, col = "grey50", xpd = FALSE)
abline(v=950, lty = 2, col = "grey50", xpd = FALSE)
abline(v=480, lty = 2, col = "grey50", xpd = FALSE)
abline(v=418, lty = 2, col = "grey50", xpd = FALSE)
mtext("LH", side = 1, line = -1, at = 449, cex = 0.75)
mtext("LIP", side = 3, line = -1, at = 715, cex = 0.75)
mtext("MH", side = 1, line = -1, at = 1150, cex = 0.75)
mtext("EIP", side = 3, line = -1, at = 1899.5, cex = 0.75)
mtext("EH", side = 1, line = -1, at = 2649, cex = 0.75)
mtext("IP", side = 3, line = -1, at = 3299, cex = 0.75)
mtext("PC", side = 1, line = -1, at = 4250, cex = 0.75)
mtext("Coast", side = 3, line = -1, at = 6800, cex = 0.75)

plot(NCoast.ckde.corr, xlim = c(0, 10000), type = "multiline")
abline(v=3749, lty = 2, col = "grey50", xpd = FALSE)
abline(v=2849, lty = 2, col = "grey50", xpd = FALSE)
abline(v=2449, lty = 2, col = "grey50", xpd = FALSE)
abline(v=1350, lty = 2, col = "grey50", xpd = FALSE)
abline(v=950, lty = 2, col = "grey50", xpd = FALSE)
abline(v=480, lty = 2, col = "grey50", xpd = FALSE)
abline(v=418, lty = 2, col = "grey50", xpd = FALSE)
mtext("LH", side = 1, line = -1, at = 449, cex = 0.75)
mtext("LIP", side = 3, line = -1, at = 715, cex = 0.75)
mtext("MH", side = 1, line = -1, at = 1150, cex = 0.75)
mtext("EIP", side = 3, line = -1, at = 1899.5, cex = 0.75)
mtext("EH", side = 1, line = -1, at = 2649, cex = 0.75)
mtext("IP", side = 3, line = -1, at = 3299, cex = 0.75)
mtext("PC", side = 1, line = -1, at = 4250, cex = 0.75)
mtext("Non-Coast", side = 3, line = -1, at = 6800, cex = 0.75)
```

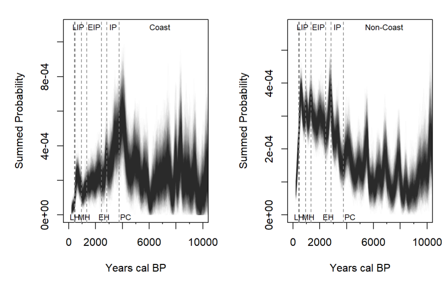

Supplemental Figure 12. Kernel Density Estimates of corrected relative past population patterns for both the coast and non-coastal samples.

Wilson et al. (2022:Figure 2) presented kernel density estimate versions of the dates as data relative past population size for Peru using both uncorrected and corrected, via volcanic correction from Surovell et al. (2009), approaches. These elicit some of the same concerns raised in other articles (e.g., Contreras and Codding 2024) regarding the effects of correction. When applying a global taphonomic correction for loss of landforms, the coastal pattern would suggest population around 10000 years ago was equal in size to just populations just before Spanish contact and that population apexed around 4000 calBP with a pattern of population decline over the subsequent nearly 4000 years. The non-coastal pattern would imply around 10000 years ago populations were roughly the same as during the Initial Period and Early Horizon, experiencing exponential growth beginning around 8000 calBP and broadly plateauing by around 3000 calBP. The drastic change in coastal and non-coastal population patterns with taphonomic correction is out of phase with the prevailing expectations of population patterns and stands in stark contrast to estimates of population patterns in the region (e.g., Billman 1996; Ikehara 2016) and to prevailing assumptions within the archaeological community based on the change in site frequency and site size through time indicative of larger population sizes later in time than earlier in the region. The taphonomically corrected coastal population spike at 4k yBP suggests the largest populations drastically pre-date the emergence of large, aggregated, sites and civilizations and seems to pre-date the full adoption of agriculture as a primary subsistence strategy. It seems unlikely that the largest populations in the region pre-date agriculture and the periods of sociopolitical complexity rise. The uncorrected curves more closely fit the expectations based upon other data for the area. Wilson et al. (2022) also conducted full climate - behavior analyses using the complete set of corrected and uncorrected kernel density estimates, finding no appreciable difference in the outcome or interpretation of analyses. This is likely due to the preponderance of data deriving from after 4k yBP in that study, the same as for this current study. Further, recent work by Codding et al. (2024) provides evidence that an uncorrected dates as data approach captures relative demographic patterns across the near past (less than 5k years ago) accurately. Finally, in the Central Andes, scholars have employed (or not) taphonomic correction based upon research question, with many synthetic studies (Riris 2018; Riris and Arroyo-Kalin 2019; Roscoe et al. 2021) determining taphonomic correction was less parsimonious for similar datasets to ours.

Given a) the majority of our data comes from the past 4,000 years when taphonomic correction effects are minimal and/or inappropriate to apply (see Contreras and Codding 2024), b) prior studies in the region finding minimal to no influence of using corrected or non-corrected data (Wilson et al. 2022; Gayo et al. 2015), and c) the fact the taphonomically corrected KDEs (Supplemental Figure 12) violate other paleodemographic reconstructions and expectations while the non-taphonomically corrected KDEs fall in-line with them, we elect to use uncorrected data as our population proxy.

To link relative past population size with each individual, we resample the KDE estimates 1k times for each individual to capture the range of potential relative population sizes an individual may have experienced during their lifetime. Doing so enables us to control for temporal uncertainty through incorporation of the uncertainty in the relative population size reconstructions into statistical modeling.

This will be a similar procedure to the resampling process we did above for climate.

```
cluster <- parallel::makeCluster(10, type = "PSOCK")
doParallel::registerDoParallel(cl = cluster)#setup the cluster of cores for use in parallel processing
foreach::getDoParRegistered()#TRUE means cluster is registered
foreach::getDoParWorkers()#Returned value should match # cores (20 in this case)

#make sure we have the individual data
indv_data <- read.csv("./data/indv_data.csv")

start_time <- Sys.time()#track when procedure begins

#Create a loop to extract individual values and iterate 10,000 times
indv.population <- foreach(i = 1:length(indv_data$RecordID), .combine = rbind, .packages = "data.table") %dopar%
{
  #each session needs to load the indv data
    indv_data <- read.csv("./data/indv_data.csv")
    #select the focal individual
    indv <- indv_data[i,]
    #make a blank dataframe to hold the individual's population reconstruction
    pop.result <- data.frame()

    #Function to load an Rdata file and assign it a new name so that we can load either CKDE as the necessary one for the individual
    loadRData <- function(fileName){
    #loads an RData file, and returns it
    load(fileName)
    get(ls()[ls() != "fileName"])
    }
  #select the appropriate ckde data (coast or non-coast)
    if (indv$Region == "Far S coast" |indv$Region == "C coast" |indv$Region == "N coast" |indv$Region == "S coast" |indv$Region == "Ecuador coast"){
      Andes.ckde <- loadRData("./data/Coast.ckde.1000.Rdata") } else {
      Andes.ckde <- loadRData("./data/NCoast.ckde.1000.Rdata")
      }

    ckde.df <- as.data.frame(Andes.ckde$res.matrix) #pull out the data by year

    #loop to get 1000 samples per individual
    for(j in 1:1000) { #repeat the steps in the loop 10000 times for each individual
    kde.sample <- ckde.df[sample(ncol(ckde.df[1:1000]), 1)] #randomly grab 1 KDE fit from the 1000 we generated
    kde.vals <- data.frame("pop" = kde.sample, "year" = seq(20000, 0, -1)) #make a dataframe of that single KDE
    colnames(kde.vals) <- c("pop", "year")#make sure the columns are named correctly
    rownames(kde.vals) <- c()#drop row names
    population <- subset(kde.vals, year >=indv$end.date.bp & year <=indv$start.date.bp, select = pop) #Subset the KDE for the individual's time window
    pop.val <- population[sample(nrow(population), 1),] #get one value which corresponds to one value from one year and add it to an output dataframe
    pop.result <- rbind(pop.result, pop.val)
    }

  #reset the column name for eas of use
  colnames(pop.result) <- "pop.KDE"

  return(pop.result)
  }

stopCluster(cluster)

#create an object for all the population data
indv.data.pop.resampled <- indv.population

#save the population data
save(indv.data.pop.resampled, file = "./data/indv.data.pop.resampled.Rdata")

#record when procedure ended
end_time <- Sys.time()
#check how long it took to run
end_time - start_time
```

Now we want to put the population density data per individual into the same dataset as the climate data per individual so that all data is in one location.

```
#load the climate data
indv.data.resampled <- read.fst(path = "./data/indv.data.full.fst")

#load the population sample
load("./data/indv.data.pop.resampled.Rdata")

#add the population estimate to the climate estimate df
indv.data.resampled <- cbind(indv.data.resampled, indv.data.pop.resampled)

indv.data.resampled$Run <- rep(seq(1:1000), 7992)#set a Run number value of 1 to 1k for each site's 1k resamples

#order this so each individual's 1st sample is next to each other, their 2nd sample next to each other, etc. This means the first 7992 rows of the dataset are all unique individuals as is the second set of 7992 and so on until we have all 1000 replicates.
indv.data.resampled <- indv.data.resampled[order(indv.data.resampled$Run),]

#write out the full dataset as an fst file for use later
write.fst(indv.clim.data, "./data/indv.data.full.fst")

#could also do this with feather via arrow package
#write_feather(indv.clim.data, file = "./data/indv.data.resampled.feather", compression = "zstd")
```

And with that we have all of the data that we need to conduct our analyses.

Analysis

Step 1. Checking the Data

Now that we have all the data, we can begin some initial evaluations. Let’s start with checking how correlated climate variables are at the 3 potential distance measures for individuals.

First, read in the data

```
#Load the individual data (1000 replicates for each individual)
indv.data.corr <- read.fst("./data/indv.data.full.fst",
                  column = c("MNtemp5km", "MNtemp20km", "MNtemp50km", "SDtemp5km", "SDtemp20km", "SDtemp50km", "MNprcp5km", "MNprcp20km", "MNprcp50km", "SDprcp5km", "SDprcp20km", "SDprcp50km", "pop.KDE", "SST", "ENSO", "Individuals_state", "Region_Fact"))

clim.vars<-with(indv.data.corr,
           data.frame(
                MNtemp5km,
                MNtemp20km,
                MNtemp50km,
                SDtemp5km,
                SDtemp20km,
                SDtemp50km,
                MNprcp5km,
                MNprcp20km,
                MNprcp50km,
                SDprcp5km,
                SDprcp20km,
                SDprcp50km
           )
         )
```

Then, perform a visual check for variable normality to determine the appropriate test for variable correlation checks. We begin with temperature.

```
#Visual check for normality (sample size so large as to make shapiro-wilk test unreliable)
par(mfrow = c(1,3))
hist(clim.vars$MNtemp5km, xlab = "MN Temp 5km", main = NA)
hist(clim.vars$MNtemp20km, xlab = "MN Temp 20km", main = NA)
hist(clim.vars$MNtemp50km, xlab = "MN Temp 50km", main = NA)
```

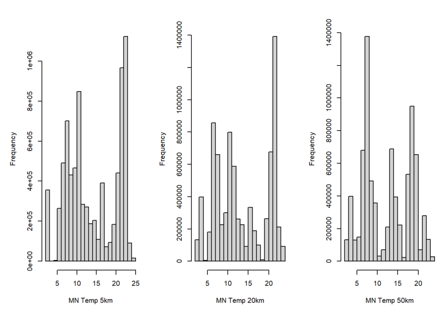

Supplemental Figure 13. Histograms of temperature data at each of the sample radii. Data are not normally distributed.

Now check temperature standard deviation.

```
par(mfrow = c(1,3))
hist(clim.vars$SDtemp5km, xlab = "SD Temp 5km", main = NA)
hist(clim.vars$SDtemp20km, xlab = "SD Temp 20km", main = NA)
hist(clim.vars$SDtemp50km, xlab = "SD Temp 50km", main = NA)
```

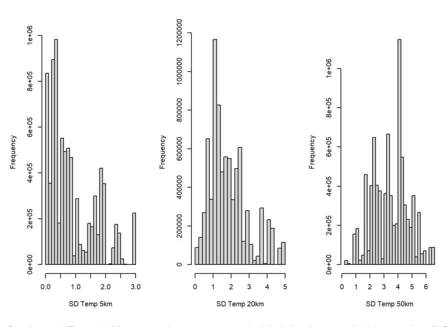

Supplemental Figure 14. Histograms of temperature standard deviation data at each of the sample radii. Data are not normally distributed.

Now check precipitation.

```
par(mfrow = c(1,3))
hist(clim.vars$MNprcp5km, xlab = "MN Prcp 5km", main = NA)
hist(clim.vars$MNprcp20km, xlab = "MN Prcp 20km", main = NA)
hist(clim.vars$MNprcp50km, xlab = "MN Prcp 50km", main = NA)
```

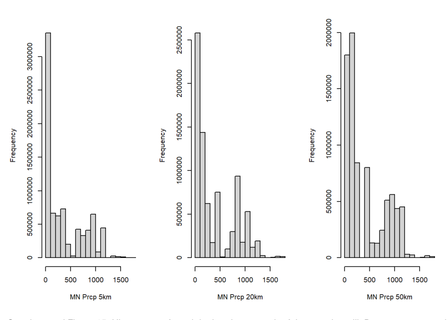

Supplemental Figure 15. Histograms of precipitation data at each of the sample radii. Data are not normally distributed.

Finally, check precipitation standard deviation

```
par(mfrow = c(1,3))
hist(clim.vars$SDprcp5km, xlab = "SD Prcp 5km", main = NA)
hist(clim.vars$SDprcp20km, xlab = "SD Prcp 20km", main = NA)
hist(clim.vars$SDprcp50km, xlab = "SD Prcp 50km", main = NA)
```

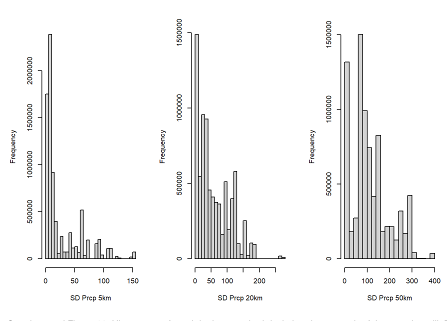

Supplemental Figure 16. Histograms of precipitation standard deviation data at each of the sample radii. Data are not normally distributed.

It is clear that our data are not normally distributed. This is unsurprising as they are sampled at individuals’ locations and the individuals are not perfectly randomly distributed across the Central Andes. As a result, to compare correlations between variables we will make use of the Spearman’s Rho correlation statistic. Here we use Spearman’s Rho as the computational time for Kendall’s Tau, given our ~8,000,000 rows of data, is prohibitive. Spearman’s Rho, as a non-parametric test of correlation, is more appropriate for our non-normal data.

```
#Create a data.frame of just the climate variables.
clim.vars<-with(indv.data.corr,
             data.frame(
                    MNtemp5km,
                    MNtemp20km,
                    MNtemp50km,
                    SDtemp5km,
                    SDtemp20km,
                    SDtemp50km,
                    MNprcp5km,
                    MNprcp20km,
                    MNprcp50km,
                    SDprcp5km,
                    SDprcp20km,
                    SDprcp50km
             )
           )

#then test collinearity
cm<- cor(clim.vars,
         method="spearman",
         use="complete.obs")


# matrix of the p-value of the correlation
p.mat<-cor.mtest(clim.vars)
#plot the correlation matrix
corrplot(cm,
      method="color",
      type="upper",
      col = viridis(n=20),
      diag=FALSE,
      bg="grey90",
      addCoef.col = "black",
      tl.col="black",
      outline = FALSE,
      addCoefasPercent=FALSE,
      number.cex = 0.75,
      p.mat = p.mat$p, sig.level = 0.05, insig = "blank"
  )
```

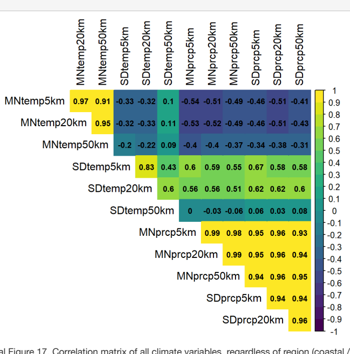

Supplemental Figure 17. Correlation matrix of all climate variables, regardless of region (coastal / non-coastal). The key takeaways are: 1) the intra-climate variable correlations are very strong (i.e., mean temp at 5km is tightly correlated with the mean temp at 20 and 50km) and hold for most of the climate variables. Therefore we can use any of the distance measures to capture the same local climatic pattern for an individual. 2) Mean precipitation and standard deviation precipitation are very tightly correlated. We therefore don't want to use both in a single model. In our models we will rely just on mean precipitation. This results in the fact that mean precipitation proxies both productivity and heterogeneity in productivity (when there is more precip it is more spatially variable).

For our analysis we use the 20km buffer as 20km roughly translates to a day's walk or a catchment for individuals around their site. We also check the correlation of the 20km buffered climate variables with SST and ENSO.

```
clim.vars<-with(indv.data.corr,
             data.frame(
                    MNtemp20km,
                    SDtemp20km,
                    MNprcp20km,
                    SDprcp20km,
                    pop.KDE,
                    SST,
                    ENSO
             )
           )

#then test collinearity
cm<- cor(clim.vars,
         method="spearman",
         use="complete.obs")


# matrix of the p-value of the correlation
p.mat<-cor.mtest(clim.vars)
#plot the correlation matrix
corrplot(cm,
      method="color",
      type="upper",
      col = viridis(n=20),
      diag=FALSE,
      bg="grey90",
      addCoef.col = "black",
      tl.col="black",
      outline = FALSE,
      addCoefasPercent=FALSE,
      p.mat = p.mat$p, sig.level = 0.05, insig = "blank"
  )
```

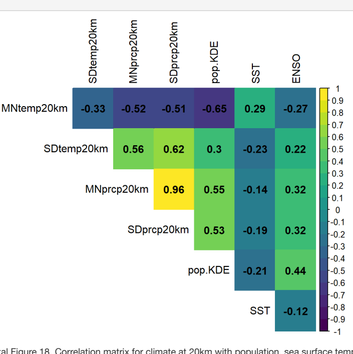

Supplemental Figure 18. Correlation matrix for climate at 20km with population, sea surface temperature, and ENSO. There remains some autocorrelation between some of the variables however only one is greater than a potentially problematic +- 0.7 (Dormann et al. 2013): mean and standard deviation in precipitation. Thus, RF analyses can readily handle incorporation of each variable except these two in the analysis.

We will do one final check on variable autocorrelation, looking at the sample subsets for coastal and inland individuals as the RF analyses for each region are run separately. This way we ensure neither region has a problematic variable autocorrelation.

```
clim.vars.C<-with(indv.data.corr,
             data.frame(
                    MNtemp20km,
                    SDtemp20km,
                    MNprcp20km,
                    SDprcp20km,
                    pop.KDE,
                    SST,
                    ENSO,
                    Region_Fact
             )
           )

clim.vars.NC<-with(indv.data.corr,
             data.frame(
                    MNtemp20km,
                    SDtemp20km,
                    MNprcp20km,
                    SDprcp20km,
                    pop.KDE,
                    ENSO,
                    Region_Fact
             )
           )

clim.vars.C <- clim.vars.C[which(clim.vars.C$Region_Fact == "coastal"),]
clim.vars.C <- clim.vars.C[,-8]#drop the region variable
clim.vars.NC <- clim.vars.NC[which(clim.vars.NC$Region_Fact == "non-coastal"),]
clim.vars.NC <- clim.vars.NC[,-7]#drop the region variable

#then test collinearity
cm.C<- cor(clim.vars.C,
         method="spearman",
         use="complete.obs")

cm.NC<- cor(clim.vars.NC,
         method="spearman",
         use="complete.obs")

# matrix of the p-value of the correlation
p.mat.C<-cor.mtest(clim.vars.C)

p.mat.NC<-cor.mtest(clim.vars.NC)

#plot the correlation matrix
par(mfrow = c(1,2))
#png(filename = "Coastal_Corrplot.png", width = 4, height = 4, units = "in", bg = "NA", res = 300)
#pdf(file = "Coastal_Corrplot.pdf", width = 4, height = 4, bg = "NA")
corrplot(cm.C,
      method="color",
      type="upper",
      col = viridis(n=20),
      diag=FALSE,
      bg="grey90",
      addCoef.col = "black",
      tl.col="black",
      tl.cex = 1,
      cl.cex = 1,
      cl.length = 3,
      outline = FALSE,
      addCoefasPercent=FALSE,
      p.mat = p.mat.C$p, sig.level = 0.05, insig = "blank"
  )
#dev.off()
#png(filename = "Inland_Corrplot.png", width = 4, height = 4, bg = "NA", units = "in", res = 300)
#pdf(file = "Inland_Corrplot.pdf", width = 4, height = 4, bg = "NA")
corrplot(cm.NC,
      method="color",
      type="upper",
      col = viridis(n=20),
      diag=FALSE,
      bg="grey90",
      addCoef.col = "black",
      tl.col="black",
      tl.cex = 1,
      cl.cex = 1,
      cl.length = 3,
      outline = FALSE,
      addCoefasPercent=FALSE,
      p.mat = p.mat.NC$p, sig.level = 0.05, insig = "blank"
  )
```

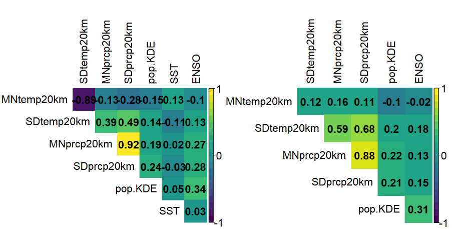

Supplemental Figure 19. Correlation matrix for coastal (left) and non-coastal (right) independent variables. On the coasts (left panel) mean temperature and standard deviation in temperature are tightly correlated (inversely). As it gets warmer on the coast the temperature gets less variable. We will drop the standard deviation in temperature from our analysis to prioritize mean temperature measure, acknowledging that low temperatures correspond with greater volatility while higher temperatures represent greater stability. We favor keeping mean temperature over standard deviation as, on the coasts, based on the ecology, temperature has a greater impact and warmer temperatures that are also less variable would be expected, based on theory, to have the same impact on peace (promote it) whereas colder temperatures that are also more variable would be expected to incentivize conflict. In both regions mean precipitation and standard deviation in precipitation remain very tightly, positively, correlated. Increasing precipitation equates to greater precipitation variability (e.g., volatility) and we therefore drop standard deviation in precipitation from the analyses. All other variables are under a correlation strength of +-0.7 and can be readily handled by an RF analysis.

Mean and standard deviation in temperature have different value ranges but appear tightly correlated. To map this out, we visualize how tightly the two variables are correlated below.

```
par(pty = "s", mfrow = c(1,2))
plot(clim.vars.C$SDprcp20km[1:7992] ~ clim.vars.C$MNprcp20km[1:7992], xlab = "Mean Prcp 20km", ylab = "SD Prcp 20
km", main = "Coastal")
plot(clim.vars.NC$SDprcp20km[1:7992] ~ clim.vars.NC$MNprcp20km[1:7992], xlab = "Mean Prcp 20km", ylab = "SD Prcp
20km", main = "Inland")
```

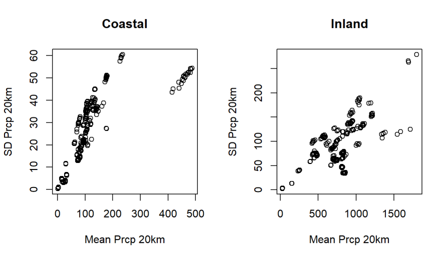

Supplemental Figure 20. Scatterplots of mean and standard deviation in temperature for the coasts (left) and non-coasts (right). Mean and standard deviation in precipitation are very tightly correlated. Based on the correlation test results and the visual assessment, it is inappropriate to include both mean and standard deviation in precipitation in our analytical models as they are effectively measuring the same thing and we therefore drop standard deviation, acknowledging its effect is captured by patterning in mean precipitation.

Step 2. Prep Model Analysis

With the data assembled and checked, we can now procede to analyse our predictions.

First, we get the min and max values for predictor variables as we will need those for getting partial response plots.

```
#lets get min and max data for our variables
variable <- read.fst("./data/indv.data.full.fst", column = c("MNtemp20km", "Region_Fact")) #MNT
minMNT.C <- min(variable$MNtemp20km[which(variable$Region_Fact == "coastal")])
medMNT.C <- median(variable$MNtemp20km[which(variable$Region_Fact == "coastal")])
maxMNT.C <- max(variable$MNtemp20km[which(variable$Region_Fact == "coastal")])

minMNT.NC <- min(variable$MNtemp20km[which(variable$Region_Fact == "non-coastal")])
medMNT.NC <- median(variable$MNtemp20km[which(variable$Region_Fact == "non-coastal")])
maxMNT.NC <- max(variable$MNtemp20km[which(variable$Region_Fact == "non-coastal")])


variable <- read.fst("./data/indv.data.full.fst", column = c("SDtemp20km", "Region_Fact")) #SDT
minSDT.NC <- min(variable$SDtemp20km[which(variable$Region_Fact == "non-coastal")])
medSDT.NC <- median(variable$SDtemp20km[which(variable$Region_Fact == "non-coastal")])
maxSDT.NC <- max(variable$SDtemp20km[which(variable$Region_Fact == "non-coastal")])


variable <- read.fst("./data/indv.data.full.fst", column = c("MNprcp20km", "Region_Fact")) #MNP
minMNP.C <- min(variable$MNprcp20km[which(variable$Region_Fact == "coastal")])
medMNP.C <- median(variable$MNprcp20km[which(variable$Region_Fact == "coastal")])
maxMNP.C <- max(variable$MNprcp20km[which(variable$Region_Fact == "coastal")])

minMNP.NC <- min(variable$MNprcp20km[which(variable$Region_Fact == "non-coastal")])
medMNP.NC <- median(variable$MNprcp20km[which(variable$Region_Fact == "non-coastal")])
maxMNP.NC <- max(variable$MNprcp20km[which(variable$Region_Fact == "non-coastal")])

variable <- read.fst("./data/indv.data.full.fst", column = c("pop.KDE", "Region_Fact")) #POP
minPOP.C <- min(variable$pop.KDE[which(variable$Region_Fact == "coastal")])
medPOP.C <- median(variable$pop.KDE[which(variable$Region_Fact == "coastal")])
maxPOP.C <- max(variable$pop.KDE[which(variable$Region_Fact == "coastal")])

minPOP.NC <- min(variable$pop.KDE[which(variable$Region_Fact == "non-coastal")])
medPOP.NC <- median(variable$pop.KDE[which(variable$Region_Fact == "non-coastal")])
maxPOP.NC <- max(variable$pop.KDE[which(variable$Region_Fact == "non-coastal")])

variable <- read.fst("./data/indv.data.full.fst", column = c("SST", "Region_Fact")) #CVP
minSST.C <- min(variable$SST[which(variable$Region_Fact == "coastal")])
medSST.C <- median(variable$SST[which(variable$Region_Fact == "coastal")])
maxSST.C <- max(variable$SST[which(variable$Region_Fact == "coastal")])

variable <- read.fst("./data/indv.data.full.fst", column = c("ENSO", "Region_Fact")) #CVP
minENS.C <- min(variable$ENSO[which(variable$Region_Fact == "coastal")])
medENS.C <- median(variable$ENSO[which(variable$Region_Fact == "coastal")])
maxENS.C <- max(variable$ENSO[which(variable$Region_Fact == "coastal")])

minENS.NC <- min(variable$ENSO[which(variable$Region_Fact == "non-coastal")])
medENS.NC <- median(variable$ENSO[which(variable$Region_Fact == "non-coastal")])
maxENS.NC <- max(variable$ENSO[which(variable$Region_Fact == "non-coastal")])

rm(variable) #don't hang on to 79 million rows of data - just keep the min/max values we need for predicted parti
al grids
```

Our hypotheses will be evaluated with the partial response plots of trauma (Y/N) to variation in the independent variables. To do get predicted partial responses we need to have a grid of values for each independent variable over which we will conduct predictions. We also want to do multi-dimensional partial response plots to look at interactions that better track volatility combined with population pressure, so we generate additional grids so that each model predicts over the same range of multiple variables for better comparison.

```
#Make the grids for 2d partial response (so we can get the influence of each variables while all other variables
are held at their average effect)
PDP.MNT.C <- data.frame(MNtemp20km = seq(minMNT.C, maxMNT.C, length.out = 21))
PDP.MNT.NC <- data.frame(MNtemp20km = seq(minMNT.NC, maxMNT.NC, length.out = 21))

PDP.SDT.NC <- data.frame(SDtemp20km = seq(minSDT.NC, maxSDT.NC, length.out = 21))

PDP.MNP.C <- data.frame(MNprcp20km = seq(minMNP.C, maxMNP.C, length.out = 21))
PDP.MNP.NC <- data.frame(MNprcp20km = seq(minMNP.NC, maxMNP.NC, length.out = 21))

PDP.POP.C <- data.frame(pop.KDE = seq(minPOP.C, maxPOP.C, length.out = 21))
PDP.POP.NC <- data.frame(pop.KDE = seq(minPOP.NC, maxPOP.NC, length.out = 21))

PDP.SST.C <- data.frame(SST = seq(minSST.C, maxSST.C, length.out = 21))

PDP.ENS.C <- data.frame(ENSO = seq(minENS.C, maxENS.C, length.out = 21))
PDP.ENS.NC <- data.frame(ENSO = seq(minENS.NC, maxENS.NC, length.out = 21))

PDP.STT.C <- data.frame(Individuals_state = c(rep(0, times = 7), rep(1, times = 7), rep(2, times = 7)))
PDP.STT.NC <- data.frame(Individuals_state = c(rep(0, times = 7), rep(1, times = 7), rep(2, times = 7)))

#For the 3d partial responses, let's begin by making the grid.out value. We are interested in the interaction of
climate variables with population density and the terrestrial climate mean variables with each other as these int
eractions characterize climate volatility vs stability and interactions with population. SDT, ENSO, SST already c
apture aspects of volatility.
#So we need a value set for population.
pop.KDE.gridout.C <- seq(minPOP.C, maxPOP.C, length.out = 20)
pop.KDE.gridout.NC <- seq(minPOP.NC, maxPOP.NC, length.out = 20)

#Now make the predictive grids

PDP.MNT.POP.C <- data.frame(MNtemp20km = rep(seq(minMNT.C, maxMNT.C, length.out = 20), times = 20), pop.KDE = c(r
ep(pop.KDE.gridout.C[1], times = 20), rep(pop.KDE.gridout.C[2], times = 20), rep(pop.KDE.gridout.C[3], times = 2
0), rep(pop.KDE.gridout.C[4], times = 20), rep(pop.KDE.gridout.C[5], times = 20), rep(pop.KDE.gridout.C[6], times
= 20), rep(pop.KDE.gridout.C[7], times = 20), rep(pop.KDE.gridout.C[8], times = 20), rep(pop.KDE.gridout.C[9], ti
mes = 20), rep(pop.KDE.gridout.C[10], times = 20), rep(pop.KDE.gridout.C[11], times = 20), rep(pop.KDE.gridout.C[
12], times = 20), rep(pop.KDE.gridout.C[13], times = 20), rep(pop.KDE.gridout.C[14], times = 20), rep(pop.KDE.gri
dout.C[15], times = 20), rep(pop.KDE.gridout.C[16], times = 20), rep(pop.KDE.gridout.C[17], times = 20), rep(pop.
KDE.gridout.C[18], times = 20), rep(pop.KDE.gridout.C[19], times = 20), rep(pop.KDE.gridout.C[20], times = 20)
))
PDP.MNT.POP.NC <- data.frame(MNtemp20km = rep(seq(minMNT.NC, maxMNT.NC, length.out = 20), times = 20), pop.KDE =
c(rep(pop.KDE.gridout.NC[1], times = 20), rep(pop.KDE.gridout.NC[2], times = 20), rep(pop.KDE.gridout.NC[3], time
s = 20), rep(pop.KDE.gridout.NC[4], times = 20), rep(pop.KDE.gridout.NC[5], times = 20), rep(pop.KDE.gridout.NC[
6], times = 20), rep(pop.KDE.gridout.NC[7], times = 20), rep(pop.KDE.gridout.NC[8], times = 20), rep(pop.KDE.grid
out.NC[9], times = 20), rep(pop.KDE.gridout.NC[10], times = 20), rep(pop.KDE.gridout.NC[11], times = 20), rep(po
p.KDE.gridout.NC[12], times = 20), rep(pop.KDE.gridout.NC[13], times = 20), rep(pop.KDE.gridout.NC[14], times = 2
0), rep(pop.KDE.gridout.NC[15], times = 20), rep(pop.KDE.gridout.NC[16], times = 20), rep(pop.KDE.gridout.NC[17],
times = 20), rep(pop.KDE.gridout.NC[18], times = 20), rep(pop.KDE.gridout.NC[19], times = 20), rep(pop.KDE.gridou
t.NC[20], times = 20)
))

PDP.SDT.POP.NC <- data.frame(SDtemp20km = rep(seq(minSDT.NC, maxSDT.NC, length.out = 20), times = 20), pop.KDE =
c(rep(pop.KDE.gridout.C[1], times = 20), rep(pop.KDE.gridout.C[2], times = 20), rep(pop.KDE.gridout.C[3], times =
20), rep(pop.KDE.gridout.C[4], times = 20), rep(pop.KDE.gridout.C[5], times = 20), rep(pop.KDE.gridout.C[6], time
s = 20), rep(pop.KDE.gridout.C[7], times = 20), rep(pop.KDE.gridout.C[8], times = 20), rep(pop.KDE.gridout.C[9],
times = 20), rep(pop.KDE.gridout.C[10], times = 20), rep(pop.KDE.gridout.C[11], times = 20), rep(pop.KDE.gridout.
C[12], times = 20), rep(pop.KDE.gridout.C[13], times = 20), rep(pop.KDE.gridout.C[14], times = 20), rep(pop.KDE.g
ridout.C[15], times = 20), rep(pop.KDE.gridout.C[16], times = 20), rep(pop.KDE.gridout.C[17], times = 20), rep(po
p.KDE.gridout.C[18], times = 20), rep(pop.KDE.gridout.C[19], times = 20), rep(pop.KDE.gridout.C[20], times = 20)
))

PDP.MNP.POP.C <- data.frame(MNprcp20km = rep(seq(minMNP.C, maxMNP.C, length.out = 20), times = 20), pop.KDE = c(r
ep(pop.KDE.gridout.C[1], times = 20), rep(pop.KDE.gridout.C[2], times = 20), rep(pop.KDE.gridout.C[3], times = 2
0), rep(pop.KDE.gridout.C[4], times = 20), rep(pop.KDE.gridout.C[5], times = 20), rep(pop.KDE.gridout.C[6], times
= 20), rep(pop.KDE.gridout.C[7], times = 20), rep(pop.KDE.gridout.C[8], times = 20), rep(pop.KDE.gridout.C[9], ti
mes = 20), rep(pop.KDE.gridout.C[10], times = 20), rep(pop.KDE.gridout.C[11], times = 20), rep(pop.KDE.gridout.C[
12], times = 20), rep(pop.KDE.gridout.C[13], times = 20), rep(pop.KDE.gridout.C[14], times = 20), rep(pop.KDE.gri
dout.C[15], times = 20), rep(pop.KDE.gridout.C[16], times = 20), rep(pop.KDE.gridout.C[17], times = 20), rep(pop.
KDE.gridout.C[18], times = 20), rep(pop.KDE.gridout.C[19], times = 20), rep(pop.KDE.gridout.C[20], times = 20)
))
PDP.MNP.POP.NC <- data.frame(MNprcp20km = rep(seq(minMNP.NC, maxMNP.NC, length.out = 20), times = 20), pop.KDE =
c(rep(pop.KDE.gridout.NC[1], times = 20), rep(pop.KDE.gridout.NC[2], times = 20), rep(pop.KDE.gridout.NC[3], time
s = 20), rep(pop.KDE.gridout.NC[4], times = 20), rep(pop.KDE.gridout.NC[5], times = 20), rep(pop.KDE.gridout.NC[
6], times = 20), rep(pop.KDE.gridout.NC[7], times = 20), rep(pop.KDE.gridout.NC[8], times = 20), rep(pop.KDE.grid
out.NC[9], times = 20), rep(pop.KDE.gridout.NC[10], times = 20), rep(pop.KDE.gridout.NC[11], times = 20), rep(po
p.KDE.gridout.NC[12], times = 20), rep(pop.KDE.gridout.NC[13], times = 20), rep(pop.KDE.gridout.NC[14], times = 2
0), rep(pop.KDE.gridout.NC[15], times = 20), rep(pop.KDE.gridout.NC[16], times = 20), rep(pop.KDE.gridout.NC[17],
times = 20), rep(pop.KDE.gridout.NC[18], times = 20), rep(pop.KDE.gridout.NC[19], times = 20), rep(pop.KDE.gridou
t.NC[20], times = 20)
))

PDP.SST.POP.C <- data.frame(SST = rep(seq(minSST.C, maxSST.C, length.out = 20), times = 20), pop.KDE = c(rep(pop.
KDE.gridout.C[1], times = 20), rep(pop.KDE.gridout.C[2], times = 20), rep(pop.KDE.gridout.C[3], times = 20), rep(
pop.KDE.gridout.C[4], times = 20), rep(pop.KDE.gridout.C[5], times = 20), rep(pop.KDE.gridout.C[6], times = 20),
rep(pop.KDE.gridout.C[7], times = 20), rep(pop.KDE.gridout.C[8], times = 20), rep(pop.KDE.gridout.C[9], times = 2
0), rep(pop.KDE.gridout.C[10], times = 20), rep(pop.KDE.gridout.C[11], times = 20), rep(pop.KDE.gridout.C[12], ti
mes = 20), rep(pop.KDE.gridout.C[13], times = 20), rep(pop.KDE.gridout.C[14], times = 20), rep(pop.KDE.gridout.C[
15], times = 20), rep(pop.KDE.gridout.C[16], times = 20), rep(pop.KDE.gridout.C[17], times = 20), rep(pop.KDE.gri
dout.C[18], times = 20), rep(pop.KDE.gridout.C[19], times = 20), rep(pop.KDE.gridout.C[20], times = 20)
))

PDP.ENS.POP.C <- data.frame(ENSO = rep(seq(minENS.C, maxENS.C, length.out = 20), times = 20), pop.KDE = c(rep(po
p.KDE.gridout.C[1], times = 20), rep(pop.KDE.gridout.C[2], times = 20), rep(pop.KDE.gridout.C[3], times = 20), re
p(pop.KDE.gridout.C[4], times = 20), rep(pop.KDE.gridout.C[5], times = 20), rep(pop.KDE.gridout.C[6], times = 2
0), rep(pop.KDE.gridout.C[7], times = 20), rep(pop.KDE.gridout.C[8], times = 20), rep(pop.KDE.gridout.C[9], times
= 20), rep(pop.KDE.gridout.C[10], times = 20), rep(pop.KDE.gridout.C[11], times = 20), rep(pop.KDE.gridout.C[12],
times = 20), rep(pop.KDE.gridout.C[13], times = 20), rep(pop.KDE.gridout.C[14], times = 20), rep(pop.KDE.gridout.
C[15], times = 20), rep(pop.KDE.gridout.C[16], times = 20), rep(pop.KDE.gridout.C[17], times = 20), rep(pop.KDE.g
ridout.C[18], times = 20), rep(pop.KDE.gridout.C[19], times = 20), rep(pop.KDE.gridout.C[20], times = 20)
))
PDP.ENS.POP.NC <- data.frame(ENSO = rep(seq(minENS.NC, maxENS.NC, length.out = 20), times = 20), pop.KDE = c(rep(
pop.KDE.gridout.NC[1], times = 20), rep(pop.KDE.gridout.NC[2], times = 20), rep(pop.KDE.gridout.NC[3], times = 2
0), rep(pop.KDE.gridout.NC[4], times = 20), rep(pop.KDE.gridout.NC[5], times = 20), rep(pop.KDE.gridout.NC[6], ti
mes = 20), rep(pop.KDE.gridout.NC[7], times = 20), rep(pop.KDE.gridout.NC[8], times = 20), rep(pop.KDE.gridout.N
C[9], times = 20), rep(pop.KDE.gridout.NC[10], times = 20), rep(pop.KDE.gridout.NC[11], times = 20), rep(pop.KDE.
gridout.NC[12], times = 20), rep(pop.KDE.gridout.NC[13], times = 20), rep(pop.KDE.gridout.NC[14], times = 20), re
p(pop.KDE.gridout.NC[15], times = 20), rep(pop.KDE.gridout.NC[16], times = 20), rep(pop.KDE.gridout.NC[17], times
= 20), rep(pop.KDE.gridout.NC[18], times = 20), rep(pop.KDE.gridout.NC[19], times = 20), rep(pop.KDE.gridout.NC[2
0], times = 20)
))

#We also need a precip grid
MNprcp.gridout.C <- seq(minMNP.C, maxMNP.C, length.out = 20)
MNprcp.gridout.NC <- seq(minMNP.NC, maxMNP.NC, length.out = 20)

PDP.MNT.MNP.C <- data.frame(MNtemp20km = rep(seq(minMNT.C, maxMNT.C, length.out = 20), times = 20), MNprcp20km =
c(rep(MNprcp.gridout.C[1], times = 20), rep(MNprcp.gridout.C[2], times = 20), rep(MNprcp.gridout.C[3], times = 2
0), rep(MNprcp.gridout.C[4], times = 20), rep(MNprcp.gridout.C[5], times = 20), rep(MNprcp.gridout.C[6], times =
20), rep(MNprcp.gridout.C[7], times = 20), rep(MNprcp.gridout.C[8], times = 20), rep(MNprcp.gridout.C[9], times =
20), rep(MNprcp.gridout.C[10], times = 20), rep(MNprcp.gridout.C[11], times = 20), rep(MNprcp.gridout.C[12], time
s = 20), rep(MNprcp.gridout.C[13], times = 20), rep(MNprcp.gridout.C[14], times = 20), rep(MNprcp.gridout.C[15],
times = 20), rep(MNprcp.gridout.C[16], times = 20), rep(MNprcp.gridout.C[17], times = 20), rep(MNprcp.gridout.C[1
8], times = 20), rep(MNprcp.gridout.C[19], times = 20), rep(MNprcp.gridout.C[20], times = 20)
))
PDP.MNT.MNP.NC <- data.frame(MNtemp20km = rep(seq(minMNT.NC, maxMNT.NC, length.out = 20), times = 20), MNprcp20km
= c(rep(MNprcp.gridout.NC[1], times = 20), rep(MNprcp.gridout.NC[2], times = 20), rep(MNprcp.gridout.NC[3], times
= 20), rep(MNprcp.gridout.NC[4], times = 20), rep(MNprcp.gridout.NC[5], times = 20), rep(MNprcp.gridout.NC[6], ti
mes = 20), rep(MNprcp.gridout.NC[7], times = 20), rep(MNprcp.gridout.NC[8], times = 20), rep(MNprcp.gridout.NC[
9], times = 20), rep(MNprcp.gridout.NC[10], times = 20), rep(MNprcp.gridout.NC[11], times = 20), rep(MNprcp.grido
ut.NC[12], times = 20), rep(MNprcp.gridout.NC[13], times = 20), rep(MNprcp.gridout.NC[14], times = 20), rep(MNprc
p.gridout.NC[15], times = 20), rep(MNprcp.gridout.NC[16], times = 20), rep(MNprcp.gridout.NC[17], times = 20), re
p(MNprcp.gridout.NC[18], times = 20), rep(MNprcp.gridout.NC[19], times = 20), rep(MNprcp.gridout.NC[20], times =
20)
))

#Now make the predictive grids for sociopolitical complexity
PDP.MNT.SPC.C <- data.frame(MNtemp20km = rep(seq(minMNT.C, maxMNT.C, length.out = 20), times = 3), Individuals_st
ate = c(rep(0, times = 20), rep(1, times = 20), rep(2, times = 20)
))
PDP.MNT.SPC.C$Individuals_state <- as.factor(PDP.MNT.SPC.C$Individuals_state)

PDP.MNT.SPC.NC <- data.frame(MNtemp20km = rep(seq(minMNT.NC, maxMNT.NC, length.out = 20), times = 3), Individuals
_state = c(rep(0, times = 20), rep(1, times = 20), rep(2, times = 20)
))
PDP.MNT.SPC.NC$Individuals_state <- as.factor(PDP.MNT.SPC.NC$Individuals_state)

PDP.SDT.SPC.NC <- data.frame(SDtemp20km = rep(seq(minSDT.NC, maxSDT.NC, length.out = 20), times = 3), Individuals
_state = c(rep(0, times = 20), rep(1, times = 20), rep(2, times = 20)
))
PDP.SDT.SPC.NC$Individuals_state <- as.factor(PDP.SDT.SPC.NC$Individuals_state)

PDP.MNP.SPC.C <- data.frame(MNprcp20km = rep(seq(minMNP.C, maxMNP.C, length.out = 20), times = 3), Individuals_st
ate = c(rep(0, times = 20), rep(1, times = 20), rep(2, times = 20)
))
PDP.MNP.SPC.C$Individuals_state <- as.factor(PDP.MNP.SPC.C$Individuals_state)

PDP.MNP.SPC.NC <- data.frame(MNprcp20km = rep(seq(minMNP.NC, maxMNP.NC, length.out = 20), times = 3), Individuals
_state = c(rep(0, times = 20), rep(1, times = 20), rep(2, times = 20)
))
PDP.MNP.SPC.NC$Individuals_state <- as.factor(PDP.MNP.SPC.NC$Individuals_state)

PDP.SST.SPC.C <- data.frame(SST = rep(seq(minSST.C, maxSST.C, length.out = 20), times = 3), Individuals_state =
c(rep(0, times = 20), rep(1, times = 20), rep(2, times = 20)
))
PDP.SST.SPC.C$Individuals_state <- as.factor(PDP.SST.SPC.C$Individuals_state)

PDP.ENS.SPC.C <- data.frame(ENSO = rep(seq(minENS.C, maxENS.C, length.out = 20), times = 3), Individuals_state =
c(rep(0, times = 20), rep(1, times = 20), rep(2, times = 20)
))
PDP.ENS.SPC.C$Individuals_state <- as.factor(PDP.ENS.SPC.C$Individuals_state)

PDP.ENS.SPC.NC <- data.frame(ENSO = rep(seq(minENS.NC, maxENS.NC, length.out = 20), times = 3), Individuals_state
= c(rep(0, times = 20), rep(1, times = 20), rep(2, times = 20)
))
PDP.ENS.SPC.NC$Individuals_state <- as.factor(PDP.ENS.SPC.NC$Individuals_state)

PDP.KDE.SPC.C <- data.frame(pop.KDE = rep(seq(minPOP.C, maxPOP.C, length.out = 20), times = 3), Individuals_state
= c(rep(0, times = 20), rep(1, times = 20), rep(2, times = 20)
))
PDP.KDE.SPC.C$Individuals_state <- as.factor(PDP.KDE.SPC.C$Individuals_state)

PDP.KDE.SPC.NC <- data.frame(pop.KDE = rep(seq(minPOP.NC, maxPOP.C, length.out = 20), times = 3), Individuals_sta
te = c(rep(0, times = 20), rep(1, times = 20), rep(2, times = 20)
))
PDP.KDE.SPC.NC$Individuals_state <- as.factor(PDP.KDE.SPC.NC$Individuals_state)
```

As part of model evaluation, we will calculate the area under the curve score. To do so we need to use the RF models we create to predict yes or no trauma for each individual in the dataset. Predict with ranger is a non-standard predict function, so we will write our own to use for this.

```
pred <- function(model, newdata)  {
  results <- as.data.frame(predict(model, newdata)$predictions)
  return(results)
}
```

Step 3. Random Forest Model Analysis

To analyze the data we make use of random forest (Breiman 2001) logistic regression. Models estimate the probability that an individual will not experience violent trauma during their lifetime by testing climate, demographic, and socio-political variables that might structure that probability. Models are partitioned into two geographic categories, coastal and non-coastal, adapted from Wilson et al. (2022) and Pulgar Vidal (1981). The coastal zone contains sites <15km from the coast and under 500 masl. The mid-elevation/highland zone contains sites >15km from the coast or above 500 masl. This division is designed to distinguish demographic trends between coastal and inland zones and allow for the inclusion or exclusion of Sea Surface Temperature (SST) from inland sites in favor of Standard Deviation in Temperature. Since our terrestrial climate, oceanic climate, and socio-political variables are estimated based on proximity to skeletal samples (using Euclidean distance or a 20km buffer), these measures are designed to account for continuous variation across environmental gradients.

To assess our predictions, we use Random Forest (RF) logistic regression (Breiman 2001; Liaw and Wiener 2002; Hastie et al. 2009), a machine learning ensemble decision tree method. We use RF because RF models can handle, and identify, interactions between variables, work with variables with autocorrelation, and evaluate non-linear relationships through subsampling predictor variables to prevent over-reliance on a single predictor. This helps limit the ability for RF to overfit (Breiman 2001; Stroble et al. 2009; Valavi et al. 2021). We implement a regression approach as a) our objective in statistically modeling the data is not to predict future sites but to understand the pattern(s), if any, that exist within the extant data set and b) as regression estimates probability forests (Wright and Ziegler 2017) that estimate probabilities more accurately (Malley et al. 2012) and enable us to more directly assess model discrimination (Zhang et al. 2019) while continuing to address potential overfitting (Valavi et al. 2021).

Two sets of RF models are created for the analysis, one each for coastal and inland individuals. We run separate models because the aspects of climate and environment differ from the coasts versus inland areas. To control for the temporal uncertainty of individuals in our dataset, we propagate temporal uncertainty into the RF modeling approach through running 2000 unique models, 1000 each for coastal and inland data subsets using each of the 1000 resampled datapoints per individual. In effect this treats every observation from the Monte Carlo sampling procedure as a 'true' model, enabling us to evaluate the aggregate output of the 1000 models for underlying patterns that span the possible climate and population combinations.

Although the individual trauma record data is imbalanced (~75-80% individuals have no trauma) it is not prohibitively imbalanced for a regression approach. Beyond this though, we must deal with the fact that any individuals in our database from the same site share similar predictor variable values, and some sites have >100 individuals while others only have a few. While the larger sites should carry some additional weight in the model, excessive imbalance could potentially skew results. To control for this, in each of the 1000 model runs, we randomly select a maximum of 30 individuals per site to be incorporated into the model. This ensures that, in each model, all sites are represented, that greater weight is provided to individuals from sites with larger sample sizes, and that we limit the effect of large sites to ensure model signals do reflect only patterns from the largest sites. This omitting of a random portion of data in each model also aids in avoiding model overfitting. To assist with evaluation of model performance we implement train and test splitting.

To directly assess our predictions, we must generate partial responses (i.e., how a change in a predictor variable impacts the likelihood of trauma or no trauma) for all RF models. As the response variable is binomial we use RF probability trees, meaning the output is an estimation of the probability, for each individual, that they will have or not have trauma (e.g., the probability that they are in the no trauma or trauma class). Model performance is assessed using mean square error (MSE) of the prediction and area under the curve based upon a model trained on training data and then predicting the test (hold-out) dataset. After generating the train-test model performance we generate a second model including all data from which we measure logloss accuracy, as a probability, to capture total model performance and generate partial dependence plots estimating the probability an individual will not have trauma across the range of each predictor variable while the others' effects are held constant. We also, for 100 models for both coastal and inland, generate three-dimensional partial response plots to show the interactive effects of climate variables and population on the probability of no trauma. We also generate 3-dimensional response plots for the interactions between sociopolitical complexity and climate and population. Due to time and computational constraints, we are unable to run the three-dimensional responses for every model as it is very computationally intensive. The output of this procedure is, for each region, 1000 unique estimates of the ability of the climate, population, and sociopolitical complexity variables to explain whether an individual experienced trauma or not, with temporal uncertainty included. Assessment of predictions relies upon the aggregated model performance metrics and partial responses. Visualizations of all 2000 model single variable partial responses are provided in S2. 3d partial responses visualized in our results are the aggregate results of the 200 models for which 3d partial response were generated.

The model analysis process is a memory (RAM) intensive process and is slow. We ran this on the Center for High Performance Computing (we did the same for both climate and population resampling) cluster at the Univeristy of Utah. The code below will work on a sufficiently powerful home computing device but may take a long time to run. If running on a home device be sure to adjust the number of cores (ncores) accordingly.

```
#We are going to run 1000 RF models to capture the range of variability in climate and population reconstructions
and use the output from each of the 1k models in our final analysis. Setting this sequence number lets us read in
just the rows we need for each RF model to save on RAM.
from.seq <- seq(from = 1, to = 7992000, by = 7992)
to.seq <- seq(from = 7992, to = 7992000, by = 7992)

start <- Sys.time()

ncores <- 16
my.cluster <- makeCluster(ncores)
doParallel::registerDoParallel(cl = my.cluster)
foreach(i = 1:1000, .packages = c("ranger","fst", "pROC", "data.table", "pdp", "dplyr", "yardstick", "caTools"))
%dopar%
  {
    #Get data in batches of 7992 (total individuals) so that each batch is 1 run
    indv.data <- read.fst("./data/indv.data.full.fst",
                          from = from.seq[i], to = to.seq[i],
                          column = c("Site.and.location", "total_trauma", "MNtemp20km",
                                     "MNprcp20km", "pop.KDE", "Individuals_state",
                                     "Region_Fact", "SST", "ENSO"))

#To run this without subsampling the sites - simply remove the code generating n.obs thorugh the indv.data %>% sa
mple_n(frq[1])

    #get the number of individuals per site
    n.obs <- indv.data %>%
      group_by(Site.and.location) %>%
      count(Site.and.location)
    #arrange the dataset by site.and.location to match with the output from count()
    indv.data <- indv.data %>%
      group_by(Site.and.location) %>%
      arrange(Site.and.location)
    #set a new column to be frq where that value is the number of individuals at the site
    indv.data$frq <- rep(n.obs$n, n.obs$n)
    indv.data$frq[which(indv.data$frq > 30)] <- 30
    #sample the dataframe taking no more than 30 individuals from a site (less if there are less than 30)
    indv.data <- indv.data %>%
      sample_n(frq[1])
    #
    #Select the individuals just from one region
    indv.data <- indv.data[which(indv.data$Region_Fact == "coastal"),]

    #Remove the Region_Fact variable
    indv.data <- subset(indv.data, select = -c(Region_Fact, Site.and.location, frq))
    #indv.data <- subset(indv.data, select = -c(Region_Fact, Site.and.location))

    indv.data <- indv.data[which(!is.na(indv.data$total_trauma)),]#drop NAs for trauma
    #indv.data$total_trauma <- factor(indv.data$total_trauma, levels = c("0", "1"))#set trauma factor level
    indv.data$Individuals_state <- factor(indv.data$Individuals_state, levels = c(0,1,2))#set state var as a fact
or level

    #Split the sample, preserving relative ratios of presence / absence of trauma
    split <- sample.split(indv.data$total_trauma, SplitRatio = 0.8) #train on 80% of data following standard cust
oms

    #add split TRUE / FALSE to the data
    indv.data$split <- split

    #Split the data into training and testing subsets
    data.train <- subset(indv.data, split == "TRUE")
    data.test <- subset(indv.data, split == "FALSE")

    #Remove the Split column
    data.train <- data.train[,-8]
    data.test <- data.test[,-8]

    #drop Split column from indv data
    indv.data <- indv.data[,-8]

    #regression with ranger
    RF.rang <- ranger(total_trauma ~ ., data = data.train, num.trees = 3000, mtry = 2, replace = TRUE, importance
= "permutation", min.node.size = 10)#General heuristics for choosing mtry value are square root of total # featur
es, 1/3 of the total # features, or log base 2 of the total # features. These all give a value between 1.98 and
2.5 - so we will do mtry of 2.

    #use the train data model to predict test data for model performance
    RF.rang.pred <- pred(RF.rang, newdata = data.test)

    #generate the reciever operating characteristic
    roctest <- roc(data.test$total_trauma, RF.rang.pred$`predict(model, newdata)$predictions`)
    #extract the area under the curve value
    aucRF <- pROC::auc(roctest)

    #calculate model logloss
    mod.logloss <-  ModelMetrics::logLoss(actual = data.test$total_trauma, pred = RF.rang.pred$`predict(model, ne
wdata)$predictions`)
    #conver model logloss to probability
   mod.logloss.prob <- exp(-ModelMetrics::logLoss(actual = data.test$total_trauma, pred = RF.rang.pred$`predict(m
odel, newdata)$predictions`))

    #run the model with all data
    RF.rang.all <- ranger(total_trauma ~ ., data = indv.data, num.trees = 3000, mtry = 2, replace = TRUE, importa
nce = "permutation", min.node.size = 10)

    #predict output for model evaluation on logloss
    RF.rang.pred.all <- pred(RF.rang, newdata = indv.data)

    #obtain model roc
    roctest.all <- roc(indv.data$total_trauma, RF.rang.pred.all$`predict(model, newdata)$predictions`)
    #extract model auc
    aucRF.all <- pROC::auc(roctest.all)

    #calculate the logloss of the model with all data included to measure overall model accuracy
    mod.logloss.all <-  ModelMetrics::logLoss(actual = indv.data$total_trauma, pred = RF.rang.pred.all$`predict(m
odel, newdata)$predictions`)
    #get the probability of correct class assignment
    mod.logloss.prob.all <- exp(-ModelMetrics::logLoss(actual = indv.data$total_trauma, pred = RF.rang.pred.all$`
predict(model, newdata)$predictions`))

    #generate the 2-dimensional partial response plots
    PDP.MNT.partial.C <- pdp::partial(RF.rang.all, pred.var = c("MNtemp20km"), pred.grid = PDP.MNT.C, prob = TRU
E)
    PDP.MNP.partial.C <- pdp::partial(RF.rang.all, pred.var = c("MNprcp20km"), pred.grid = PDP.MNP.C, prob = TRU
E)
    PDP.POP.partial.C <- pdp::partial(RF.rang.all, pred.var = c("pop.KDE"), pred.grid = PDP.POP.C, prob = TRUE)
    PDP.SST.partial.C <- pdp::partial(RF.rang.all, pred.var = c("SST"), pred.grid = PDP.SST.C, prob = TRUE)
    PDP.ENS.partial.C <- pdp::partial(RF.rang.all, pred.var = c("ENSO"), pred.grid = PDP.ENS.C, prob = TRUE)
    PDP.STT.partial.C <- pdp::partial(RF.rang.all, pred.var = c("Individuals_state"), pred.grid = PDP.STT.C, prob
= TRUE)

    #Put all the model evaluation data into one dataframe
    RF.results <- data.table(
      MSE.prederr = RF.rang$prediction.error,
      AUC = aucRF,
      AUC.all = aucRF.all,
      mod.logloss = mod.logloss,
      mod.logloss.prob = mod.logloss.prob,
      MSE.prederr.all = RF.rang.all$prediction.error,
      mod.logloss.all = mod.logloss.all,
      mod.logloss.prob.all = mod.logloss.prob.all,
      MNT_Imp = RF.rang$variable.importance[1],
      MNP_Imp = RF.rang$variable.importance[2],
      POP_Imp = RF.rang$variable.importance[3],
      SST_Imp = RF.rang$variable.importance[4],
      ENS_Imp = RF.rang$variable.importance[5],
      STT_Imp = RF.rang$variable.importance[6])

        #Generate the 3 dimensional partial predictions for 100 repetitions (we only generate 100 samples of this
because of computational time involved: ~40 hours of computational time to get the 100 repetitions)
    if (i <= 100) {
    MNT.KDE.PDP.C <- pdp::partial(RF.rang.all, pred.var = c("MNtemp20km", "pop.KDE"), pred.grid = PDP.MNT.POP.C,
plot = FALSE)
    MNP.KDE.PDP.C <- pdp::partial(RF.rang.all, pred.var = c("MNprcp20km", "pop.KDE"), pred.grid = PDP.MNP.POP.C,
plot = FALSE)
    SST.KDE.PDP.C <- pdp::partial(RF.rang.all, pred.var = c("SST", "pop.KDE"), pred.grid = PDP.SST.POP.C, plot =
FALSE)
    ENS.KDE.PDP.C <- pdp::partial(RF.rang.all, pred.var = c("ENSO", "pop.KDE"), pred.grid = PDP.ENS.POP.C, plot =
FALSE)
    MNP.MNT.PDP.C <- pdp::partial(RF.rang.all, pred.var = c("MNtemp20km", "MNprcp20km"), pred.grid = PDP.MNT.MNP.
C, plot = FALSE)

    MNT.SPC.PDP.C <- pdp::partial(RF.rang.all, pred.var = c("MNtemp20km", "Individuals_state"), pred.grid = PDP.M
NT.SPC.C, plot = FALSE)
    MNP.SPC.PDP.C <- pdp::partial(RF.rang.all, pred.var = c("MNprcp20km", "Individuals_state"), pred.grid = PDP.M
NP.SPC.C, plot = FALSE)
    SST.SPC.PDP.C <- pdp::partial(RF.rang.all, pred.var = c("SST", "Individuals_state"), pred.grid = PDP.SST.SPC.
C, plot = FALSE)
    ENS.SPC.PDP.C <- pdp::partial(RF.rang.all, pred.var = c("ENSO", "Individuals_state"), pred.grid = PDP.ENS.SP
C.C, plot = FALSE)
    KDE.SPC.PDP.C <- pdp::partial(RF.rang.all, pred.var = c("pop.KDE", "Individuals_state"), pred.grid = PDP.KDE.
SPC.C, plot = FALSE)

    #Put 3d results in a df
    Partials.3d <- data.frame(
      MNT.KDE.PDP.C = MNT.KDE.PDP.C,
      MNP.KDE.PDP.C = MNP.KDE.PDP.C,
      SST.KDE.PDP.C = SST.KDE.PDP.C,
      ENS.KDE.PDP.C = ENS.KDE.PDP.C,
      MNP.MNT.PDP.C = MNP.MNT.PDP.C
    )
    Partials.3d.spc <- data.frame(
```

```r
      MNT.SPC.PDP.C = MNT.SPC.PDP.C,
      MNP.SPC.PDP.C = MNP.SPC.PDP.C,
      SST.SPC.PDP.C = SST.SPC.PDP.C,
      ENS.SPC.PDP.C = ENS.SPC.PDP.C,
      KDE.SPC.PDP.C = KDE.SPC.PDP.C
    )
    #Make hte file path to save the results
    mod_3dpdp.file <- paste("./output/3DPDP_C/model_part_", i, ".Rdata", sep = "")
    mod_3dpdp.spc.file <- paste("./output/3DPDP_C_SPC/model_part_", i, ".Rdata", sep = "")

    #Save the results
    save(Partials.3d, file = mod_3dpdp.file)
    save(Partials.3d.spc, file = mod_3dpdp.spc.file)

    #Remove results from environment to save space
    rm(list = c("MNT.KDE.PDP.C", "MNP.KDE.PDP.C", "SST.KDE.PDP.C", "ENS.KDE.PDP.C", "MNP.MNT.PDP.C", "Partials.3d", "mod_3dpdp.file", "MNT.SPC.PDP.C", "MNP.SPC.PDP.C", "SDT.SPC.PDP.C", "ENS.SPC.PDP.C", "KDE.SPC.PDP.C", "mod_3dpdp.spc.file"))

    }

    #make a df of single variable partial responses
    PDP.partials <- data.frame(
          PDP.MNT.C = PDP.MNT.partial.C,
          PDP.MNP.C = PDP.MNP.partial.C,
          PDP.POP.C = PDP.POP.partial.C,
          PDP.SST.C = PDP.SST.partial.C,
          PDP.ENS.C = PDP.ENS.partial.C,
          PDP.STT.C = PDP.STT.partial.C
                                  )
    #Make file path for model performance data
    mod_perf.file <- paste("./output/Performance_C/model_perf_", i, ".Rdata", sep = "")
    #make file path for single var partial response data
    mod_pdp_part.file <- paste("./output/PDP_Partials_C/model_part_",i,".Rdata", sep = "")
    #save performance and single partials
    save(PDP.partials, file = mod_pdp_part.file)
    save(RF.results, file = mod_perf.file)

    #clean the environment to hopefully cut down on the RAM used which is causing failures
    rm(list = c("indv.data", "RF.rang", "RF.rang.all", "RF.rang.pred", "RF.rang.pred.all", "roctest", "aucRF", "roctest.all", "aucRF.all", "RF.results", "PDP.MNT.partial.C", "PDP.MNP.partial.C", "PDP.POP.partial.C", "PDP.SST.partial.C", "PDP.ENS.partial.C", "PDP.STT.partial.C", "mod_perf.file", "mod_pdp_part.file", "mod.logloss", "mod.logloss.prob", "mod.logloss.all", "mod.logloss.prob.all"))

    gc(verbose = FALSE)#garbage collect to free up cleaned memory space


  }
stopCluster(my.cluster)

#Repeat for non-coastal

ncores <- 16
my.cluster <- makeCluster(ncores)
doParallel::registerDoParallel(cl = my.cluster)
foreach(i = 1:1000, .packages = c("ranger","fst", "pROC", "data.table", "pdp", "dplyr", "yardstick", "caTools"))
%dopar% #"pdp"
  {

    #Get data in batches of 7992 (total individuals) so that each batch is 1 run
    indv.data <- read.fst("./data/indv.data.full.fst",
                          from = from.seq[i], to = to.seq[i],
                          column = c("Site.and.location" ,"total_trauma", "MNtemp20km",  "MNprcp20km",
                                     "pop.KDE", "SDtemp20km", "ENSO","Individuals_state", "Region_Fact"))

    # get the number of individuals per site
    n.obs <- indv.data %>%
      group_by(Site.and.location) %>%
      count(Site.and.location)
    #arrange the dataset by site.and.location to match with the output from count()
    indv.data <- indv.data %>%
      group_by(Site.and.location) %>%
      arrange(Site.and.location)
    #set a new column to be frq where that value is the number of individuals at the site
    indv.data$frq <- rep(n.obs$n, n.obs$n)
    indv.data$frq[which(indv.data$frq > 30)] <- 30
    #sample the dataframe taking no more than 30 individuals from a site (less if there are less than 30)
    indv.data <- indv.data %>%
      sample_n(frq[1])

    #Select the individuals just from one region
    indv.data <- indv.data[which(indv.data$Region_Fact == "non-coastal"),]

    #Remove the Region_Fact variable
    indv.data <- subset(indv.data, select = -c(Region_Fact, Site.and.location, frq))
    #indv.data <- subset(indv.data, select = -c(Region_Fact, Site.and.location))

    indv.data <- indv.data[which(!is.na(indv.data$total_trauma)),]#drop NAs for trauma
   # indv.data$total_trauma <- factor(indv.data$total_trauma, levels = c("0", "1"))#set trauma factor level
    indv.data$Individuals_state <- factor(indv.data$Individuals_state, levels = c(0,1,2))#set state var as a factor level

    #Split the sample, preserving relative ratios of presence / absence of trauma
    split <- sample.split(indv.data$total_trauma, SplitRatio = 0.8) #train on 80% of data following standard customs

    #add split TRUE / FALSE to the data
    indv.data$split <- split

    #Split the data into training and testing subsets
    data.train <- subset(indv.data, split == "TRUE")
    data.test <- subset(indv.data, split == "FALSE")

    #Remove the Split column
    data.train <- data.train[,-8]
    data.test <- data.test[,-8]

    #drop Split column from indv data
    indv.data <- indv.data[,-8]


    RF.rang <- ranger(total_trauma ~ ., data = data.train, num.trees = 3000, mtry = 2, replace = TRUE, importance = "permutation", min.node.size = 10)#General heuristics for choosing mtry value are square root of total # features, 1/3 of the total # features, or log base 2 of the total # features. These all give a value between 1.98 and 2.5 - so we will do mtry of 2.

    #predict
    RF.rang.pred <- pred(RF.rang, newdata = data.test)

    #get roc
    roctest <- roc(data.test$total_trauma, RF.rang.pred$`predict(model, newdata)$predictions`)
    #extract auc
    aucRF <- pROC::auc(roctest)

    #calculate logloss
     mod.logloss <-  ModelMetrics::logLoss(actual = data.test$total_trauma, pred = RF.rang.pred$`predict(model, newdata)$predictions`)
   #convert to probability correct prediction
     mod.logloss.prob <- exp(-ModelMetrics::logLoss(actual = data.test$total_trauma, pred = RF.rang.pred$`predict(model, newdata)$predictions`))

    #rerun with all data
    RF.rang.all <- ranger(total_trauma ~ ., data = indv.data, num.trees = 3000, mtry = 2, replace = TRUE, importance = "permutation", min.node.size = 10)

    #predict
    RF.rang.pred.all <- pred(RF.rang.all, newdata = indv.data)

    #calculate roc
    roctest.all <- roc(indv.data$total_trauma, RF.rang.pred.all$`predict(model, newdata)$predictions`)
    #extract auc
    aucRF.all <- pROC::auc(roctest.all)

    #calculate logloss for model accuracy
    mod.logloss.all <-  ModelMetrics::logLoss(actual = indv.data$total_trauma, pred = RF.rang.pred.all$`predict(model, newdata)$predictions`)
    #convert logloss to probability of accurate prediction
    mod.logloss.prob.all <- exp(-ModelMetrics::logLoss(actual = indv.data$total_trauma, pred = RF.rang.pred.all$`predict(model, newdata)$predictions`))

    #generate 2-dimensional partial response data
    PDP.MNT.partial.NC <- pdp::partial(RF.rang.all, pred.var = c("MNtemp20km"), pred.grid = PDP.MNT.NC, prob = TRUE)
    PDP.MNP.partial.NC <- pdp::partial(RF.rang.all, pred.var = c("MNprcp20km"), pred.grid = PDP.MNP.NC, prob = TRUE)
    PDP.POP.partial.NC <- pdp::partial(RF.rang.all, pred.var = c("pop.KDE"), pred.grid = PDP.POP.NC, prob = TRUE)
    PDP.SDT.partial.NC <- pdp::partial(RF.rang.all, pred.var = c("SDtemp20km"), pred.grid = PDP.SDT.NC, prob = TRUE)
    PDP.ENS.partial.NC <- pdp::partial(RF.rang.all, pred.var = c("ENSO"), pred.grid = PDP.ENS.NC, prob = TRUE)
    PDP.STT.partial.NC <- pdp::partial(RF.rang.all, pred.var = c("Individuals_state"), pred.grid = PDP.STT.NC, prob = TRUE)

    #Put all the model evaluation data into one dataframe
    RF.results <- data.table(
      MSE.prederr = RF.rang$prediction.error,
      AUC = aucRF,
      AUC.all = aucRF.all,
      mod.logloss = mod.logloss,
      mod.logloss.prob = mod.logloss.prob,
      MSE.prederr.all = RF.rang.all$prediction.error,
      mod.logloss.all = mod.logloss.all,
      mod.logloss.prob.all = mod.logloss.prob.all,
      MNT_Imp = RF.rang$variable.importance[1],
      MNP_Imp = RF.rang$variable.importance[2],
      POP_Imp = RF.rang$variable.importance[3],
      SDT_Imp = RF.rang$variable.importance[4],
      ENS_Imp = RF.rang$variable.importance[5],
      STT_Imp = RF.rang$variable.importance[6])

    #
    # #Generate the 3 dimensional partial predictions for 100 repetitions
    if (i <= 100) {
    MNT.KDE.PDP.NC <- pdp::partial(RF.rang.all, pred.var = c("MNtemp20km", "pop.KDE"), pred.grid = PDP.MNT.POP.NC, plot = FALSE)
    MNP.KDE.PDP.NC <- pdp::partial(RF.rang.all, pred.var = c("MNprcp20km", "pop.KDE"), pred.grid = PDP.MNP.POP.NC, plot = FALSE)
    SDT.KDE.PDP.NC <- pdp::partial(RF.rang.all, pred.var = c("SDtemp20km", "pop.KDE"), pred.grid = PDP.SDT.POP.NC, plot = FALSE)
    ENS.KDE.PDP.NC <- pdp::partial(RF.rang.all, pred.var = c("ENSO", "pop.KDE"), pred.grid = PDP.ENS.POP.NC, plot = FALSE)
    MNP.MNT.PDP.NC <- pdp::partial(RF.rang.all, pred.var = c("MNtemp20km", "MNprcp20km"), pred.grid = PDP.MNT.MNP.NC, plot = FALSE)

    MNT.SPC.PDP.NC <- pdp::partial(RF.rang.all, pred.var = c("MNtemp20km", "Individuals_state"), pred.grid = PDP.MNT.SPC.NC, plot = FALSE)
    MNP.SPC.PDP.NC <- pdp::partial(RF.rang.all, pred.var = c("MNprcp20km", "Individuals_state"), pred.grid = PDP.MNP.SPC.NC, plot = FALSE)
    SDT.SPC.PDP.NC <- pdp::partial(RF.rang.all, pred.var = c("SDtemp20km", "Individuals_state"), pred.grid = PDP.SDT.SPC.NC, plot = FALSE)
    ENS.SPC.PDP.NC <- pdp::partial(RF.rang.all, pred.var = c("ENSO", "Individuals_state"), pred.grid = PDP.ENS.SPC.NC, plot = FALSE)
    KDE.SPC.PDP.NC <- pdp::partial(RF.rang.all, pred.var = c("pop.KDE", "Individuals_state"), pred.grid = PDP.KDE.SPC.NC, plot = FALSE)

    #Put 3d results in a df
    Partials.3d <- data.frame(
      MNT.KDE.PDP.NC = MNT.KDE.PDP.NC,
      MNP.KDE.PDP.NC = MNP.KDE.PDP.NC,
      SDT.KDE.PDP.NC = SDT.KDE.PDP.NC,
      ENS.KDE.PDP.NC = ENS.KDE.PDP.NC,
      MNP.MNT.PDP.NC = MNP.MNT.PDP.NC
    )

    Partials.3d.spc <- data.frame(
      MNT.SPC.PDP.NC = MNT.SPC.PDP.NC,
      MNP.SPC.PDP.NC = MNP.SPC.PDP.NC,
      SDT.SPC.PDP.NC = SDT.SPC.PDP.NC,
      ENS.SPC.PDP.NC = ENS.SPC.PDP.NC,
      KDE.SPC.PDP.NC = KDE.SPC.PDP.NC
    )

    #Make hte file path to save the results
    mod_3dpdp.file <- paste("./output/Regression/3DPDP_NC/model_part_", i, ".Rdata", sep = "")
    mod_3dpdp.spc.file <- paste("./output/Regression/3DPDP_NC_SPC/model_part_", i, ".Rdata", sep = "")
    #Save the results
    save(Partials.3d, file = mod_3dpdp.file)
    save(Partials.3d.spc, file = mod_3dpdp.spc.file)
    #Remove results from environment to save space
    rm(list = c("MNT.KDE.PDP.NC", "MNP.KDE.PDP.NC", "SDT.KDE.PDP.NC", "ENS.KDE.PDP.NC", "MNP.MNT.PDP.NC", "Partials.3d", "mod_3dpdp.file", "MNT.SPC.PDP.NC", "MNP.SPC.PDP.NC", "SDT.SPC.PDP.NC", "ENS.SPC.PDP.NC", "KDE.SPC.PDP.NC", "mod_3dpdp.spc.file"))
    }

    #make a df of single variable partial responses
    PDP.partials <- data.frame(
          PDP.MNT.NC = PDP.MNT.partial.NC,
          PDP.MNP.NC = PDP.MNP.partial.NC,
          PDP.POP.NC = PDP.POP.partial.NC,
          PDP.SDT.NC = PDP.SDT.partial.NC,
          PDP.ENS.NC = PDP.ENS.partial.NC,
          PDP.STT.NC = PDP.STT.partial.NC
                                  )
    #Make file path for model performance data
    mod_perf.file <- paste("./output/Performance_NC/model_perf_", i, ".Rdata", sep = "")
    #make file path for single var partial response data
    mod_pdp_part.file <- paste("./output/PDP_Partials_NC/model_part_",i,".Rdata", sep = "")
    #save performance and single partials
    save(PDP.partials, file = mod_pdp_part.file)
    save(RF.results, file = mod_perf.file)

    #clean the environment to hopefully cut down on the RAM used which is causing failures
    rm(list = c("indv.data", "RF.rang", "RF.rang.all", "RF.rang.pred", "RF.rang.pred.all", "roctest", "aucRF", "roctest.all", "aucRF.all", "RF.results", "PDP.MNT.partial.NC", "PDP.MNP.partial.NC", "PDP.POP.partial.NC", "PDP.SDT.partial.NC", "PDP.ENS.partial.NC", "PDP.STT.partial.NC", "mod_perf.file", "mod_pdp_part.file", "mod.logloss", "mod.logloss.prob", "mod.logloss.all", "mod.logloss.prob.all"))

    gc(verbose = FALSE)#garbage collect to free up cleaned memory space

#    end <- Sys.time()
#time.total <- end - start
#time.total
  }

stopCluster(my.cluster)

    end <- Sys.time()
time.total <- end - start
time.total
```

We saved the output from each of the 1k model runs as its own file for ease of computation. Next, combine the relevant files together so that everything is in one place.

```r
#Repeat for model performance
fn1 <- list.files(path = "./output/Performance_C",
                  pattern = "Rdata",
                  full.names = TRUE)

all_performance.C <- data.table()

for (i in 1:length(fn1)) {
   load(fn1[i])
   RF.results <- data.table(RF.results)
   to.bind <- list(all_performance.C, RF.results)
   all_performance.C <- rbindlist(to.bind)
}

#now non-coastal model performance
fn2 <- list.files(path = "./output/Performance_NC",
                  pattern = "Rdata",
                  full.names = TRUE)

all_performance.NC <- data.table() #make an empty data frame to store the 10k set of partial responses

for (i in 1:length(fn2)) {
   load(fn2[i])
   RF.results <- data.table(RF.results)
   to.bind <- list(all_performance.NC, RF.results)
   all_performance.NC <- rbindlist(to.bind)
}

fn3 <- list.files(path = "./output/PDP_Partials_C",
                  pattern = "Rdata",
                  full.names = TRUE)#obtain a list of all of the files (individual tifs) in the folder and place them in a single vector

pdp_partials.C <- data.table() #make an empty data frame to store the 1k set of partial responses

for (i in 1:length(fn3)) {
   load(fn3[i])
   RF.partials <- data.table(PDP.partials)
   to.bind <- list(pdp_partials.C, RF.partials)
   pdp_partials.C <- rbindlist(to.bind)
}


#Repeat for model performance
fn4 <- list.files(path = "./output/PDP_Partials_NC",
                  pattern = "Rdata",
                  full.names = TRUE)

pdp_partials.NC <- data.table()

for (i in 1:length(fn4)) {
   load(fn4[i])
   RF.partials <- data.table(PDP.partials)
   to.bind <- list(pdp_partials.NC, RF.partials)
   pdp_partials.NC <- rbindlist(to.bind)
}

 #now get 3 dimensional partial response for coastal output
 fn5 <- list.files(path = "./output/3DPDP_C",
                   pattern = "Rdata",
                   full.names = TRUE)

 pdp3d_partials.C <- data.table()

 for (i in 1:length(fn5)) {
    load(fn5[i])
    PDP.partials <- data.table(Partials.3d)
    to.bind <- list(pdp3d_partials.C, PDP.partials)
    pdp3d_partials.C <- rbindlist(to.bind)
 }

 #repeat for non-coastal 3d partials
 fn6 <- list.files(path = "./output/3DPDP_NC",
                   pattern = "Rdata",
                   full.names = TRUE)

 pdp3d_partials.NC <- data.table()

 for (i in 1:length(fn6)) {
    load(fn6[i])
    PDP.partials <- data.table(Partials.3d)
    to.bind <- list(pdp3d_partials.NC, PDP.partials)
    pdp3d_partials.NC <- rbindlist(to.bind)
 }

 #now get 3 dimensional partial response for coastal output SPC
 fn7 <- list.files(path = "./output/3DPDP_C_SPC",
                   pattern = "Rdata",
                   full.names = TRUE)

 pdp3d_spc.C <- data.table()

 for (i in 1:length(fn7)) {
    load(fn7[i])
    PDP.partials <- data.table(Partials.3d.spc)
    to.bind <- list(pdp3d_spc.C, Partials.3d.spc)
    pdp3d_spc.C <- rbindlist(to.bind)
 }


  #now get 3 dimensional partial response for coastal output SPC
 fn8 <- list.files(path = "./output/3DPDP_NC_SPC",
                   pattern = "Rdata",
                   full.names = TRUE)

 pdp3d_spc.NC <- data.table()

 for (i in 1:length(fn8)) {
    load(fn8[i])
    PDP.partials <- data.table(Partials.3d.spc)
    to.bind <- list(pdp3d_spc.NC, Partials.3d.spc)
    pdp3d_spc.NC <- rbindlist(to.bind)
 }



# #Save the aggregated outputs
 save(all_performance.C, file = "./output/all_performance.C.Rdata")
 save(all_performance.NC, file = "./output/all_performance.NC.Rdata")

 save(pdp_partials.C, file = "./output/pdp_partials.C.Rdata")
 save(pdp_partials.NC, file = "./output/pdp_partials.NC.Rdata")

 save(pdp3d_partials.C, file = "./output/pdp3d_partials.C.Rdata")
 save(pdp3d_partials.NC, file = "./output/pdp3d_partials.NC.Rdata")

 save(pdp3d_spc.C, file = "./output/pdp3d_spc.C.Rdata")
 save(pdp3d_spc.NC, file = "./output/pdp3d_spc.NC.Rdata")
```

Step 4. Analyse Model Output

Read in the saved aggregated model outputs.

```r
load("./output/all_performance.C.Rdata")
load("./output/all_performance.NC.Rdata")
load("./output/pdp_partials.C.Rdata")
load("./output/pdp_partials.NC.Rdata")
load("./output/pdp3d_partials.C.Rdata")
load("./output/pdp3d_partials.NC.Rdata")
load("./output/pdp3d_spc.C.Rdata")
load("./output/pdp3d_spc.NC.Rdata")
```

First we will look at model performance metrics mean square error and area under the curve scores for the test-train split versions of the model for evaluating if the model provides discriminatory information in discerning trauma or no trauma.

```r
#save the image files
#png(filename = "Coastal_OOB.png", width = 3, height = 4, units = "in", res = 300)
#pdf(file = "Coastal_MSE.pdf", width = 3, height = 4)
#hist(all_performance.C$MSE.prederr, main = "", xlab = "MSE Pred. Error", breaks = 15, col = "#2D708EFF", border = "white")
#dev.off()

#pdf(file = "Coastal_AUC.pdf", width = 3, height = 4)
#hist(all_performance.C$AUC, main = "", xlab = "AUC", breaks = 15, col = "#73D055FF", border = "white")
#dev.off()

#pdf(file = "Inland_MSE.pdf", width = 3, height = 4)
#hist(all_performance.NC$MSE.prederr, main = "", xlab = "MSE Pred. Error", breaks = 15, col = "#2D708EFF", border = "white")
#dev.off()

#pdf(file = "Inland_AUC.pdf", width = 3, height = 4)
#hist(all_performance.NC$AUC, main = "", xlab = "AUC", breaks = 15, col = "#73D055FF", border = "white")
#dev.off()

par(mfrow = c(2,2))
#par(pty = "s")
hist(all_performance.C$MSE.prederr, main = "", xlab = "MSE Pred. Error", breaks = 15, col = "#2D708EFF", border = "white")
mtext("Coastal", side = 3, line = 1)
hist(all_performance.NC$MSE.prederr, main = "", xlab = "MSE Pred. Error", breaks = 15, col = "#73D055FF", border = "white")
mtext("Non-Coastal", side = 3, line = 1)
hist(all_performance.C$AUC, main = "", xlab = "AUC", breaks = 15, col = "#2D708EFF", border = "white")
hist(all_performance.NC$AUC, main = "", xlab = "AUC", breaks = 15, col = "#73D055FF", border = "white")
```

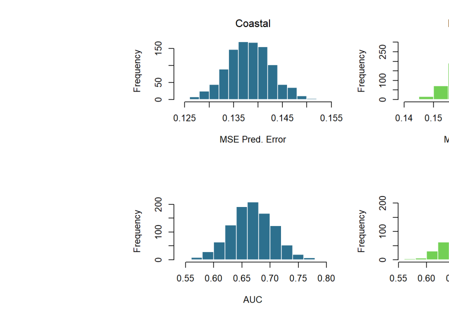

Supplemental Figure 21. Mean square error and area under the curve (AUC) model performance values for each of the 1000 RF models for the coast (left, in blue) and non-coastal (right, in green) individuals. AUC values suggest that the models for coast and non-coastal individuals perform acceptably well in predicting the absence or presence of trauma associated with violence and peace. Model outputs are normally distributed suggesting the central tendency of model performance accurately captures how strongly the climate and population data predict trauma.

Following standard practices, to capture total model accuracy performance we present the log-loss and model log-loss as probability of correct prediction from the models using all data.

```r
par(mfrow = c(2,2))
#par(pty = "s")
hist(all_performance.C$mod.logloss.all, main = "", xlab = "Log-Loss", breaks = 15, col = "#2D708EFF", border = "white", xlim = c(0.24,0.29))
mtext("Coastal", side = 3, line = 1)
hist(all_performance.NC$mod.logloss.all, main = "", xlab = "Log-Loss", breaks = 15, col = "#73D055FF", border = "white", xlim = c(0.24,0.29))
mtext("Non-Coastal", side = 3, line = 1)
hist(all_performance.C$mod.logloss.prob.all, main = "", xlab = "Log-Loss as Probability", breaks = 15, col = "#2D708EFF", border = "white", xlim = c(0.745,0.79))
hist(all_performance.NC$mod.logloss.prob.all, main = "", xlab = "Log-Loss as Probability", breaks = 15, col = "#73D055FF", border = "white", xlim = c(0.745,0.79))
```

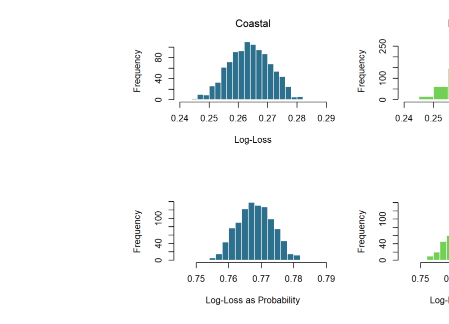

Supplemental Figure 22. In line with the AUC and MSE results, the log-loss results suggest the model performs acceptably well. We correctly predict trauma / no-trauma with around 77% accuracy; this is strong model performance given the data and the amount of uncertainty built into the models to capture temporal uncertainty, limit influence of large sites, and sample randomly for each split of the RF tree in each RF model.

Coastal Model Outcomes

Next we look at variable importance on the coast. This is calculated as the permutation variable importance, so it is the increase in MSE based upon permuting the variable out of the model.

```r
MNT_Imp <- mean(all_performance.C$MNT_Imp)#1st
MNT_t <- t.test(all_performance.C$MNT_Imp)

MNP_Imp <- mean(all_performance.C$MNP_Imp)#2nd
MNP_t <- t.test(all_performance.C$MNP_Imp)

POP_Imp <- mean(all_performance.C$POP_Imp)#5th
POP_t <- t.test(all_performance.C$POP_Imp)

SST_Imp <- mean(all_performance.C$SST_Imp)#6th
SST_t <- t.test(all_performance.C$SST_Imp)

ENS_Imp <- mean(all_performance.C$ENS_Imp)#3rd
ENS_t <- t.test(all_performance.C$ENS_Imp)

STT_Imp <- mean(all_performance.C$STT_Imp)#4th
STT_t <- t.test(all_performance.C$STT_Imp)

#png(filename = "Coastal.VarImp.png", height = 5, width = 5, units = "in", res = 300)
#pdf(file = "Coastal.VarImp.pdf", height = 5, width = 5)
par(pty = "s", oma = c(2,2,0,0))
plot(x = MNT_Imp, y = 6, xlim = c(0, 0.04), ylim = c(1,6), yaxt = "n", ylab = "", pch = 19, xlab = "", col = "#482576FF", cex = 1.5)
lines(x = c(-0.90, 0.0405), y = c(6,6), col = alpha("grey80", alpha = 0.5), lty = 2)
lines(x = c(-0.90, 0.0405), y = c(5,5), col = alpha("grey80", alpha = 0.5), lty = 2)
lines(x = c(-0.90, 0.0405), y = c(4,4), col = alpha("grey80", alpha = 0.5), lty = 2)
lines(x = c(-0.90, 0.0405), y = c(3,3), col = alpha("grey80", alpha = 0.5), lty = 2)
lines(x = c(-0.90, 0.0405), y = c(2,2), col = alpha("grey80", alpha = 0.5), lty = 2)
lines(x = c(-0.90, 0.0405), y = c(1,1), col = alpha("grey80", alpha = 0.5), lty = 2)

lines(x = c(0, 0), y = c(0.9,6.10), col = alpha("grey80", alpha = 0.5), lty = 2)
lines(x = c(0.01, 0.01), y = c(0.9,6.10), col = alpha("grey80", alpha = 0.5), lty = 2)
lines(x = c(0.02, 0.02), y = c(0.9,6.10), col = alpha("grey80", alpha = 0.5), lty = 2)
lines(x = c(0.03, 0.03), y = c(0.9,6.10), col = alpha("grey80", alpha = 0.5), lty = 2)
lines(x = c(0.04, 0.04), y = c(0.9,6.10), col = alpha("grey80", alpha = 0.5), lty = 2)

lines(x = c(mean(all_performance.C$MNP_Imp) + sd(all_performance.C$MNP_Imp)*2, mean(all_performance.C$MNP_Imp) - sd(all_performance.C$MNP_Imp)*2), y = c(5,5), col = "black")
lines(x = c(mean(all_performance.C$SST_Imp) + sd(all_performance.C$SST_Imp)*2, mean(all_performance.C$SST_Imp) - sd(all_performance.C$SST_Imp)*2), y = c(1,1), col = "black")
lines(x = c(mean(all_performance.C$MNT_Imp) + sd(all_performance.C$MNT_Imp)*2, mean(all_performance.C$MNT_Imp) - sd(all_performance.C$MNT_Imp)*2), y = c(6,6), col = "black")
lines(x = c(mean(all_performance.C$POP_Imp) + sd(all_performance.C$POP_Imp)*2, mean(all_performance.C$POP_Imp) - sd(all_performance.C$POP_Imp)*2), y = c(2,2), col = "black")
lines(x = c(mean(all_performance.C$ENS_Imp) + sd(all_performance.C$ENS_Imp)*2, mean(all_performance.C$ENS_Imp) - sd(all_performance.C$ENS_Imp)*2), y = c(4,4), col = "black")
lines(x = c(mean(all_performance.C$STT_Imp) + sd(all_performance.C$STT_Imp)*2, mean(all_performance.C$STT_Imp) - sd(all_performance.C$STT_Imp)*2), y = c(3,3), col = "black")
lines(x = c(MNT_t$conf.int[1], MNT_t$conf.int[2]), y = c(6,6), col = "white", lwd = 1)
points(x = MNP_Imp, y = 5, pch = 19, cex = 1.5, col = "#31668EFF")
lines(x = c(MNP_t$conf.int[1], MNP_t$conf.int[2]), y = c(5,5), col = "white")
points(x = ENS_Imp, y = 4, pch = 19, cex = 1.5, col = "#6BCD5AFF")
lines(x = c(ENS_t$conf.int[1], ENS_t$conf.int[2]), y = c(4,4), col = "white")
points(x = STT_Imp, y = 3, pch = 19, cex = 1.5, col = "black")
lines(x = c(STT_t$conf.int[1], STT_t$conf.int[2]), y = c(3,3), col = "white")
points(x = POP_Imp, y = 2, pch = 19, cex = 1.5, col = "#FDE725FF")
lines(x = c(POP_t$conf.int[1], POP_t$conf.int[2]), y = c(2,2), col = "white")
points(x = SST_Imp, y = 1, pch = 19, cex = 1.5, col = "#1E9C89FF")
lines(x = c(SST_t$conf.int[1], SST_t$conf.int[2]), y = c(1,1), col = "white")
axis(2, at = c(1,2,3,4,5,6), labels = FALSE, tick = TRUE)
mtext("Mean Temp.", at = 6, side = 2, line = 1, las = 1)
mtext("Mean Precip.", at = 5, side = 2, line = 1, las = 1)
mtext("ENSO Freq.", at = 4, side = 2, line = 1, las = 1)
mtext("State", at = 3, side = 2, line = 1, las = 1)
mtext("Population (KDE)", at = 2, side = 2, line = 1, las = 1)
mtext("Sea Surf. Temp.", at = 1, side = 2, line = 1, las = 1)
mtext("Variable Importance", side = 1, line = 2)
```

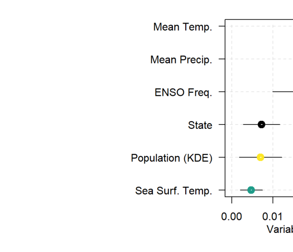

Supplemental Figure 23. Variable importance for coastal models. Colored dots provide the central tendency value for variable importance from the aggregate 1000 models, black lines are two standard deviations around the mean,and the white line provides the 95% confidence interval on the mean. Results suggest that climate matters more than social and demographic variables in predicting the presence/absence of trauma. However, variable importance does not evaluate our predictions. For that we use 2 and 3 dimensional partial response plots. We begin with the 2d plots for each of the coastal independent variables.

Mean Precip

```r
#Get the aggregated predicted responses and make info for a confidence interval for precip
MNP.list.C <- list(pdp_partials.C$PDP.MNP.C.MNprcp20km)
MNP.resp.ST1.C <- aggregate(1-pdp_partials.C$PDP.MNP.C.yhat, by = MNP.list.C, FUN = mean)#Get the average predicted probability no trauma for each precip value
MNP.resp.ST1.sdE.C <- aggregate(1-pdp_partials.C$PDP.MNP.C.yhat, by = MNP.list.C, FUN = sd)#Get the st dev in predicted probability no trauma for each precip value
MNP.margin.ST1.C <- qt(0.975, df = length(MNP.resp.ST1.C$Group.1)-1) * MNP.resp.ST1.sdE.C$x/sqrt(length(MNP.resp.ST1.C$Group.1))#Calculate 95% CIs
MNP.lower.int.ST1.C <- MNP.resp.ST1.C$x - MNP.margin.ST1.C#get the lower 95% CI boundary
MNP.upper.int.ST1.C <- MNP.resp.ST1.C$x + MNP.margin.ST1.C#get the upper 95% CI boundary

from.seq.plot <- seq(1, 21000, 21)
to.seq.plot <- seq(21, 21000, 21)

par(pty = "s")
plot(MNP.resp.ST1.C$x~MNP.resp.ST1.C$Group.1, type = "l", ylab = "Prob. of Peace \n(no trauma)", xlab = "Mean Precip.", ylim = c(0.65, 0.875), col = "#31668EFF")
for (i in 1:length(from.seq.plot)) {
  lines(1-pdp_partials.C$PDP.MNP.C.yhat[from.seq.plot[i]:to.seq.plot[i]]~pdp_partials.C$PDP.MNP.C.MNprcp20km[1:21], col = alpha("grey50", alpha = 0.15), lwd = 0.5)
}
lines(MNP.resp.ST1.C$x~MNP.resp.ST1.C$Group.1, col = "#31668EFF")
polygon(x = c(MNP.resp.ST1.C$Group.1, rev(MNP.resp.ST1.C$Group.1)), y = c(MNP.lower.int.ST1.C, rev(MNP.upper.int.ST1.C)), col = alpha("#31668EFF", alpha = 0.5), border = NA)
```

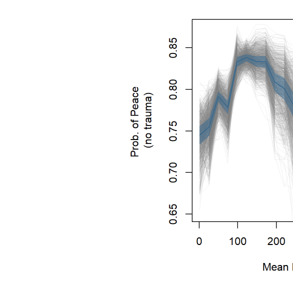

Supplemental Figure 24. Partial response of the probability of peace to variation in mean precipitation on the coast while all other predictor variables' effects are held constant.

Mean Temp

```r
MNT.list.C <- list(pdp_partials.C$PDP.MNT.C.MNtemp20km)
MNT.resp.ST1.C <- aggregate(1-pdp_partials.C$PDP.MNT.C.yhat, by = MNT.list.C, FUN = mean)
MNT.resp.ST1.sdE.C <- aggregate(1-pdp_partials.C$PDP.MNT.C.yhat, by = MNT.list.C, FUN = sd)
MNT.margin.ST1.C <- qt(0.975, df = length(MNT.resp.ST1.C$Group.1)-1) * MNT.resp.ST1.sdE.C$x/sqrt(length(MNT.resp.ST1.C$Group.1))#Calculate 95% CIs
MNT.lower.int.ST1.C <- MNT.resp.ST1.C$x - MNT.margin.ST1.C#get the lower 95% CI boundary
MNT.upper.int.ST1.C <- MNT.resp.ST1.C$x + MNT.margin.ST1.C#get the upper 95% CI boundary

#from.seq.plot <- seq(1, 2100, 21)
#to.seq.plot <- seq(21, 2100, 21)

par(pty = "s")
plot(MNT.resp.ST1.C$x~MNT.resp.ST1.C$Group.1, type = "l", ylab = "Prob. of Peace \n(no trauma)", xlab = "Mean Temp.", ylim = c(0.65, 0.875), col = "#482576FF")
for (i in 1:length(from.seq.plot)) {
  lines(1-pdp_partials.C$PDP.MNT.C.yhat[from.seq.plot[i]:to.seq.plot[i]]~pdp_partials.C$PDP.MNT.C.MNtemp20km[1:21], col = alpha("grey50", alpha = 0.15), lwd = 0.5)
}
lines(MNT.resp.ST1.C$x~MNT.resp.ST1.C$Group.1, col = "#482576FF")
polygon(x = c(MNT.resp.ST1.C$Group.1, rev(MNT.resp.ST1.C$Group.1)), y = c(MNT.lower.int.ST1.C, rev(MNT.upper.int.ST1.C)), col = alpha("#482576FF", alpha = 0.5), border = NA)
```

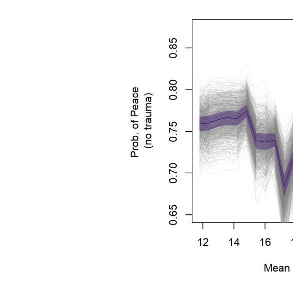

Supplemental Figure 25. Partial response of the probability of peace to variation in mean temperature on the coast while all other predictor variables' effects are held constant.

SST

```r
SST.list.C <- list(pdp_partials.C$PDP.SST.C.SST)
SST.resp.ST1.C <- aggregate(1-pdp_partials.C$PDP.SST.C.yhat, by = SST.list.C, FUN = mean)
SST.resp.ST1.sdE.C <- aggregate(1-pdp_partials.C$PDP.SST.C.yhat, by = SST.list.C, FUN = sd)
SST.margin.ST1.C <- qt(0.975, df = length(SST.resp.ST1.C$Group.1)-1) * SST.resp.ST1.sdE.C$x/sqrt(length(SST.resp.ST1.C$Group.1))#Calculate 95% CIs
SST.lower.int.ST1.C <- SST.resp.ST1.C$x - SST.margin.ST1.C#get the lower 95% CI boundary
SST.upper.int.ST1.C <- SST.resp.ST1.C$x + SST.margin.ST1.C#get the upper 95% CI boundary

par(pty = "s")
plot(SST.resp.ST1.C$x~SST.resp.ST1.C$Group.1, type = "l", ylab = "Prob. of Peace \n(no trauma)", xlab = "SST", ylim = c(0.65, 0.875), col = "#1E9C89FF")
for (i in 1:length(from.seq.plot)) {
  lines(1-pdp_partials.C$PDP.SST.C.yhat[from.seq.plot[i]:to.seq.plot[i]]~pdp_partials.C$PDP.SST.C.SST[1:21], col = alpha("grey50", alpha = 0.15), lwd = 0.5)
}
lines(SST.resp.ST1.C$x~SST.resp.ST1.C$Group.1, col = "#1E9C89FF")
polygon(x = c(SST.resp.ST1.C$Group.1, rev(SST.resp.ST1.C$Group.1)), y = c(SST.lower.int.ST1.C, rev(SST.upper.int.ST1.C)), col = alpha("#1E9C89FF", alpha = 0.5), border = NA)
```

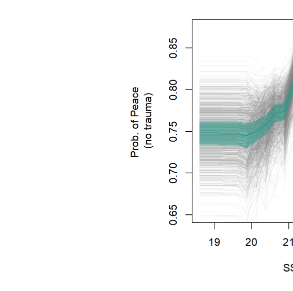

Supplemental Figure 26. Partial response of the probability of peace to variation in sea surface temperature on the coast while all other predictor variables' effects are held constant.

Population

```
POP.list.C <- list(pdp_partials.C$PDP.POP.C.pop.KDE)
POP.resp.ST1.C <- aggregate(1-pdp_partials.C$PDP.POP.C.yhat, by = POP.list.C, FUN = mean)
POP.resp.ST1.sdE.C <- aggregate(1-pdp_partials.C$PDP.POP.C.yhat, by = POP.list.C, FUN = sd)
POP.margin.ST1.C <- qt(0.975, df = length(POP.resp.ST1.C$Group.1)-1) * POP.resp.ST1.sdE.C$x/sqrt(length(POP.resp.
ST1.C$Group.1))#Calculate 95% CIs
POP.lower.int.ST1.C <- POP.resp.ST1.C$x - POP.margin.ST1.C#get the lower 95% CI boundary
POP.upper.int.ST1.C <- POP.resp.ST1.C$x + POP.margin.ST1.C#get the upper 95% CI boundary

par(pty = "s")
plot(POP.resp.ST1.C$x~POP.resp.ST1.C$Group.1, type = "l", ylab = "Prob. of Peace \n(no trauma)", xlab = "Populati
on Size", ylim = c(0.65, 0.875), col = "#FDE725FF")
for (i in 1:length(from.seq.plot)) {
  lines(1-pdp_partials.C$PDP.POP.C.yhat[from.seq.plot[i]:to.seq.plot[i]]~pdp_partials.C$PDP.POP.C.pop.KDE[1:21],
col = alpha("grey50", alpha =  0.15), lwd = 0.5)
}
lines(POP.resp.ST1.C$x~POP.resp.ST1.C$Group.1, col = "#FDE725FF")
polygon(x = c(POP.resp.ST1.C$Group.1, rev(POP.resp.ST1.C$Group.1)), y = c(POP.lower.int.ST1.C, rev(POP.upper.int.
ST1.C)), col = alpha("#FDE725FF", alpha = 0.5), border = NA)
```

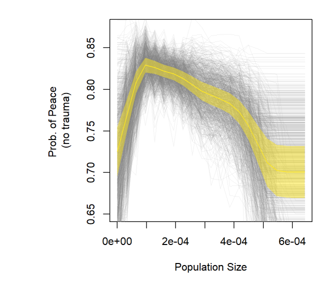

Supplemental Figure 27. Partial response of the probability of peace to variation in relative population size on the coast while all other predictor variables' effects are held constant.

ENSO

```
ENS.list.C <- list(pdp_partials.C$PDP.ENS.C.ENSO)
ENS.resp.ST1.C <- aggregate(1-pdp_partials.C$PDP.ENS.C.yhat, by = ENS.list.C, FUN = mean)
ENS.resp.ST1.sdE.C <- aggregate(1-pdp_partials.C$PDP.ENS.C.yhat, by = ENS.list.C, FUN = sd)
ENS.margin.ST1.C <- qt(0.975, df = length(ENS.resp.ST1.C$Group.1)-1) * ENS.resp.ST1.sdE.C$x/sqrt(length(ENS.resp.
ST1.C$Group.1))#Calculate 95% CIs
ENS.lower.int.ST1.C <- ENS.resp.ST1.C$x - ENS.margin.ST1.C#get the lower 95% CI boundary
ENS.upper.int.ST1.C <- ENS.resp.ST1.C$x + ENS.margin.ST1.C#get the upper 95% CI boundary

par(pty = "s")
plot(ENS.resp.ST1.C$x~ENS.resp.ST1.C$Group.1, type = "l", ylab = "Prob. of Peace \n(no trauma)", xlab = "ENSO Fre
q", ylim = c(0.65, 0.875), col = "#6BCD5AFF")
for (i in 1:length(from.seq.plot)) {
  lines(1-pdp_partials.C$PDP.ENS.C.yhat[from.seq.plot[i]:to.seq.plot[i]]~pdp_partials.C$PDP.ENS.C.ENSO[1:21], col
= alpha("grey50", alpha = 0.15), lwd = 0.5)
}
lines(ENS.resp.ST1.C$x~ENS.resp.ST1.C$Group.1, col = "#6BCD5AFF")
polygon(x = c(ENS.resp.ST1.C$Group.1, rev(ENS.resp.ST1.C$Group.1)), y = c(ENS.lower.int.ST1.C, rev(ENS.upper.int.
ST1.C)), col = alpha("#6BCD5AFF", alpha = 0.5), border = NA)
```

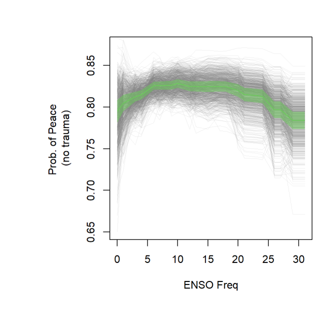

Supplemental Figure 28. Partial response of the probability of peace to variation in ENSO frequency on the coast while all other predictor variables' effects are held constant.

State

```
State.list.C <- list(pdp_partials.C$PDP.STT.C.Individuals_state)
State.resp.ST1.C <- aggregate(1-pdp_partials.C$PDP.STT.C.yhat, by = State.list.C, FUN = mean)
State.resp.ST1.sdE.C <- aggregate(1-pdp_partials.C$PDP.STT.C.yhat, by = State.list.C, FUN = sd)
State.margin.ST1.C <- qt(0.975, df = length(State.resp.ST1.C$Group.1)-1) * State.resp.ST1.sdE.C$x/sqrt(length(Sta
te.resp.ST1.C$Group.1))#Calculate 95% CIs
State.lower.int.ST1.C <- State.resp.ST1.C$x - State.margin.ST1.C#get the lower 95% CI boundary
State.upper.int.ST1.C <- State.resp.ST1.C$x + State.margin.ST1.C#get the upper 95% CI boundary

par(pty = "s")
plot(y = State.resp.ST1.C$x, x = as.numeric(State.resp.ST1.C$Group.1), pch = 19, col = c("black","black","blac
k"), ylab = "Prob. of Peace \n(no trauma)", xlab = "State Presence", ylim = c(0.65, 0.875), xaxt = "n")
for (i in 1:length(from.seq.plot)) {
  lines(1-pdp_partials.C$PDP.STT.C.yhat[from.seq.plot[i]:to.seq.plot[i]]~pdp_partials.C$PDP.STT.C.Individuals_sta
te[1:21], col = alpha("grey50", alpha = 0.15), lwd = 0.5)
}
lines(State.resp.ST1.C$x~State.resp.ST1.C$Group.1, col = alpha("black", alpha = 0.5), lwd = 2)
points(y = State.resp.ST1.C$x, x = as.numeric(State.resp.ST1.C$Group.1), pch = 19, col = c("black","black","blac
k"), cex = 1.5)
axis(side = 1, at = c(1,2,3), labels = c(0,1,2), tick = TRUE)
```

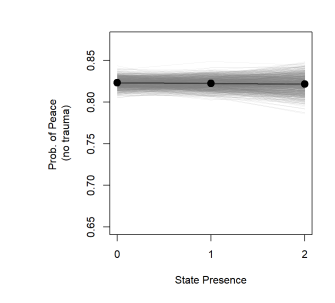

Supplemental Figure 29. Partial response of the probability of peace to variation in sociopolitical complexity on the coast while all other predictor variables' effects are held constant.

Now make a full plot

```
par(mfrow = c(2,3))
par(pty = "s")
plot(MNT.resp.ST1.C$x~MNT.resp.ST1.C$Group.1, type = "l", ylab = "Prob. of Peace \n(no trauma)", xlab = "Mean Tem
p.", ylim = c(0.65, 0.875), col = "#482576FF")
for (i in 1:length(from.seq.plot)) {
  lines(1-pdp_partials.C$PDP.MNT.C.yhat[from.seq.plot[i]:to.seq.plot[i]]~pdp_partials.C$PDP.MNT.C.MNtemp20km[1:2
1], col = alpha("grey50", alpha = 0.15), lwd = 0.5)
}

lines(MNT.resp.ST1.C$x~MNT.resp.ST1.C$Group.1, col = "#482576FF")
polygon(x = c(MNT.resp.ST1.C$Group.1, rev(MNT.resp.ST1.C$Group.1)), y = c(MNT.lower.int.ST1.C, rev(MNT.upper.int.
ST1.C)), col = alpha("#482576FF", alpha = 0.5), border = NA)

plot(MNP.resp.ST1.C$x~MNP.resp.ST1.C$Group.1, type = "l", ylab = "", yaxt = "n", xlab = "Mean Precip.", ylim = c(
0.65, 0.875), col = "#31668EFF")
for (i in 1:length(from.seq.plot)) {
  lines(1-pdp_partials.C$PDP.MNP.C.yhat[from.seq.plot[i]:to.seq.plot[i]]~pdp_partials.C$PDP.MNP.C.MNprcp20km[1:2
1], col = alpha("grey50", alpha = 0.15), lwd = 0.5)
}

lines(MNP.resp.ST1.C$x~MNP.resp.ST1.C$Group.1, col = "#31668EFF")
polygon(x = c(MNP.resp.ST1.C$Group.1, rev(MNP.resp.ST1.C$Group.1)), y = c(MNP.lower.int.ST1.C, rev(MNP.upper.int.
ST1.C)), col = alpha("#31668EFF", alpha = 0.5), border = NA)

par(pty = "s")
plot(ENS.resp.ST1.C$x~ENS.resp.ST1.C$Group.1, type = "l", ylab = "", yaxt = "n", xlab = "ENSO Freq.", ylim = c(0.
65, 0.875), col = "#6BCD5AFF")
for (i in 1:length(from.seq.plot)) {
  lines(1-pdp_partials.C$PDP.ENS.C.yhat[from.seq.plot[i]:to.seq.plot[i]]~pdp_partials.C$PDP.ENS.C.ENSO[1:21], col
= alpha("grey50", alpha = 0.15), lwd = 0.5)
}

lines(ENS.resp.ST1.C$x~ENS.resp.ST1.C$Group.1, col = "#6BCD5AFF")
polygon(x = c(ENS.resp.ST1.C$Group.1, rev(ENS.resp.ST1.C$Group.1)), y = c(ENS.lower.int.ST1.C, rev(ENS.upper.int.
ST1.C)), col = alpha("#6BCD5AFF", alpha = 0.5), border = NA)

par(pty = "s")
plot(y = State.resp.ST1.C$x, x = as.numeric(State.resp.ST1.C$Group.1), pch = 19, col = "black", ylab = "Prob. of
Peace \n(no trauma)", xlab = "State Presence", ylim = c(0.65, 0.875), xaxt = "n")
for (i in 1:length(from.seq.plot)) {
  lines(1-pdp_partials.C$PDP.STT.C.yhat[from.seq.plot[i]:to.seq.plot[i]]~pdp_partials.C$PDP.STT.C.Individuals_sta
te[1:21], col = alpha("grey50", alpha = 0.15), lwd = 0.5)
}
lines(State.resp.ST1.C$x~State.resp.ST1.C$Group.1, col = alpha("black", alpha = 0.5), lwd = 2)
points(y = State.resp.ST1.C$x, x = as.numeric(State.resp.ST1.C$Group.1), pch = 19, col = "black", cex = 1.5)
axis(side = 1, at = c(1,2,3), labels = c(0,1,2), tick = TRUE)

par(pty = "s")
plot(POP.resp.ST1.C$x~POP.resp.ST1.C$Group.1, type = "l", ylab = "", yaxt = "n", xlab = "Population (KDE)", ylim
= c(0.65, 0.875), col = "#FDE725FF")
for (i in 1:length(from.seq.plot)) {
  lines(1-pdp_partials.C$PDP.POP.C.yhat[from.seq.plot[i]:to.seq.plot[i]]~pdp_partials.C$PDP.POP.C.pop.KDE[1:21],
col = alpha("grey50", alpha = 0.15), lwd = 0.5)
}

lines(POP.resp.ST1.C$x~POP.resp.ST1.C$Group.1, col = "#FDE725FF")
polygon(x = c(POP.resp.ST1.C$Group.1, rev(POP.resp.ST1.C$Group.1)), y = c(POP.lower.int.ST1.C, rev(POP.upper.int.
ST1.C)), col = alpha("#FDE725FF", alpha = 0.5), border = NA)

par(pty = "s")
plot(SST.resp.ST1.C$x~SST.resp.ST1.C$Group.1, type = "l", ylab = "", yaxt = "n", xlab = "SST", ylim = c(0.65, 0.8
75), col = "#1E9C89FF")
for (i in 1:length(from.seq.plot)) {
  lines(1-pdp_partials.C$PDP.SST.C.yhat[from.seq.plot[i]:to.seq.plot[i]]~pdp_partials.C$PDP.SST.C.SST[1:21], col
= alpha("grey50", alpha = 0.15), lwd = 0.5)
}

lines(SST.resp.ST1.C$x~SST.resp.ST1.C$Group.1, col = "#1E9C89FF")
polygon(x = c(SST.resp.ST1.C$Group.1, rev(SST.resp.ST1.C$Group.1)), y = c(SST.lower.int.ST1.C, rev(SST.upper.int.
ST1.C)), col = alpha("#1E9C89FF", alpha = 0.5), border = NA)
```

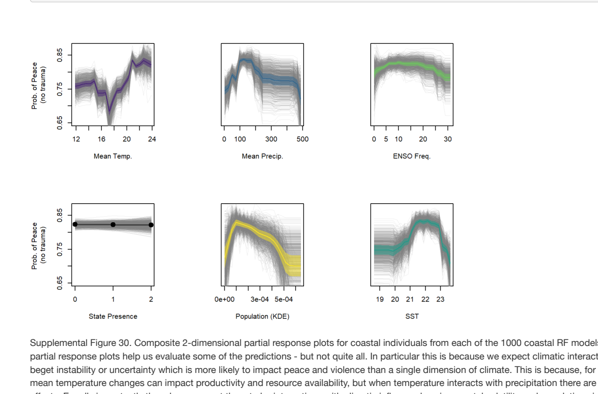

Supplemental Figure 30. Composite 2-dimensional partial response plots for coastal individuals from each of the 1000 coastal RF models. These partial response plots help us evaluate some of the predictions - but not quite all. In particular this is because we expect climatic interactions to beget instability or uncertainty which is more likely to impact peace and violence than a single dimension of climate. This is because, for example, mean temperature changes can impact productivity and resource availability, but when temperature interacts with precipitation there are greater effects. Equally importantly though, we expect there to be interactions with climatic influenced environmental volatility and population size.

To investigate these interactions, we use 3d partial response plots from a sub-sample of 100 models. We use 100 models because it takes >25 minutes to generate these 3d partial response data for a single model and the computational time for all 1000 would be >420 hours.

To visualize the 3d partial responses, we aggregate the data from the 100 models. We focus these interaction plots on the impact of climate interacting with population density, which we explore given the strong Allee effects apparent in the 2d partial plot for population density. We also provide an interaction plot for mean precipitation with mean temperature to further capture volatility.

Mean Temp.

```
MNT.3d.p <- aggregate(pdp3d_partials.C$MNT.KDE.PDP.C.yhat~pdp3d_partials.C$MNT.KDE.PDP.C.MNtemp20km+pdp3d_partial
s.C$MNT.KDE.PDP.C.pop.KDE, FUN = mean)
colnames(MNT.3d.p) <- c("MNtemp20km", "pop.KDE", "yhat")

C.MNT.3d <- ggplot() +
  theme(aspect.ratio = 1, panel.background = element_blank()) + #make the plot square
  geom_raster(data = MNT.3d.p, aes(x = MNtemp20km, y = pop.KDE, fill = 1-yhat)) +
  scale_fill_viridis_b(n.breaks = 10, breaks = seq(0.625,0.85,0.025), limits = c(0.625,0.85), option = "turbo", d
irection = -1, labels = c("0.625", "", "", "0.700", "", " ", "0.775", " ", "", "0.850")) +
  xlab("Mean Temp. (C)") +
  ylab("Pop. Density (KDE)") +
  labs(fill = "Prob. of Peace \n(no trauma)") +
  scale_x_continuous(breaks = round(seq(min(MNT.3d.p$MNtemp20km), max(MNT.3d.p$MNtemp20km), len = 7))) +
  scale_y_continuous(breaks = round(seq(min(MNT.3d.p$pop.KDE), max(MNT.3d.p$pop.KDE), len = 7),4)) +
  geom_vline(xintercept = mean(pdp3d_partials.C$MNT.KDE.PDP.C.MNtemp20km), col = "grey95", size = 1.5) +
  geom_hline(yintercept = mean(pdp3d_partials.C$MNT.KDE.PDP.C.pop.KDE), col = "grey95", size = 1.5)

#ggsave(filename = "C.MNT.3d.png", plot = C.MNT.3d, device = "png", height = 5, width = 5, units = "in", dpi = 30
0)
#ggsave(filename = "C.MNT.3d.pdf", plot = C.MNT.3d, device = "pdf", height = 5, width = 5, units = "in", dpi = 30
0)

C.MNT.3d
```

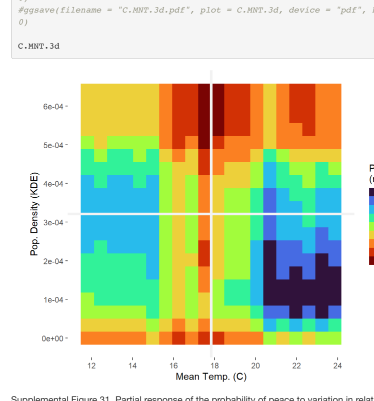

Supplemental Figure 31. Partial response of the probability of peace to variation in relative population density and mean temperature on the coast while all other predictor variables' effects are held constant.

Mean Precip.

```
MNP.3d.p <- aggregate(pdp3d_partials.C$MNP.KDE.PDP.C.yhat~pdp3d_partials.C$MNP.KDE.PDP.C.MNprcp20km+pdp3d_partial
s.C$MNP.KDE.PDP.C.pop.KDE, FUN = mean)
colnames(MNP.3d.p) <- c("MNprcp20km", "pop.KDE", "yhat")

C.MNP.3d <- ggplot() +
  theme(aspect.ratio = 1, panel.background = element_blank()) + #make the plot square
  geom_raster(data = MNP.3d.p, aes(x = MNprcp20km, y = pop.KDE, fill = 1-yhat)) +
  scale_fill_viridis_b(n.breaks = 10, breaks = seq(0.625,0.85,0.025), limits = c(0.625,0.85), option = "turbo", d
irection = -1, labels = c("0.625", "", "", "0.700", "", " ", "0.775", " ", "", "0.850")) +
  xlab("Mean Precip. (mm/yr)") +
  ylab("Pop. Density (KDE)") +
  labs(fill = "Prob. of Peace \n(no trauma)") +
  scale_x_continuous(breaks = round(seq(min(MNP.3d.p$MNprcp20km), max(MNP.3d.p$MNprcp20km), len = 7))) +
  scale_y_continuous(breaks = round(seq(min(MNP.3d.p$pop.KDE), max(MNP.3d.p$pop.KDE), len = 7),4)) +
  geom_vline(xintercept = mean(pdp3d_partials.C$MNP.KDE.PDP.C.MNprcp20km), col = "grey95", size = 1.5) +
  geom_hline(yintercept = mean(pdp3d_partials.C$MNP.KDE.PDP.C.pop.KDE), col = "grey95", size = 1.5)

#ggsave(filename = "C.MNP.3d.png", plot = C.MNP.3d, device = "png", height = 5, width = 5, units = "in", dpi = 30
0)
#ggsave(filename = "C.MNP.3d.pdf", plot = C.MNP.3d, device = "pdf", height = 5, width = 5, units = "in", dpi = 30
0)

C.MNP.3d
```

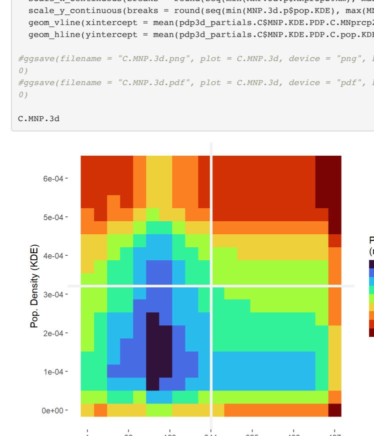

Supplemental Figure 32. Partial response of the probability of peace to variation in relative population density and mean precipitation on the coast while all other predictor variables' effects are held constant.

SST

```
SST.3d.p <- aggregate(pdp3d_partials.C$SST.KDE.PDP.C.yhat~pdp3d_partials.C$SST.KDE.PDP.C.SST+pdp3d_partials.C$SS
T.KDE.PDP.C.pop.KDE, FUN = mean)
colnames(SST.3d.p) <- c("SST", "pop.KDE", "yhat")
SST.3d.p$yhat[which(SST.3d.p$yhat > 1.0)] <- 1.0 #there is 1 prediction that is 1.0003 or so, so for plotting pur
poses we will call that 1

C.SST.3d <- ggplot() +
  theme(aspect.ratio = 1, panel.background = element_blank()) + #make the plot square
  geom_raster(data = SST.3d.p, aes(x = SST, y = pop.KDE, fill = 1-yhat)) +
  scale_fill_viridis_b(n.breaks = 10, breaks = seq(0.625,0.85,0.025), limits = c(0.625,0.85), option = "turbo", d
irection = -1, labels = c("0.625", "", "", "0.700", "", " ", "0.775", " ", "", "0.850")) +
  xlab("SST (C)") +
  ylab("Pop. Density (KDE)") +
  labs(fill = "Prob. of Peace \n(no trauma)") +
  scale_x_continuous(breaks = round(seq(min(SST.3d.p$SST), max(SST.3d.p$SST), len = 7),2)) +
  scale_y_continuous(breaks = round(seq(min(SST.3d.p$pop.KDE), max(SST.3d.p$pop.KDE), len = 7),4)) +
  geom_vline(xintercept = mean(pdp3d_partials.C$SST.KDE.PDP.C.SST), col = "grey95", size = 1.5) +
  geom_hline(yintercept = mean(pdp3d_partials.C$SST.KDE.PDP.C.pop.KDE), col = "grey95", size = 1.5)

#ggsave(filename = "C.SST.3d.png", plot = C.SST.3d, device = "png", height = 5, width = 5, units = "in", dpi = 30
0)
#ggsave(filename = "C.SST.3d.pdf", plot = C.SST.3d, device = "pdf", height = 5, width = 5, units = "in", dpi = 30
0)

C.SST.3d
```

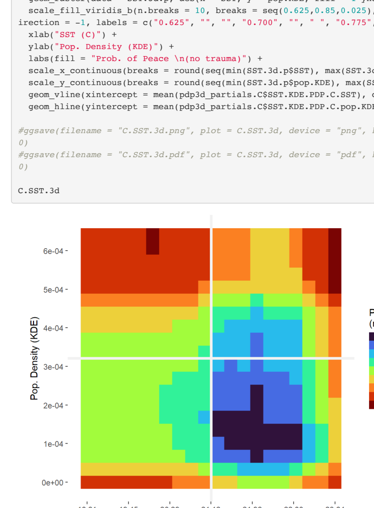

Supplemental Figure 33. Partial response of the probability of peace to variation in relative population density and sea surface temperature on the coast while all other predictor variables' effects are held constant.

ENSO

```
ENS.3d.p <- aggregate(pdp3d_partials.C$ENS.KDE.PDP.C.yhat~pdp3d_partials.C$ENS.KDE.PDP.C.ENSO+pdp3d_partials.C$EN
S.KDE.PDP.C.pop.KDE, FUN = mean)
colnames(ENS.3d.p) <- c("ENSO", "pop.KDE", "yhat")

#The gaps in ENSO values are not even (for example, we go from 0 to 1 and then 1 to 3). This causes an issue with
plotting because we don't have continuous variables. We can address this by generating an estimate for the missin
g variables - which we do below.

#use tidyverse for add_row
library(tidyverse)

   st <- seq(1,400,20) #start row sequence
   en <- seq(20,400,20) #end row sequence
   pop.kde.seq <- unique(ENS.3d.p$pop.KDE)#sequence of pop values from our partial predictions

   ENS.POP.3d <- data.frame()#empty dataframe to hold ouptut

for (i in 1:20) { #for our 20 ENSO / pop.KDE combinations
     test.df <- ENS.3d.p[st[i]:en[i],] #subset to just 1 combination
     test.df <- test.df %>% #add the missing ENSO observation values as rows
       add_row(ENSO = c(2,5,7,10,12,15,18,20,23,25,28,30),
             pop.KDE = pop.kde.seq[i], #insert the correct pop.KDE value from the sequence
             yhat = NA, #make yhat (prob no trauma) NA - we will fill in later
             .before = 3) #place the new rows before row 3

     test.df <- arrange(test.df, by = test.df$ENSO) #order the df by ENSO in ascending order
     test.df$yhat <- approx(test.df$yhat, n = 32)$y #use the approx function to estimate the missing yhat values
(just uses the prior and succeeding observation to each missing one - think of it like an average of the two)
     ENS.POP.3d <- rbind(ENS.POP.3d, test.df)#put the new rows into a dataframe
     }

#This makes the plot before our filling in the dataframe just to see what it looks like and to compare
#ggplot() +
#  theme(aspect.ratio = 1, panel.background = element_blank()) + #make the plot square
#  geom_raster(data = ENS.3d.p, aes(x = ENSO, y = pop.KDE, fill = yhat)) +
#  scale_fill_viridis_b(n.breaks = 10, limits = c(0,1), option = "turbo", direction = -1) +
#  xlab("ENSO Freq. / Century") +
#  ylab("Pop. Density (KDE)") +
#  labs(fill = "Prob. No Trauma") +
#  scale_x_continuous(breaks = round(seq(min(ENS.3d.p$ENSO), max(ENS.3d.p$ENSO), len = 7))) +
#  scale_y_continuous(breaks = round(seq(min(ENS.3d.p$pop.KDE), max(ENS.3d.p$pop.KDE), len = 7),4))

#this makes the plot with the filled in data we did above so there aren't odd white gaps in the plot
C.ENS.3d <- ggplot() +
  theme(aspect.ratio = 1, panel.background = element_blank()) + #make the plot square
  geom_raster(data = ENS.POP.3d, aes(x = ENSO, y = pop.KDE, fill = 1-yhat)) +
  scale_fill_viridis_b(n.breaks = 10, breaks = seq(0.625,0.85,0.025), limits = c(0.625,0.85), option = "turbo", d
irection = -1, labels = c("0.625", "", "", "0.700", "", " ", "0.775", " ", "", "0.850")) +
  xlab("ENSO Freq. / Century") +
  ylab("Pop. Density (KDE)") +
  labs(fill = "Prob. of Peace \n(no trauma)") +
  scale_x_continuous(breaks = round(seq(min(ENS.POP.3d$ENSO), max(ENS.POP.3d$ENSO), len = 7))) +
  scale_y_continuous(breaks = round(seq(min(ENS.POP.3d$pop.KDE), max(ENS.POP.3d$pop.KDE), len = 7),4)) +
  geom_vline(xintercept = 15.5, col = "grey95", size = 1.5) +
  geom_hline(yintercept = mean(pdp3d_partials.C$ENS.KDE.PDP.C.pop.KDE), col = "grey95", size = 1.5)

#ggsave(filename = "C.ENS.3d.png", plot = C.ENS.3d, device = "png", height = 5, width = 5, units = "in", dpi = 30
0)
#ggsave(filename = "C.ENS.3d.pdf", plot = C.ENS.3d, device = "pdf", height = 5, width = 5, units = "in", dpi = 30
0)

C.ENS.3d
```

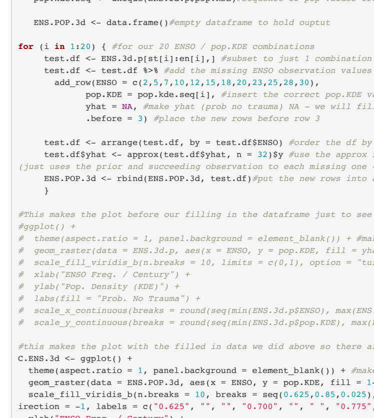

Supplemental Figure 34. Partial response of the probability of peace to variation in relative population density and ENSO frequency per century on the coast while all other predictor variables' effects are held constant.

Mean Temp and Mean Precip.

```
MNT.MNP.3d.p <- aggregate(pdp3d_partials.C$MNP.MNT.PDP.C.yhat~pdp3d_partials.C$MNP.MNT.PDP.C.MNtemp20km+pdp3d_par
tials.C$MNP.MNT.PDP.C.MNprcp20km, FUN = mean)
colnames(MNT.MNP.3d.p) <- c("MNtemp20km", "MNprcp20km", "yhat")
MNT.MNP.3d.p$yhat[which(MNT.MNP.3d.p$yhat > 1.0)] <- 1.0

library(ggplot2)
C.MNT.MNP.3d <- ggplot() +
  theme(aspect.ratio = 1, panel.background = element_blank()) + #make the plot square
  geom_raster(data = MNT.MNP.3d.p, aes(x = MNtemp20km, y = MNprcp20km, fill = 1-yhat)) +
  scale_fill_viridis_b(n.breaks = 10, breaks = seq(0.625,0.85,0.025), limits = c(0.625,0.85), option = "turbo", d
irection = -1, labels = c("0.625", "", "", "0.700", "", " ", "0.775", " ", "", "0.850")) +
  xlab("Mean Temp. (C)") +
  ylab("Mean Precip. (mm/year)") +
  labs(fill = "Prob. of Peace \n(no trauma)") +
  scale_x_continuous(breaks = round(seq(min(MNT.MNP.3d.p$MNtemp20km), max(MNT.MNP.3d.p$MNtemp20km), len = 7))) +
  scale_y_continuous(breaks = round(seq(min(MNT.MNP.3d.p$MNprcp20km), max(MNT.MNP.3d.p$MNprcp20km), len = 7),4))
+
  geom_vline(xintercept = mean(pdp3d_partials.C$MNT.KDE.PDP.C.MNtemp20km), col = "grey95", size = 1.5) +
  geom_hline(yintercept = mean(pdp3d_partials.C$MNP.KDE.PDP.C.MNprcp20km), col = "grey95", size = 1.5)

#ggsave(filename = "C.MNT.MNP.3d.png", plot = C.MNT.MNP.3d, device = "png", height = 5, width = 5, units = "in",
dpi = 300)
#ggsave(filename = "C.MNT.MNP.3d.pdf", plot = C.MNT.MNP.3d, device = "pdf", height = 5, width = 5, units = "in",
dpi = 300)

C.MNT.MNP.3d
```

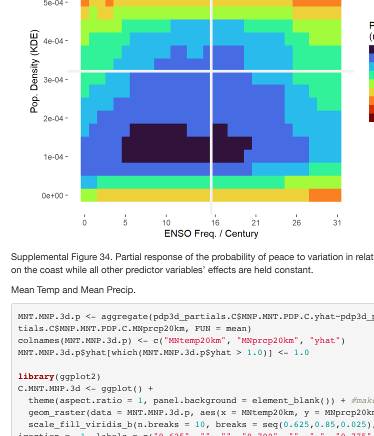

Supplemental Figure 35. Partial response of the probability of peace to variation in relative population density and mean temperature for non-coastal individuals while all other predictor variables' effects are held constant.

Non-Coastal Model Outcomes

Next we look at variable importance on the coast. This is calculated as the permutation variable importance.

```
MNT_Imp <- mean(all_performance.NC$MNT_Imp)#3rd
MNT_t <- t.test(all_performance.NC$MNT_Imp)

MNP_Imp <- mean(all_performance.NC$MNP_Imp)#1st
MNP_t <- t.test(all_performance.NC$MNP_Imp)

POP_Imp <- mean(all_performance.NC$POP_Imp)#4th
POP_t <- t.test(all_performance.NC$POP_Imp)

SDT_Imp <- mean(all_performance.NC$SDT_Imp)#2nd
SDT_t <- t.test(all_performance.NC$SDT_Imp)

ENS_Imp <- mean(all_performance.NC$ENS_Imp)#5th
ENS_t <- t.test(all_performance.NC$ENS_Imp)

STT_Imp <- mean(all_performance.NC$STT_Imp)#6th
STT_t <- t.test(all_performance.NC$STT_Imp)

#png(filename = "NonCoastal.VarImp.png", height = 5, width = 5, units = "in", res = 300)
#pdf(file = "NonCoastal.VarImp.pdf", height = 5, width = 5)
par(pty = "s", oma = c(2,2,0,0))
plot(x = MNT_Imp, y = 4, xlim = c(0, 0.05), ylim = c(1,6), yaxt = "n", ylab = "", pch = 19, xlab = "", col = "#48
2576FF", cex = 1.5)
lines(x = c(-0.90, 0.0505), y = c(6,6), col = alpha("grey80", alpha = 0.5), lty = 2)
lines(x = c(-0.90, 0.0505), y = c(5,5), col = alpha("grey80", alpha = 0.5), lty = 2)
lines(x = c(-0.90, 0.0505), y = c(4,4), col = alpha("grey80", alpha = 0.5), lty = 2)
lines(x = c(-0.90, 0.0505), y = c(3,3), col = alpha("grey80", alpha = 0.5), lty = 2)
lines(x = c(-0.90, 0.0505), y = c(2,2), col = alpha("grey80", alpha = 0.5), lty = 2)
lines(x = c(-0.90, 0.0505), y = c(1,1), col = alpha("grey80", alpha = 0.5), lty = 2)

lines(x = c(0, 0), y = c(0.9,6.10), col = alpha("grey80", alpha = 0.5), lty = 2)
lines(x = c(0.01, 0.01), y = c(0.9,6.10), col = alpha("grey80", alpha = 0.5), lty = 2)
lines(x = c(0.02, 0.02), y = c(0.9,6.10), col = alpha("grey80", alpha = 0.5), lty = 2)
lines(x = c(0.03, 0.03), y = c(0.9,6.10), col = alpha("grey80", alpha = 0.5), lty = 2)
lines(x = c(0.04, 0.04), y = c(0.9,6.10), col = alpha("grey80", alpha = 0.5), lty = 2)
lines(x = c(0.05, 0.05), y = c(0.9,6.10), col = alpha("grey80", alpha = 0.5), lty = 2)
lines(x = c(mean(all_performance.NC$MNP_Imp) + sd(all_performance.NC$MNP_Imp)*2, mean(all_performance.NC$MNP_Imp)
- sd(all_performance.NC$MNP_Imp)*2), y = c(6,6), col = "black")
lines(x = c(mean(all_performance.NC$SDT_Imp) + sd(all_performance.NC$SDT_Imp)*2, mean(all_performance.NC$SDT_Imp)
- sd(all_performance.NC$SDT_Imp)*2), y = c(5,5), col = "black")
lines(x = c(mean(all_performance.NC$MNT_Imp) + sd(all_performance.NC$MNT_Imp)*2, mean(all_performance.NC$MNT_Imp)
- sd(all_performance.NC$MNT_Imp)*2), y = c(4,4), col = "black")
lines(x = c(mean(all_performance.NC$POP_Imp) + sd(all_performance.NC$POP_Imp)*2, mean(all_performance.NC$POP_Imp)
- sd(all_performance.NC$POP_Imp)*2), y = c(3,3), col = "black")
lines(x = c(mean(all_performance.NC$ENS_Imp) + sd(all_performance.NC$ENS_Imp)*2, mean(all_performance.NC$ENS_Imp)
- sd(all_performance.NC$ENS_Imp)*2), y = c(2,2), col = "black")
lines(x = c(mean(all_performance.NC$STT_Imp) + sd(all_performance.NC$STT_Imp)*2, mean(all_performance.NC$STT_Imp)
- sd(all_performance.NC$STT_Imp)*2), y = c(1,1), col = "black")
points(x = MNT_Imp, y = 4, pch = 19, cex = 1.5, col = "#482576FF")
lines(x = c(MNT_t$conf.int[1], MNT_t$conf.int[2]), y = c(4,4), col = "white", lwd = 1)
points(x = MNP_Imp, y = 6, pch = 19, cex = 1.5, col = "#31668EFF")
lines(x = c(MNP_t$conf.int[1], MNP_t$conf.int[2]), y = c(6,6), col = "white")
points(x = ENS_Imp, y = 2, pch = 19, cex = 1.5, col = "#6BCD5AFF")
lines(x = c(ENS_t$conf.int[1], ENS_t$conf.int[2]), y = c(2,2), col = "white")
points(x = STT_Imp, y = 1, pch = 19, cex = 1.5, col = "black")
lines(x = c(STT_t$conf.int[1], STT_t$conf.int[2]), y = c(1,1), col = "white")
points(x = POP_Imp, y = 3, pch = 19, cex = 1.5, col = "#FDE725FF")
lines(x = c(POP_t$conf.int[1], POP_t$conf.int[2]), y = c(3,3), col = "white")
points(x = SDT_Imp, y = 5, pch = 19, cex = 1.5, col = "#1E9C89FF")
lines(x = c(SDT_t$conf.int[1], SDT_t$conf.int[2]), y = c(5,5), col = "white")
axis(2, at = c(1,2,3,4,5,6), labels = FALSE, tick = TRUE)
mtext("Mean Temp.", at = 4, side = 2, line = 1, las = 1)
mtext("Mean Precip.", at = 6, side = 2, line = 1, las = 1)
mtext("ENSO Freq.", at = 2, side = 2, line = 1, las = 1)
mtext("State", at = 1, side = 2, line = 1, las = 1)
mtext("Population (KDE)", at = 3, side = 2, line = 1, las = 1)
mtext("Std Dev. Temp.", at = 5, side = 2, line = 1, las = 1)
mtext("Variable Importance", side = 1, line = 2)
```

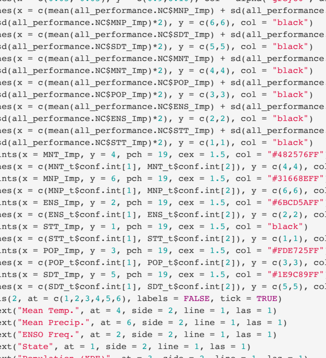

Supplemental Figure 36. Variable importance for non-coastal models. Colored dots provide the central tendency value for variable importance from the aggregate 1000 models, black lines are two standard deviations around the mean,and the white line provides the 95% confidence interval on the mean. Like coastal outputs, the variable importance suggests that climate is more important than demographic and social variables in predicting the presence/absence of trauma. To further evalaute predictions, we use 2 and 3 dimensional partial response plots.

Mean Precip

```
#Get the aggregated predicted responses and make info for a confidence interval for precip
MNP.list.NC <- list(pdp_partials.NC$PDP.MNP.NC.MNprcp20km)
MNP.resp.ST1.NC <- aggregate(1-pdp_partials.NC$PDP.MNP.NC.yhat, by = MNP.list.NC, FUN = mean)#Get the average pre
dicted probability no trauma for each precip value
MNP.resp.ST1.sdE.NC <- aggregate(1-pdp_partials.NC$PDP.MNP.NC.yhat, by = MNP.list.NC, FUN = sd)#Get the st dev in
predicted probability no trauma for each precip value
MNP.margin.ST1.NC <- qt(0.975, df = length(MNP.resp.ST1.NC$Group.1)-1) * MNP.resp.ST1.sdE.NC$x/sqrt(length(MNP.re
sp.ST1.NC$Group.1))#Calculate 95% CIs
MNP.lower.int.ST1.NC <- MNP.resp.ST1.NC$x - MNP.margin.ST1.NC#get the lower 95% CI boundary
MNP.upper.int.ST1.NC <- MNP.resp.ST1.NC$x + MNP.margin.ST1.NC#get the upper 95% CI boundary

from.seq.plot <- seq(1, 10500, 21)
to.seq.plot <- seq(21, 10500, 21)

par(pty = "s")
plot(MNP.resp.ST1.NC$x~MNP.resp.ST1.NC$Group.1, type = "l", ylab = "Prob. of Peace \n(no trauma)", xlab = "Mean P
recip.", ylim = c(0.6, 0.8), col = "#31668EFF")
for (i in 1:length(from.seq.plot)) {
  lines(1-pdp_partials.NC$PDP.MNP.NC.yhat[from.seq.plot[i]:to.seq.plot[i]]~pdp_partials.NC$PDP.MNP.NC.MNprcp20km[
1:21], col = alpha("grey50", alpha = 0.15), lwd = 0.5)
}
lines(MNP.resp.ST1.NC$x~MNP.resp.ST1.NC$Group.1, col = "#31668EFF")
polygon(x = c(MNP.resp.ST1.NC$Group.1, rev(MNP.resp.ST1.NC$Group.1)), y = c(MNP.lower.int.ST1.NC, rev(MNP.upper.i
nt.ST1.NC)), col = alpha("#31668EFF", alpha = 0.5), border = NA)
```

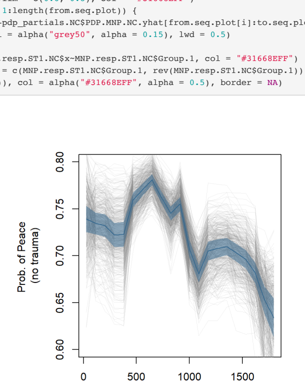

Supplemental Figure 37. Partial response of the probability of peace to variation in mean precipitation for non-coastal individuals while all other predictor variables' effects are held constant.

Mean Temp

```
MNT.list.NC <- list(pdp_partials.NC$PDP.MNT.NC.MNtemp20km)
MNT.resp.ST1.NC <- aggregate(1-pdp_partials.NC$PDP.MNT.NC.yhat, by = MNT.list.NC, FUN = mean)
MNT.resp.ST1.sdE.NC <- aggregate(1-pdp_partials.NC$PDP.MNT.NC.yhat, by = MNT.list.NC, FUN = sd)
MNT.margin.ST1.NC <- qt(0.975, df = length(MNT.resp.ST1.NC$Group.1)-1) * MNT.resp.ST1.sdE.NC$x/sqrt(length(MNT.re
sp.ST1.NC$Group.1))#Calculate 95% CIs
MNT.lower.int.ST1.NC <- MNT.resp.ST1.NC$x - MNT.margin.ST1.NC#get the lower 95% CI boundary
MNT.upper.int.ST1.NC <- MNT.resp.ST1.NC$x + MNT.margin.ST1.NC#get the upper 95% CI boundary

par(pty = "s")
plot(MNT.resp.ST1.NC$x~MNT.resp.ST1.NC$Group.1, type = "l", ylab = "Prob. of Peace \n(no trauma)", xlab = "Mean T
emp.", ylim = c(0.6, 0.8), col = "#482576FF")
for (i in 1:length(from.seq.plot)) {
  lines(1-pdp_partials.NC$PDP.MNT.NC.yhat[from.seq.plot[i]:to.seq.plot[i]]~pdp_partials.NC$PDP.MNT.NC.MNtemp20km[
1:21], col = alpha("grey50", alpha = 0.15), lwd = 0.5)
}
lines(MNT.resp.ST1.NC$x~MNT.resp.ST1.NC$Group.1, col = "#482576FF")
polygon(x = c(MNT.resp.ST1.NC$Group.1, rev(MNT.resp.ST1.NC$Group.1)), y = c(MNT.lower.int.ST1.NC, rev(MNT.upper.i
nt.ST1.NC)), col = alpha("#482576FF", alpha = 0.5), border = NA)
```

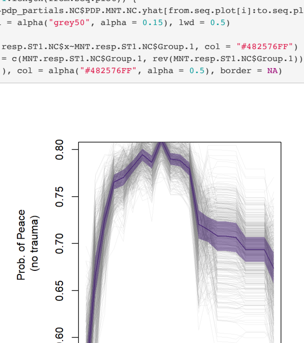

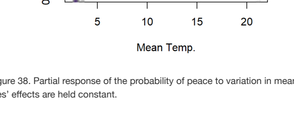

Supplemental Figure 38. Partial response of the probability of peace to variation in mean temperature for non-coastal individuals while all other predictor variables' effects are held constant.

SDT

```
SDT.list.NC <- list(pdp_partials.NC$PDP.SDT.NC.SDtemp20km)
SDT.resp.ST1.NC <- aggregate(1-pdp_partials.NC$PDP.SDT.NC.yhat, by = SDT.list.NC, FUN = mean)
SDT.resp.ST1.sdE.NC <- aggregate(1-pdp_partials.NC$PDP.SDT.NC.yhat, by = SDT.list.NC, FUN = sd)
SDT.margin.ST1.NC <- qt(0.975, df = length(SDT.resp.ST1.NC$Group.1)-1) * SDT.resp.ST1.sdE.NC$x/sqrt(length(SDT.re
sp.ST1.NC$Group.1))#Calculate 95% CIs
SDT.lower.int.ST1.NC <- SDT.resp.ST1.NC$x - SDT.margin.ST1.NC#get the lower 95% CI boundary
SDT.upper.int.ST1.NC <- SDT.resp.ST1.NC$x + SDT.margin.ST1.NC#get the upper 95% CI boundary

par(pty = "s")
plot(SDT.resp.ST1.NC$x~SDT.resp.ST1.NC$Group.1, type = "l", ylab = "Prob. of Peace \n(no trauma)", xlab = "SDT",
ylim = c(0.6, 0.8), col = "#1E9C89FF")
for (i in 1:length(from.seq.plot)) {
  lines(1-pdp_partials.NC$PDP.SDT.NC.yhat[from.seq.plot[i]:to.seq.plot[i]]~pdp_partials.NC$PDP.SDT.NC.SDtemp20km[
1:21], col = alpha("grey50", alpha = 0.15), lwd = 0.5)
}
lines(SDT.resp.ST1.NC$x~SDT.resp.ST1.NC$Group.1, col = "#1E9C89FF")
polygon(x = c(SDT.resp.ST1.NC$Group.1, rev(SDT.resp.ST1.NC$Group.1)), y = c(SDT.lower.int.ST1.NC, rev(SDT.upper.i
nt.ST1.NC)), col = alpha("#1E9C89FF", alpha = 0.5), border = NA)
```

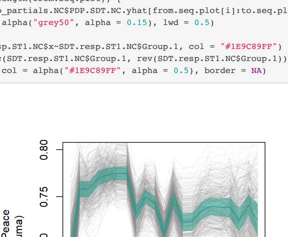

Supplemental Figure 39. Partial response of the probability of peace to variation in standard deviation in temperature for non-coastal individuals while all other predictor variables' effects are held constant.

Population

```
POP.list.NC <- list(pdp_partials.NC$PDP.POP.NC.pop.KDE)
POP.resp.ST1.NC <- aggregate(1-pdp_partials.NC$PDP.POP.NC.yhat, by = POP.list.NC, FUN = mean)
POP.resp.ST1.sdE.NC <- aggregate(1-pdp_partials.NC$PDP.POP.NC.yhat, by = POP.list.NC, FUN = sd)
POP.margin.ST1.NC <- qt(0.975, df = length(POP.resp.ST1.NC$Group.1)-1) * POP.resp.ST1.sdE.NC$x/sqrt(length(POP.re
sp.ST1.NC$Group.1))#Calculate 95% CIs
POP.lower.int.ST1.NC <- POP.resp.ST1.NC$x - POP.margin.ST1.NC#get the lower 95% CI boundary
POP.upper.int.ST1.NC <- POP.resp.ST1.NC$x + POP.margin.ST1.NC#get the upper 95% CI boundary

par(pty = "s")
plot(POP.resp.ST1.NC$x~POP.resp.ST1.NC$Group.1, type = "l", ylab = "Prob. of Peace \n(no trauma)", xlab = "Popula
tion Size", ylim = c(0.6, 0.8), col = "#FDE725FF")
for (i in 1:length(from.seq.plot)) {
  lines(1-pdp_partials.NC$PDP.POP.NC.yhat[from.seq.plot[i]:to.seq.plot[i]]~pdp_partials.NC$PDP.POP.NC.pop.KDE[1:2
1], col = alpha("grey50", alpha =  0.15), lwd = 0.5)
}
lines(POP.resp.ST1.NC$x~POP.resp.ST1.NC$Group.1, col = "#FDE725FF")
polygon(x = c(POP.resp.ST1.NC$Group.1, rev(POP.resp.ST1.NC$Group.1)), y = c(POP.lower.int.ST1.NC, rev(POP.upper.i
nt.ST1.NC)), col = alpha("#FDE725FF", alpha = 0.5), border = NA)
```

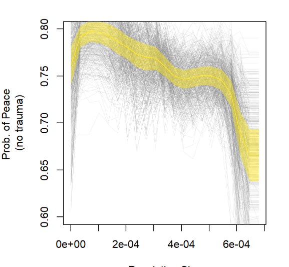

Supplemental Figure 40. Partial response of the probability of peace to variation in relative population size for non-coastal individuals while all other predictor variables' effects are held constant.

ENSO

```
ENS.list.NC <- list(pdp_partials.NC$PDP.ENS.NC.ENSO)
ENS.resp.ST1.NC <- aggregate(1-pdp_partials.NC$PDP.ENS.NC.yhat, by = ENS.list.NC, FUN = mean)
ENS.resp.ST1.sdE.NC <- aggregate(1-pdp_partials.NC$PDP.ENS.NC.yhat, by = ENS.list.NC, FUN = sd)
ENS.margin.ST1.NC <- qt(0.975, df = length(ENS.resp.ST1.NC$Group.1)-1) * ENS.resp.ST1.sdE.NC$x/sqrt(length(ENS.re
sp.ST1.NC$Group.1))#Calculate 95% CIs
ENS.lower.int.ST1.NC <- ENS.resp.ST1.NC$x - ENS.margin.ST1.NC#get the lower 95% CI boundary
ENS.upper.int.ST1.NC <- ENS.resp.ST1.NC$x + ENS.margin.ST1.NC#get the upper 95% CI boundary

par(pty = "s")
plot(ENS.resp.ST1.NC$x~ENS.resp.ST1.NC$Group.1, type = "l", ylab = "Prob. of Peace \n(no trauma)", xlab = "ENSO F
req", ylim = c(0.6, 0.8), col = "#6BCD5AFF")
for (i in 1:length(from.seq.plot)) {
  lines(1-pdp_partials.NC$PDP.ENS.NC.yhat[from.seq.plot[i]:to.seq.plot[i]]~pdp_partials.NC$PDP.ENS.NC.ENSO[1:21],
col = alpha("grey50", alpha = 0.15), lwd = 0.5)
}
lines(ENS.resp.ST1.NC$x~ENS.resp.ST1.NC$Group.1, col = "#6BCD5AFF")
polygon(x = c(ENS.resp.ST1.NC$Group.1, rev(ENS.resp.ST1.NC$Group.1)), y = c(ENS.lower.int.ST1.NC, rev(ENS.upper.i
nt.ST1.NC)), col = alpha("#6BCD5AFF", alpha = 0.5), border = NA)
```

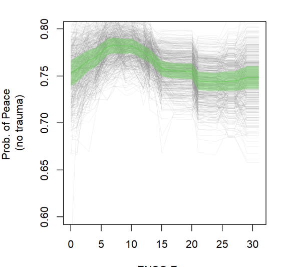

Supplemental Figure 41. Partial response of the probability of peace to variation in ENSO frequency per century for non-coastal individuals while all other predictor variables' effects are held constant.

State

```
State.list.NC <- list(pdp_partials.NC$PDP.STT.NC.Individuals_state)
State.resp.ST1.NC <- aggregate(1-pdp_partials.NC$PDP.STT.NC.yhat, by = State.list.NC, FUN = mean)
State.resp.ST1.sdE.NC <- aggregate(1-pdp_partials.NC$PDP.STT.NC.yhat, by = State.list.NC, FUN = sd)
State.margin.ST1.NC <- qt(0.975, df = length(State.resp.ST1.NC$Group.1)-1) * State.resp.ST1.sdE.NC$x/sqrt(length(
State.resp.ST1.NC$Group.1))#Calculate 95% CIs
State.lower.int.ST1.NC <- State.resp.ST1.NC$x - State.margin.ST1.NC#get the lower 95% CI boundary
State.upper.int.ST1.NC <- State.resp.ST1.NC$x + State.margin.ST1.NC#get the upper 95% CI boundary

par(pty = "s")
plot(y = State.resp.ST1.NC$x, x = as.numeric(State.resp.ST1.NC$Group.1), pch = 19, col = c("black","black","blac
k"), ylab = "Prob. of Peace \n(no trauma)", xlab = "State Presence", ylim = c(0.6, 0.8), xaxt = "n")
for (i in 1:length(from.seq.plot)) {
  lines(1-pdp_partials.NC$PDP.STT.NC.yhat[from.seq.plot[i]:to.seq.plot[i]]~pdp_partials.NC$PDP.STT.NC.Individuals
_state[1:21], col = alpha("grey50", alpha = 0.15), lwd = 0.5)
}
lines(State.resp.ST1.NC$x~State.resp.ST1.NC$Group.1, col = alpha("black", alpha = 0.5), lwd = 2)
points(y = State.resp.ST1.NC$x, x = as.numeric(State.resp.ST1.NC$Group.1), pch = 19, col = c("black","black","bla
ck"), cex = 1.5)
axis(side = 1, at = c(1,2,3), labels = c(0,1,2), tick = TRUE)
```

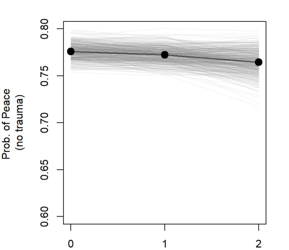

Supplemental Figure 42. Partial response of the probability of peace to variation in sociopolitical complexity for non-coastal individuals while all other predictor variables' effects are held constant.

Now make a full plot of all partials for non-coastal individuals.

```
from.seq.plot <- seq(1, 21000, 21)
to.seq.plot <- seq(21, 21000, 21)

par(mfrow = c(2,3))
par(pty = "s")
plot(MNP.resp.ST1.NC$x~MNP.resp.ST1.NC$Group.1, type = "l", ylab = "Prob. of Peace \n(no trauma)", xlab = "Mean P
recip.", ylim = c(0.60,0.825), col = "#482576FF")
for (i in 1:length(from.seq.plot)) {
  lines(1-pdp_partials.NC$PDP.MNP.NC.yhat[from.seq.plot[i]:to.seq.plot[i]]~pdp_partials.NC$PDP.MNP.NC.MNprcp20km[
1:21], col = alpha("grey50", alpha = 0.15), lwd = 0.5)
}

lines(MNP.resp.ST1.NC$x~MNP.resp.ST1.NC$Group.1, col = "#31668EFF")
polygon(x = c(MNP.resp.ST1.NC$Group.1, rev(MNP.resp.ST1.NC$Group.1)), y = c(MNP.lower.int.ST1.NC, rev(MNP.upper.i
nt.ST1.NC)), col = alpha("#31668EFF", alpha = 0.5), border = NA)

par(pty = "s")
plot(SDT.resp.ST1.NC$x~SDT.resp.ST1.NC$Group.1, type = "l", ylab = "", yaxt = "n", xlab = "SDT", ylim = c(0.60,0.
825), col = "#1E9C89FF")
for (i in 1:length(from.seq.plot)) {
  lines(1-pdp_partials.NC$PDP.SDT.NC.yhat[from.seq.plot[i]:to.seq.plot[i]]~pdp_partials.NC$PDP.SDT.NC.SDtemp20km[
1:21], col = alpha("grey50", alpha = 0.15), lwd = 0.5)
}

lines(SDT.resp.ST1.NC$x~SDT.resp.ST1.NC$Group.1, col = "#1E9C89FF")
polygon(x = c(SDT.resp.ST1.NC$Group.1, rev(SDT.resp.ST1.NC$Group.1)), y = c(SDT.lower.int.ST1.NC, rev(SDT.upper.i
nt.ST1.NC)), col = alpha("#1E9C89FF", alpha = 0.5), border = NA)


plot(MNT.resp.ST1.NC$x~MNT.resp.ST1.NC$Group.1, type = "l", ylab = "", yaxt = "n", xlab = "Mean Temp.", ylim = c(
0.60,0.825), col = "#31668EFF")
for (i in 1:length(from.seq.plot)) {
  lines(1-pdp_partials.NC$PDP.MNT.NC.yhat[from.seq.plot[i]:to.seq.plot[i]]~pdp_partials.NC$PDP.MNT.NC.MNtemp20km[
1:21], col = alpha("grey50", alpha = 0.15), lwd = 0.5)
}
lines(MNT.resp.ST1.NC$x~MNT.resp.ST1.NC$Group.1, col = "#482576FF")
polygon(x = c(MNT.resp.ST1.NC$Group.1, rev(MNT.resp.ST1.NC$Group.1)), y = c(MNT.lower.int.ST1.NC, rev(MNT.upper.i
nt.ST1.NC)), col = alpha("#482576FF", alpha = 0.5), border = NA)


par(pty = "s")
plot(POP.resp.ST1.NC$x~POP.resp.ST1.NC$Group.1, type = "l", ylab = "Prob. of Peace\n(no trauma)", xlab = "Populat
ion (KDE)", ylim = c(0.60,0.825), col = "#FDE725FF")
for (i in 1:length(from.seq.plot)) {
  lines(1-pdp_partials.NC$PDP.POP.NC.yhat[from.seq.plot[i]:to.seq.plot[i]]~pdp_partials.NC$PDP.POP.NC.pop.KDE[1:2
1], col = alpha("grey50", alpha = 0.15), lwd = 0.5)
}

lines(POP.resp.ST1.NC$x~POP.resp.ST1.NC$Group.1, col = "#FDE725FF")
polygon(x = c(POP.resp.ST1.NC$Group.1, rev(POP.resp.ST1.NC$Group.1)), y = c(POP.lower.int.ST1.NC, rev(POP.upper.i
nt.ST1.NC)), col = alpha("#FDE725FF", alpha = 0.5), border = NA)

par(pty = "s")
plot(ENS.resp.ST1.NC$x~ENS.resp.ST1.NC$Group.1, type = "l", ylab = "", yaxt = "n", xlab = "ENSO Freq.", ylim = c(
0.60,0.825), col = "#6BCD5AFF")
for (i in 1:length(from.seq.plot)) {
  lines(1-pdp_partials.NC$PDP.ENS.NC.yhat[from.seq.plot[i]:to.seq.plot[i]]~pdp_partials.NC$PDP.ENS.NC.ENSO[1:21],
col = alpha("grey50", alpha = 0.15), lwd = 0.5)
}

lines(ENS.resp.ST1.NC$x~ENS.resp.ST1.NC$Group.1, col = "#6BCD5AFF")
polygon(x = c(ENS.resp.ST1.NC$Group.1, rev(ENS.resp.ST1.NC$Group.1)), y = c(ENS.lower.int.ST1.NC, rev(ENS.upper.i
nt.ST1.NC)), col = alpha("#6BCD5AFF", alpha = 0.5), border = NA)

par(pty = "s")
plot(y = State.resp.ST1.NC$x, x = as.numeric(State.resp.ST1.NC$Group.1), pch = 19, col = "black", ylab = "", yaxt
= "n", xlab = "State Presence", ylim = c(0.60,0.825), xaxt = "n")
for (i in 1:length(from.seq.plot)) {
  lines(1-pdp_partials.NC$PDP.STT.NC.yhat[from.seq.plot[i]:to.seq.plot[i]]~pdp_partials.NC$PDP.STT.NC.Individuals
_state[1:21], col = alpha("grey50", alpha = 0.15), lwd = 0.5)
}
lines(State.resp.ST1.NC$x~State.resp.ST1.NC$Group.1, col = alpha("black", alpha = 0.5), lwd = 2)
points(y = State.resp.ST1.NC$x, x = as.numeric(State.resp.ST1.NC$Group.1), pch = 19, col = "black", cex = 1.5)
axis(side = 1, at = c(1,2,3), labels = c(0,1,2), tick = TRUE)
```

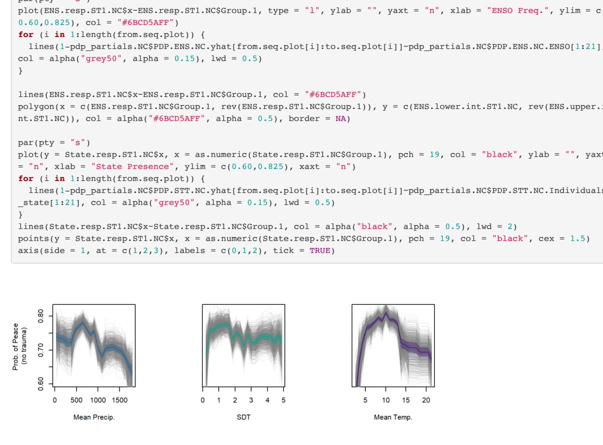

Supplemental Figure 43. Partial response plots for the probability of trauma to all predictor variables for non-coastal individuals. Like with the coastal results, these partial responses provide intriguing, useful, insights though do not quite test all of the predictions due to the expectation of climatic interactions that beget instability or uncertainty which is more likely to impact peace and violence than single climate dimensions.

As with coastal individuals, to investigate these interactions, we use 3d partial response plots from a sub-sample of 100 models. We use 100 models because it takes >25 minutes to generate these 3d partial response data for a single model and the computational time for all 1000 would be >420 hours. Combined with the coastal models, this would represent >840 hours of computational time. As we run 3d partial plots for sociopolitical complexity as well, the computation time would be >1680 hours (~70 days).

To visualize the 3d partial responses, we need to aggregate the data from the 100 models.

Mean Temp.

```
MNT.3d.p <- aggregate(pdp3d_partials.NC$MNT.KDE.PDP.NC.yhat~pdp3d_partials.NC$MNT.KDE.PDP.NC.MNtemp20km+pdp3d_par
tials.NC$MNT.KDE.PDP.NC.pop.KDE, FUN = mean)
colnames(MNT.3d.p) <- c("MNtemp20km", "pop.KDE", "yhat")

library(ggplot2)
NC.MNT.3d <- ggplot() +
  theme(aspect.ratio = 1, panel.background = element_blank()) + #make the plot square
  geom_raster(data = MNT.3d.p, aes(x = MNtemp20km, y = pop.KDE, fill = 1-yhat)) +
  scale_fill_viridis_b(n.breaks = 11, breaks = seq(0.575,0.825,length.out = 11), limits = c(0.575,0.825), option
= "turbo", direction = -1, labels = c("0.575", "", "0.625", " ", "0.675", " ", "0.725", " ", "0.775", " ", "0.82
5")) +
  xlab("Mean Temp. (C)") +
  ylab("Pop. Density (KDE)") +
  labs(fill = "Prob. of Peace \n(no trauma)") +
  scale_x_continuous(breaks = round(seq(min(MNT.3d.p$MNtemp20km), max(MNT.3d.p$MNtemp20km), len = 7))) +
  scale_y_continuous(breaks = round(seq(min(MNT.3d.p$pop.KDE), max(MNT.3d.p$pop.KDE), len = 8),4)) +
  geom_vline(xintercept = mean(pdp3d_partials.NC$MNT.KDE.PDP.NC.MNtemp20km), col = "grey95", size = 1.5) +
  geom_hline(yintercept = mean(pdp3d_partials.NC$MNT.KDE.PDP.NC.pop.KDE), col = "grey95", size = 1.5)

#ggsave(filename = "NC.MNT.3d.png", plot = NC.MNT.3d, device = "png", height = 5, width = 5, units = "in", dpi =
300)
#ggsave(filename = "NC.MNT.3d.pdf", plot = NC.MNT.3d, device = "pdf", height = 5, width = 5, units = "in", dpi =
300)

NC.MNT.3d
```

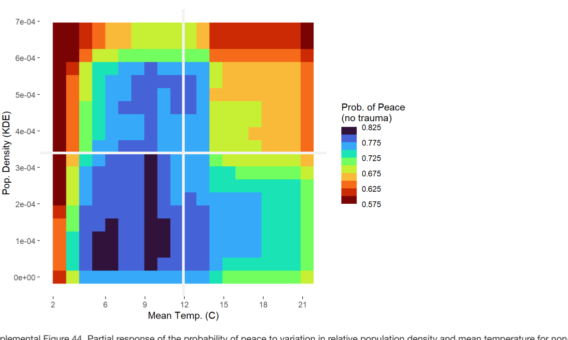

Supplemental Figure 44. Partial response of the probability of peace to variation in relative population density and mean temperature for non-coastal individuals while all other predictor variables' effects are held constant.

Mean Precip.

```
MNP.3d.p <- aggregate(pdp3d_partials.NC$MNP.KDE.PDP.NC.yhat~pdp3d_partials.NC$MNP.KDE.PDP.NC.MNprcp20km+pdp3d_par
tials.NC$MNP.KDE.PDP.NC.pop.KDE, FUN = mean)
colnames(MNP.3d.p) <- c("MNprcp20km", "pop.KDE", "yhat")

library(ggplot2)
NC.MNP.3d <- ggplot() +
  theme(aspect.ratio = 1, panel.background = element_blank()) + #make the plot square
  geom_raster(data = MNP.3d.p, aes(x = MNprcp20km, y = pop.KDE, fill = 1-yhat)) +
  scale_fill_viridis_b(n.breaks = 11, breaks = seq(0.575,0.825,length.out = 11), limits = c(0.575,0.825), option
= "turbo", direction = -1, labels = c("0.575", "", "0.625", " ", "0.675", " ", "0.725", " ", "0.775", " ", "0.82
5")) +
  xlab("Mean Precip. (mm/yr)") +
  ylab("Pop. Density (KDE)") +
  labs(fill = "Prob. of Peace \n(no trauma)") +
  scale_x_continuous(breaks = round(seq(min(MNP.3d.p$MNprcp20km), max(MNP.3d.p$MNprcp20km), len = 7))) +
  scale_y_continuous(breaks = round(seq(min(MNP.3d.p$pop.KDE), max(MNP.3d.p$pop.KDE), len = 8),4)) +
  geom_vline(xintercept = mean(pdp3d_partials.NC$MNP.KDE.PDP.NC.MNprcp20km), col = "grey95", size = 1.5) +
  geom_hline(yintercept = mean(pdp3d_partials.NC$MNP.KDE.PDP.NC.pop.KDE), col = "grey95", size = 1.5)

#ggsave(filename = "NC.MNP.3d.png", plot = NC.MNP.3d, device = "png", height = 5, width = 5, units = "in", dpi =
300)
#ggsave(filename = "NC.MNP.3d.pdf", plot = NC.MNP.3d, device = "pdf", height = 5, width = 5, units = "in", dpi =
300)

NC.MNP.3d
```

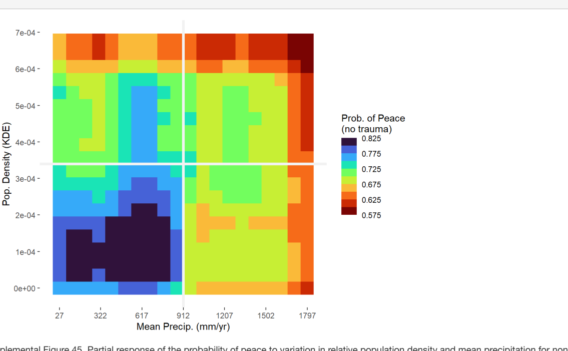

Supplemental Figure 45. Partial response of the probability of peace to variation in relative population density and mean precipitation for non-coastal individuals while all other predictor variables' effects are held constant.

St. Dev. Temp.

```
SDT.3d.p <- aggregate(pdp3d_partials.NC$SDT.KDE.PDP.NC.yhat~pdp3d_partials.NC$SDT.KDE.PDP.NC.SDtemp20km+pdp3d_par
tials.NC$SDT.KDE.PDP.NC.pop.KDE, FUN = mean)
colnames(SDT.3d.p) <- c("SDT", "pop.KDE", "yhat")

library(ggplot2)
NC.SDT.3d <- ggplot() +
  theme(aspect.ratio = 1, panel.background = element_blank()) + #make the plot square
  geom_raster(data = SDT.3d.p, aes(x = SDT, y = pop.KDE, fill = 1-yhat)) +
  scale_fill_viridis_b(n.breaks = 11, breaks = seq(0.575,0.825,length.out = 11), limits = c(0.575,0.825), option
= "turbo", direction = -1, labels = c("0.575", "", "0.625", " ", "0.675", " ", "0.725", " ", "0.775", " ", "0.82
5")) +
  xlab("SDT") +
  ylab("Pop. Density (KDE)") +
  labs(fill = "Prob. of Peace \n(no trauma)") +
  scale_x_continuous(breaks = round(seq(min(SDT.3d.p$SDT), max(SDT.3d.p$SDT), len = 7),2)) +
  scale_y_continuous(breaks = round(seq(min(SDT.3d.p$pop.KDE), max(SDT.3d.p$pop.KDE), len = 8),4)) +
  geom_vline(xintercept = mean(pdp3d_partials.NC$SDT.KDE.PDP.NC.SDtemp20km), col = "grey95", size = 1.5) +
  geom_hline(yintercept = mean(pdp3d_partials.NC$SDT.KDE.PDP.NC.pop.KDE), col = "grey95", size = 1.5)

#ggsave(filename = "NC.SDT.3d.png", plot = NC.SDT.3d, device = "png", height = 5, width = 5, units = "in", dpi =
300)
#ggsave(filename = "NC.SDT.3d.pdf", plot = NC.SDT.3d, device = "pdf", height = 5, width = 5, units = "in", dpi =
300)

NC.SDT.3d
```

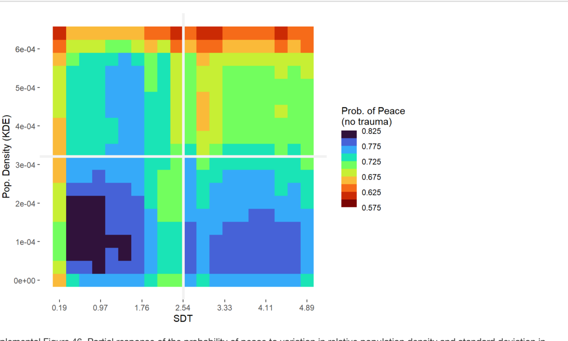

Supplemental Figure 46. Partial response of the probability of peace to variation in relative population density and standard deviation in temperature for non-coastal individuals while all other predictor variables' effects are held constant.

ENSO

```
ENS.3d.p <- aggregate(pdp3d_partials.NC$ENS.KDE.PDP.NC.yhat~pdp3d_partials.NC$ENS.KDE.PDP.NC.ENSO+pdp3d_partials.
NC$ENS.KDE.PDP.NC.pop.KDE, FUN = mean)
colnames(ENS.3d.p) <- c("ENSO", "pop.KDE", "yhat")

#The gaps in ENSO values are not even (for example, we go from 0 to 1 and then 1 to 3). This causes an issue with
plotting because we don't have continuous variables. We can address this by generating an estimate for the missin
g variables - which we do below.

#use tidyverse for add_row
library(tidyverse)

   st <- seq(1,400,20) #start row sequence
   en <- seq(20,400,20) #end row sequence
   pop.kde.seq <- unique(ENS.3d.p$pop.KDE)#sequence of pop values from our partial predictions

   ENS.POP.3d <- data.frame()#empty dataframe to hold ouptut

for (i in 1:20) { #for our 20 ENSO / pop.KDE combinations
     test.df <- ENS.3d.p[st[i]:en[i],] #subset to just 1 combination
     test.df <- test.df %>% #add the missing ENSO observation values as rows
       add_row(ENSO = c(2,5,7,10,12,15,18,20,23,25,28,30),
             pop.KDE = pop.kde.seq[i], #insert the correct pop.KDE value from the sequence
             yhat = NA, #make yhat (prob no trauma) NA - we will fill in later
             .before = 3) #place the new rows before row 3

     test.df <- arrange(test.df, by = test.df$ENSO) #order the df by ENSO in ascending order
     test.df$yhat <- approx(test.df$yhat, n = 32)$y #use the approx function to estimate the missing yhat values
(just uses the prior and succeeding observation to each missing one - think of it like an average of the two)
     ENS.POP.3d <- rbind(ENS.POP.3d, test.df)#put the new rows into a dataframe
     }

#This makes the plot before our filling in the dataframe just to see what it looks like and to compare
#ggplot() +
#  theme(aspect.ratio = 1, panel.background = element_blank()) + #make the plot square
#  geom_raster(data = ENS.3d.p, aes(x = ENSO, y = pop.KDE, fill = yhat)) +
#  scale_fill_viridis(n.breaks = 10) +
#  xlab("ENSO Freq. / Century") +
#  ylab("Pop. Density (KDE)") +
#  labs(fill = "Prob. No Trauma") +
#  scale_x_continuous(breaks = round(seq(min(ENS.3d.p$ENSO), max(ENS.3d.p$ENSO), len = 7))) +
#  scale_y_continuous(breaks = round(seq(min(ENS.3d.p$pop.KDE), max(ENS.3d.p$pop.KDE), len = 7),4))

#this makes the plot with the filled in data we did above so there aren't odd white gaps in the plot
NC.ENS.3d <- ggplot() +
  theme(aspect.ratio = 1, panel.background = element_blank()) + #make the plot square
  geom_raster(data = ENS.POP.3d, aes(x = ENSO, y = pop.KDE, fill = 1-yhat)) +
  scale_fill_viridis_b(n.breaks = 11, breaks = seq(0.575,0.825,length.out = 11), limits = c(0.575,0.825), option
= "turbo", direction = -1, labels = c("0.575", "", "0.625", " ", "0.675", " ", "0.725", " ", "0.775", " ", "0.82
5")) +
  xlab("ENSO Freq. / Century") +
  ylab("Pop. Density (KDE)") +
  labs(fill = "Prob. of Peace \n(no trauma)") +
  scale_x_continuous(breaks = round(seq(min(ENS.POP.3d$ENSO), max(ENS.POP.3d$ENSO), len = 7))) +
  scale_y_continuous(breaks = round(seq(min(ENS.POP.3d$pop.KDE), max(ENS.POP.3d$pop.KDE), len = 8),4)) +
  geom_vline(xintercept = 15.5, col = "grey95", size = 1.5) +
  geom_hline(yintercept = mean(pdp3d_partials.NC$ENS.KDE.PDP.NC.pop.KDE), col = "grey95", size = 1.5)

#ggsave(filename = "NC.ENS.3d.png", plot = NC.ENS.3d, device = "png", height = 5, width = 5, units = "in", dpi =
300)
#ggsave(filename = "NC.ENS.3d.pdf", plot = NC.ENS.3d, device = "pdf", height = 5, width = 5, units = "in", dpi =
300)

NC.ENS.3d
```

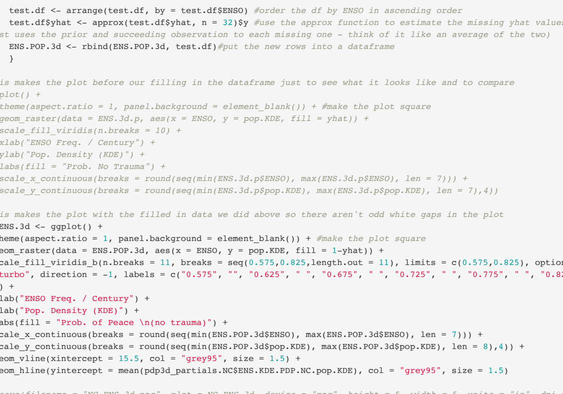

Supplemental Figure 47. Partial response of the probability of peace to variation in relative population density and ENSO frequency per century for non-coastal individuals while all other predictor variables' effects are held constant.

Mean Temp and Mean Precip.

```
MNT.MNP.3d.p <- aggregate(pdp3d_partials.NC$MNP.MNT.PDP.NC.yhat~pdp3d_partials.NC$MNP.MNT.PDP.NC.MNtemp20km+pdp3d
_partials.NC$MNP.MNT.PDP.NC.MNprcp20km, FUN = mean)
colnames(MNT.MNP.3d.p) <- c("MNtemp20km", "MNprcp20km", "yhat")

NC.MNT.MNP.3d <- ggplot() +
  theme(aspect.ratio = 1, panel.background = element_blank()) + #make the plot square
  geom_raster(data = MNT.MNP.3d.p, aes(x = MNtemp20km, y = MNprcp20km, fill = 1-yhat)) +
  scale_fill_viridis_b(n.breaks = 11, breaks = seq(0.575,0.825,length.out = 11), limits = c(0.575,0.825), option
= "turbo", direction = -1, labels = c("0.575", "", "0.625", " ", "0.675", " ", "0.725", " ", "0.775", " ", "0.82
5")) +
  xlab("Mean Temp. (C)") +
  ylab("Mean Precip. (mm/year)") +
  labs(fill = "Prob. of Peace \n(no trauma)") +
  scale_x_continuous(breaks = round(seq(min(MNT.MNP.3d.p$MNtemp20km), max(MNT.MNP.3d.p$MNtemp20km), len = 7))) +
  scale_y_continuous(breaks = round(seq(min(MNT.MNP.3d.p$MNprcp20km), max(MNT.MNP.3d.p$MNprcp20km), len = 7),4))
+
  geom_vline(xintercept = mean(pdp3d_partials.NC$MNT.KDE.PDP.NC.MNtemp20km), col = "grey95", size = 1.5) +
  geom_hline(yintercept = mean(pdp3d_partials.NC$MNP.KDE.PDP.NC.MNprcp20km), col = "grey95", size = 1.5)

#ggsave(filename = "NC.MNT.MNP.3d.png", plot = NC.MNT.MNP.3d, device = "png", height = 5, width = 5, units = "i
n", dpi = 300)
#ggsave(filename = "NC.MNT.MNP.3d.pdf", plot = NC.MNT.MNP.3d, device = "pdf", height = 5, width = 5, units = "i
n", dpi = 300)

NC.MNT.MNP.3d
```

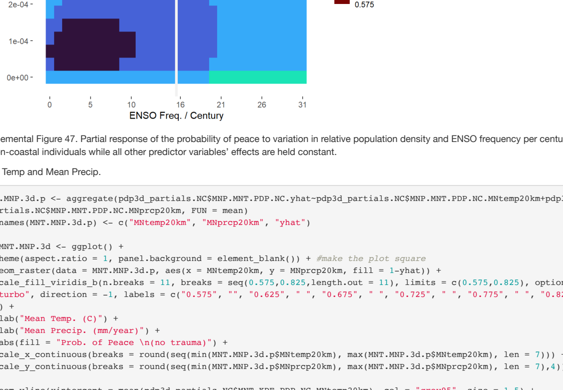

Supplemental Figure 48. Partial response of the probability of peace to variation in mean precipitation and mean temperature for non-coastal individuals while all other predictor variables' effects are held constant.

Sociopolitical Complexity Interaction

To explore interactions with sociopolitical complexity we generate an additional set of interaction plots for both coastal and non-coastal individuals.

Beginning with coastal data: Mean Temp.

```
MNT.3d.p <- aggregate(pdp3d_spc.C$MNT.SPC.PDP.C.yhat~pdp3d_spc.C$MNT.SPC.PDP.C.MNtemp20km+pdp3d_spc.C$MNT.SPC.PD
P.C.Individuals_state, FUN = mean)
colnames(MNT.3d.p) <- c("MNtemp20km", "SPC", "yhat")

C.MNT.3d <- ggplot() +
  theme(aspect.ratio = 1, panel.background = element_blank()) + #make the plot square
  geom_raster(data = MNT.3d.p, aes(x = MNtemp20km, y = SPC, fill = 1-yhat)) +
  scale_fill_viridis_b(n.breaks = 10, breaks = seq(0.625,0.85,0.025), limits = c(0.625,0.85), option = "turbo", d
irection = -1, labels = c("0.625", "", "", "0.700", "", " ", "0.775", " ", "", "0.850")) +
  xlab("Mean Temp. (C)") +
  ylab("Socio-Pol. Complexity") +
  labs(fill = "Prob. of Peace \n(no trauma)") +
  scale_x_continuous(breaks = round(seq(min(MNT.3d.p$MNtemp20km), max(MNT.3d.p$MNtemp20km), len = 7))) +
 # scale_y_continuous(breaks = round(seq(min(MNT.3d.p$SPC), max(MNT.3d.p$SPC), len = 7),4)) +
  geom_vline(xintercept = mean(pdp3d_spc.C$MNT.SPC.PDP.C.MNtemp20km), col = "grey95", size = 1.5) +
  geom_hline(yintercept = 1.5, col = "grey95", size = 1.5)+
  geom_hline(yintercept = 2.5, col = "grey95", size = 1.5)

C.MNT.3d
```

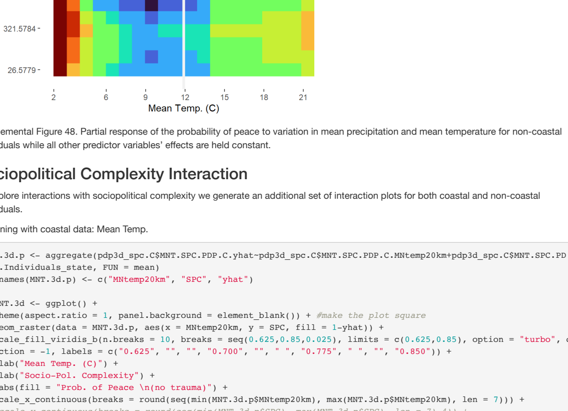

Supplemental Figure 49. Partial response of the probability of peace to variation in socipolitical complexity and mean temperature for coastal individuals while all other predictor variables' effects are held constant.

Mean Precip.

```
MNP.3d.p <- aggregate(pdp3d_spc.C$MNP.SPC.PDP.C.yhat~pdp3d_spc.C$MNP.SPC.PDP.C.MNprcp20km+pdp3d_spc.C$MNP.SPC.PD
P.C.Individuals_state, FUN = mean)
colnames(MNP.3d.p) <- c("MNprcp20km", "SPC", "yhat")

C.MNP.3d <- ggplot() +
  theme(aspect.ratio = 1, panel.background = element_blank()) + #make the plot square
  geom_raster(data = MNP.3d.p, aes(x = MNprcp20km, y = SPC, fill = 1-yhat)) +
  scale_fill_viridis_b(n.breaks = 10, breaks = seq(0.625,0.85,0.025), limits = c(0.625,0.85), option = "turbo", d
irection = -1, labels = c("0.625", "", "", "0.700", "", " ", "0.775", " ", "", "0.850")) +
  xlab("Mean Precip. (mm/yr)") +
  ylab("Socio-Pol. Complexity") +
  labs(fill = "Prob. of Peace \n(no trauma)") +
  scale_x_continuous(breaks = round(seq(min(MNP.3d.p$MNprcp20km), max(MNP.3d.p$MNprcp20km), len = 7))) +
 # scale_y_continuous(breaks = round(seq(min(MNP.3d.p$SPC), max(MNP.3d.p$SPC), len = 7),4)) +
  geom_vline(xintercept = mean(pdp3d_spc.C$MNP.SPC.PDP.C.MNprcp20km), col = "grey95", size = 1.5) +
  geom_hline(yintercept = 1.5, col = "grey95", size = 1.5)+
  geom_hline(yintercept = 2.5, col = "grey95", size = 1.5)

C.MNP.3d
```

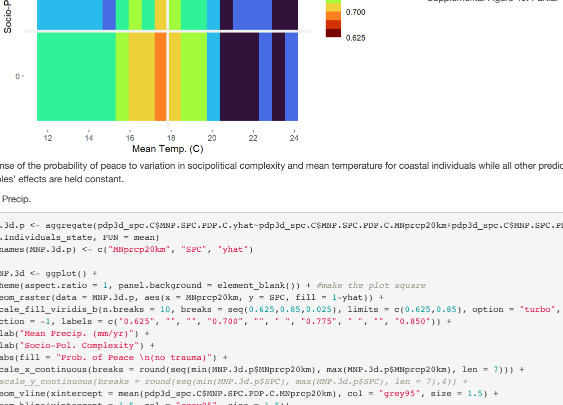

Supplemental Figure 50. Partial response of the probability of peace to variation in socipolitical complexity and mean precipitation for coastal individuals while all other predictor variables' effects are held constant.

SST

```
SST.3d.p <- aggregate(pdp3d_spc.C$SST.SPC.PDP.C.yhat~pdp3d_spc.C$SST.SPC.PDP.C.SST+pdp3d_spc.C$SST.SPC.PDP.C.Indi
viduals_state, FUN = mean)
colnames(SST.3d.p) <- c("SST", "SPC", "yhat")

C.SST.3d <- ggplot() +
  theme(aspect.ratio = 1, panel.background = element_blank()) + #make the plot square
  geom_raster(data = SST.3d.p, aes(x = SST, y = SPC, fill = 1-yhat)) +
  scale_fill_viridis_b(n.breaks = 10, breaks = seq(0.625,0.85,0.025), limits = c(0.625,0.85), option = "turbo", d
irection = -1, labels = c("0.625", "", "", "0.700", "", " ", "0.775", " ", "", "0.850")) +
  xlab("SST (C)") +
  ylab("Socio-Pol. Complexity") +
  labs(fill = "Prob. of Peace \n(no trauma)") +
  scale_x_continuous(breaks = round(seq(min(SST.3d.p$SST), max(SST.3d.p$SST), len = 7))) +
 # scale_y_continuous(breaks = round(seq(min(SST.3d.p$SPC), max(SST.3d.p$SPC), len = 7),4)) +
  geom_vline(xintercept = mean(pdp3d_spc.C$SST.SPC.PDP.C.SST), col = "grey95", size = 1.5) +
  geom_hline(yintercept = 1.5, col = "grey95", size = 1.5)+
  geom_hline(yintercept = 2.5, col = "grey95", size = 1.5)

C.SST.3d
```

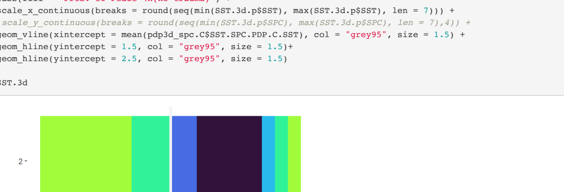

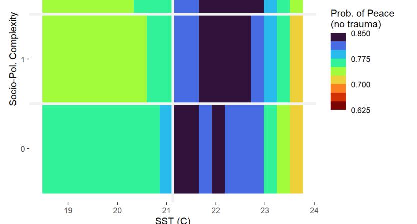

Supplemental Figure 51. Partial response of the probability of peace to variation in socipolitical complexity and sea surface temperature for coastal individuals while all other predictor variables' effects are held constant.

ENSO

```
ENS.3d.p <- aggregate(pdp3d_spc.C$ENS.SPC.PDP.C.yhat~pdp3d_spc.C$ENS.SPC.PDP.C.ENSO+pdp3d_spc.C$ENS.SPC.PDP.C.Ind
ividuals_state, FUN = mean)
colnames(ENS.3d.p) <- c("ENSO", "SPC", "yhat")
ENS.3d.p$yhat[which(ENS.3d.p$yhat > 1.0)] <- 1.0

#use tidyverse for add_row
library(tidyverse)

   st <- seq(1,400,20) #start row sequence
   en <- seq(20,400,20) #end row sequence
   SPC.seq <- unique(ENS.3d.p$SPC)#sequence of pop values from our partial predictions

   ENS.SPC.3d <- data.frame()#empty dataframe to hold ouptut

for (i in 1:3) { #for our 20 ENSO / pop.KDE combinations
     test.df <- ENS.3d.p[st[i]:en[i],] #subset to just 1 combination
     test.df <- test.df %>% #add the missing ENSO observation values as rows
       add_row(ENSO = c(2,5,7,10,12,15,18,20,23,25,28,30),
             SPC = SPC.seq[i], #insert the correct pop.KDE value from the sequence
             yhat = NA, #make yhat (prob no trauma) NA - we will fill in later
             .before = 3) #place the new rows before row 3

     test.df <- arrange(test.df, by = test.df$ENSO) #order the df by ENSO in ascending order
     test.df$yhat <- approx(test.df$yhat, n = 32)$y #use the approx function to estimate the missing yhat values
(just uses the prior and succeeding observation to each missing one - think of it like an average of the two)
     ENS.SPC.3d <- rbind(ENS.SPC.3d, test.df)#put the new rows into a dataframe
}

C.ENS.3d <- ggplot() +
  theme(aspect.ratio = 1, panel.background = element_blank()) + #make the plot square
  geom_raster(data = ENS.SPC.3d, aes(x = ENSO, y = SPC, fill = 1-yhat)) +
  scale_fill_viridis_b(n.breaks = 10, breaks = seq(0.625,0.85,0.025), limits = c(0.625,0.85), option = "turbo", d
irection = -1, labels = c("0.625", "", "", "0.700", "", " ", "0.775", " ", "", "0.850")) +
  xlab("ENSO Freq. / Century") +
  ylab("Socio-Pol. Complexity") +
  labs(fill = "Prob. of Peace \n(no trauma)") +
  scale_x_continuous(breaks = round(seq(min(ENS.SPC.3d$ENSO), max(ENS.SPC.3d$ENSO), len = 7))) +
 # scale_y_continuous(breaks = round(seq(min(ENS.POP.3d$pop.KDE), max(ENS.POP.3d$pop.KDE), len = 7),4)) +
  geom_vline(xintercept = mean(pdp3d_spc.C$ENS.SPC.PDP.C.ENSO), col = "grey95", size = 1.5) +
  geom_hline(yintercept = 1.5, col = "grey95", size = 1.5)+
  geom_hline(yintercept = 2.5, col = "grey95", size = 1.5)

C.ENS.3d
```

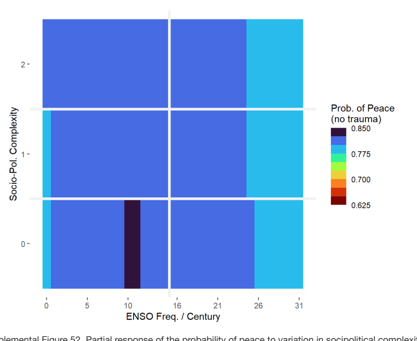

Supplemental Figure 52. Partial response of the probability of peace to variation in socipolitical complexity and ENSO frequency per century for coastal individuals while all other predictor variables' effects are held constant.

Population

```
POP.3d.p <- aggregate(pdp3d_spc.C$KDE.SPC.PDP.C.yhat~pdp3d_spc.C$KDE.SPC.PDP.C.pop.KDE+pdp3d_spc.C$KDE.SPC.PDP.C.
Individuals_state, FUN = mean)
colnames(POP.3d.p) <- c("Pop.KDE", "SPC", "yhat")

C.KDE.3d <- ggplot() +
  theme(aspect.ratio = 1, panel.background = element_blank()) + #make the plot square
  geom_raster(data = POP.3d.p, aes(x = Pop.KDE, y = SPC, fill = 1-yhat)) +
  scale_fill_viridis_b(n.breaks = 10, breaks = seq(0.625,0.85,0.025), limits = c(0.625,0.85), option = "turbo", d
irection = -1, labels = c("0.625", "", "", "0.700", "", " ", "0.775", " ", "", "0.850")) +
  xlab("Pop. Density (KDE)") +
  ylab("Socio-Pol. Complexity") +
  labs(fill = "Prob. of Peace \n(no trauma)") +
  scale_x_continuous(breaks = round(seq(min(POP.3d.p$Pop.KDE), max(POP.3d.p$Pop.KDE), len = 7))) +
 # scale_y_continuous(breaks = round(seq(min(SST.3d.p$SPC), max(SST.3d.p$SPC), len = 7),4)) +
  geom_vline(xintercept = mean(pdp3d_spc.C$KDE.SPC.PDP.C.pop.KDE), col = "grey95", size = 1.5) +
  geom_hline(yintercept = 1.5, col = "grey95", size = 1.5)+
  geom_hline(yintercept = 2.5, col = "grey95", size = 1.5)

C.KDE.3d
```

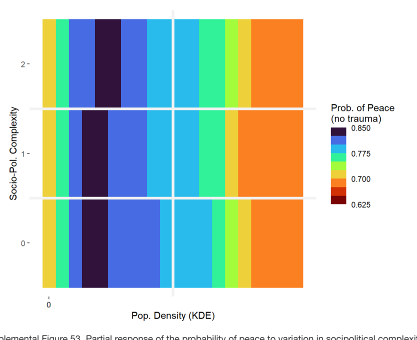

Supplemental Figure 53. Partial response of the probability of peace to variation in socipolitical complexity and relative popluation size for coastal individuals while all other predictor variables' effects are held constant.

Overall what we see in the 3d partial plots exploring the interaction of sociopolitical complexity with other variables is that complexity continues to have very little impact and almost no interaction with the other variables for predicting trauma. The key to reading this is y-axis (vertical) variation along shows the scope of complexity effect while horizontal variation along the x axis visualizes climate (or demography) scope of effect. What we see is very little vertical variation and a lot more horizontal variability. In every case what we see is color variation almost exclusively along the x axes (climate and population variables). The y axes (sociopolitical complexity) have consistent coloration (no variation) meaning little complexity effect.

Next we can check sociopolitical complexity interactions for inland individuals:

Mean Temp.

```
MNT.3d.p <- aggregate(pdp3d_spc.NC$MNT.SPC.PDP.NC.yhat~pdp3d_spc.NC$MNT.SPC.PDP.NC.MNtemp20km+pdp3d_spc.NC$MNT.SP
C.PDP.NC.Individuals_state, FUN = mean)
colnames(MNT.3d.p) <- c("MNtemp20km", "SPC", "yhat")

NC.MNT.3d <- ggplot() +
  theme(aspect.ratio = 1, panel.background = element_blank()) + #make the plot square
  geom_raster(data = MNT.3d.p, aes(x = MNtemp20km, y = SPC, fill = 1-yhat)) +
  scale_fill_viridis_b(n.breaks = 11, breaks = seq(0.575,0.825,length.out = 11), limits = c(0.575,0.825), option
= "turbo", direction = -1, labels = c("0.575", "", "0.625", " ", "0.675", " ", "0.725", " ", "0.775", " ", "0.82
5")) +
  xlab("Mean Temp. (C)") +
  ylab("Socio-Pol. Complexity") +
  labs(fill = "Prob. of Peace \n(no trauma)") +
  scale_x_continuous(breaks = round(seq(min(MNT.3d.p$MNtemp20km), max(MNT.3d.p$MNtemp20km), len = 7))) +
 # scale_y_continuous(breaks = round(seq(min(MNT.3d.p$SPC), max(MNT.3d.p$SPC), len = 7),4)) +
  geom_vline(xintercept = mean(pdp3d_spc.NC$MNT.SPC.PDP.NC.MNtemp20km), col = "grey95", size = 1.5) +
  geom_hline(yintercept = 1.5, col = "grey95", size = 1.5)+
  geom_hline(yintercept = 2.5, col = "grey95", size = 1.5)

NC.MNT.3d
```

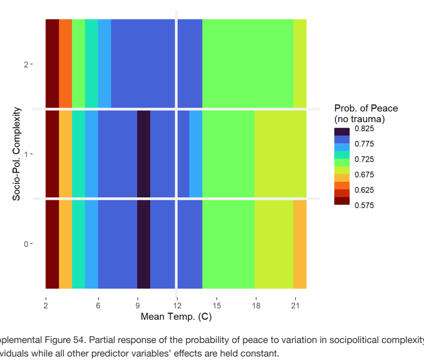

Supplemental Figure 54. Partial response of the probability of peace to variation in socipolitical complexity and mean temperature for non-coastal individuals while all other predictor variables' effects are held constant.

Mean Precip.

```
MNP.3d.p <- aggregate(pdp3d_spc.NC$MNP.SPC.PDP.NC.yhat~pdp3d_spc.NC$MNP.SPC.PDP.NC.MNprcp20km+pdp3d_spc.NC$MNP.SP
C.PDP.NC.Individuals_state, FUN = mean)
colnames(MNP.3d.p) <- c("MNprcp20km", "SPC", "yhat")

NC.MNP.3d <- ggplot() +
  theme(aspect.ratio = 1, panel.background = element_blank()) + #make the plot square
  geom_raster(data = MNP.3d.p, aes(x = MNprcp20km, y = SPC, fill = 1-yhat)) +
  scale_fill_viridis_b(n.breaks = 11, breaks = seq(0.575,0.825,length.out = 11), limits = c(0.575,0.825), option
= "turbo", direction = -1, labels = c("0.575", "", "0.625", " ", "0.675", " ", "0.725", " ", "0.775", " ", "0.82
5")) +
  xlab("Mean Precip. (mm/yr)") +
  ylab("Socio-Pol. Complexity") +
  labs(fill = "Prob. of Peace \n(no trauma)") +
  scale_x_continuous(breaks = round(seq(min(MNP.3d.p$MNprcp20km), max(MNP.3d.p$MNprcp20km), len = 7))) +
 # scale_y_continuous(breaks = round(seq(min(MNP.3d.p$SPC), max(MNP.3d.p$SPC), len = 7),4)) +
  geom_vline(xintercept = mean(pdp3d_spc.NC$MNP.SPC.PDP.NC.MNprcp20km), col = "grey95", size = 1.5) +
  geom_hline(yintercept = 1.5, col = "grey95", size = 1.5)+
  geom_hline(yintercept = 2.5, col = "grey95", size = 1.5)

NC.MNP.3d
```

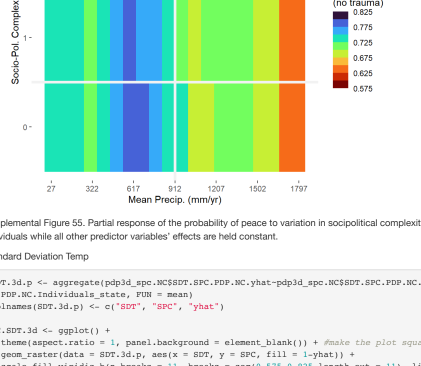

Supplemental Figure 55. Partial response of the probability of peace to variation in socipolitical complexity and mean precipitation for non-coastal individuals while all other predictor variables' effects are held constant.

Standard Deviation Temp

```
SDT.3d.p <- aggregate(pdp3d_spc.NC$SDT.SPC.PDP.NC.yhat~pdp3d_spc.NC$SDT.SPC.PDP.NC.SDtemp20km+pdp3d_spc.NC$SDT.SP
C.PDP.NC.Individuals_state, FUN = mean)
colnames(SDT.3d.p) <- c("SDT", "SPC", "yhat")

NC.SDT.3d <- ggplot() +
  theme(aspect.ratio = 1, panel.background = element_blank()) + #make the plot square
  geom_raster(data = SDT.3d.p, aes(x = SDT, y = SPC, fill = 1-yhat)) +
  scale_fill_viridis_b(n.breaks = 11, breaks = seq(0.575,0.825,length.out = 11), limits = c(0.575,0.825), option
= "turbo", direction = -1, labels = c("0.575", "", "0.625", " ", "0.675", " ", "0.725", " ", "0.775", " ", "0.82
5")) +
  xlab("Std. Dev. Temp. (C)") +
  ylab("Socio-Pol. Complexity") +
  labs(fill = "Prob. of Peace \n(no trauma)") +
  scale_x_continuous(breaks = round(seq(min(SDT.3d.p$SDT), max(SDT.3d.p$SDT), len = 7))) +
 # scale_y_continuous(breaks = round(seq(min(SDT.3d.p$SPC), max(SDT.3d.p$SPC), len = 7),4)) +
  geom_vline(xintercept = mean(pdp3d_spc.NC$SDT.SPC.PDP.NC.SDtemp20km), col = "grey95", size = 1.5) +
  geom_hline(yintercept = 1.5, col = "grey95", size = 1.5)+
  geom_hline(yintercept = 2.5, col = "grey95", size = 1.5)

NC.SDT.3d
```

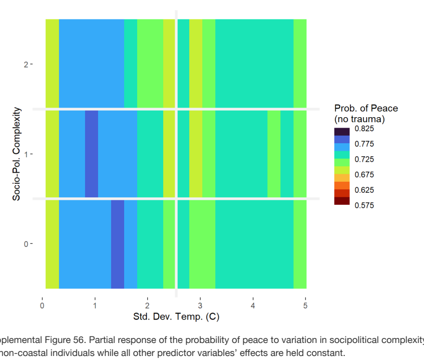

Supplemental Figure 56. Partial response of the probability of peace to variation in socipolitical complexity and standard deviation in temperature for non-coastal individuals while all other predictor variables' effects are held constant.

ENSO

```
ENS.3d.p <- aggregate(pdp3d_spc.NC$ENS.SPC.PDP.NC.yhat~pdp3d_spc.NC$ENS.SPC.PDP.NC.ENSO+pdp3d_spc.NC$ENS.SPC.PDP.
NC.Individuals_state, FUN = mean)
colnames(ENS.3d.p) <- c("ENSO", "SPC", "yhat")
ENS.3d.p$yhat[which(ENS.3d.p$yhat > 1.0)] <- 1.0

#use tidyverse for add_row
library(tidyverse)

   st <- seq(1,400,20) #start row sequence
   en <- seq(20,400,20) #end row sequence
   SPC.seq <- unique(ENS.3d.p$SPC)#sequence of pop values from our partial predictions

   ENS.SPC.3d <- data.frame()#empty dataframe to hold ouptut

for (i in 1:3) { #for our 20 ENSO / pop.KDE combinations
     test.df <- ENS.3d.p[st[i]:en[i],] #subset to just 1 combination
     test.df <- test.df %>% #add the missing ENSO observation values as rows
       add_row(ENSO = c(2,5,7,10,12,15,18,20,23,25,28,30),
             SPC = SPC.seq[i], #insert the correct pop.KDE value from the sequence
             yhat = NA, #make yhat (prob no trauma) NA - we will fill in later
             .before = 3) #place the new rows before row 3

     test.df <- arrange(test.df, by = test.df$ENSO) #order the df by ENSO in ascending order
     test.df$yhat <- approx(test.df$yhat, n = 32)$y #use the approx function to estimate the missing yhat values
(just uses the prior and succeeding observation to each missing one - think of it like an average of the two)
     ENS.SPC.3d <- rbind(ENS.SPC.3d, test.df)#put the new rows into a dataframe
     }

#this makes the plot with the filled in data we did above so there aren't odd white gaps in the plot
NC.ENS.3d <- ggplot() +
  theme(aspect.ratio = 1, panel.background = element_blank()) + #make the plot square
  geom_raster(data = ENS.SPC.3d, aes(x = ENSO, y = SPC, fill = 1-yhat)) +
  scale_fill_viridis_b(n.breaks = 11, breaks = seq(0.575,0.825,length.out = 11), limits = c(0.575,0.825), option
= "turbo", direction = -1, labels = c("0.575", "", "0.625", " ", "0.675", " ", "0.725", " ", "0.775", " ", "0.82
5")) +
  xlab("ENSO Freq. / Century") +
  ylab("Socio-Pol. Complexity") +
  labs(fill = "Prob. of Peace \n(no trauma)") +
  scale_x_continuous(breaks = round(seq(min(ENS.SPC.3d$ENSO), max(ENS.SPC.3d$ENSO), len = 7))) +
 # scale_y_continuous(breaks = round(seq(min(ENS.POP.3d$pop.KDE), max(ENS.POP.3d$pop.KDE), len = 7),4)) +
  geom_vline(xintercept = mean(pdp3d_spc.NC$ENS.SPC.PDP.NC.ENSO), col = "grey95", size = 1.5) +
  geom_hline(yintercept = 1.5, col = "grey95", size = 1.5)+
  geom_hline(yintercept = 2.5, col = "grey95", size = 1.5)

NC.ENS.3d
```

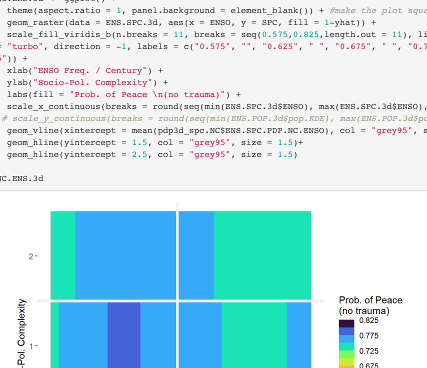

Supplemental Figure 57. Partial response of the probability of peace to variation in socipolitical complexity and ENSO frequency per century for non-coastal individuals while all other predictor variables' effects are held constant.

Population

```
POP.3d.p <- aggregate(pdp3d_spc.NC$KDE.SPC.PDP.NC.yhat~pdp3d_spc.NC$KDE.SPC.PDP.NC.pop.KDE+pdp3d_spc.NC$KDE.SPC.P
DP.NC.Individuals_state, FUN = mean)
colnames(POP.3d.p) <- c("Pop.KDE", "SPC", "yhat")

NC.KDE.3d <- ggplot() +
  theme(aspect.ratio = 1, panel.background = element_blank()) + #make the plot square
  geom_raster(data = POP.3d.p, aes(x = Pop.KDE, y = SPC, fill = 1-yhat)) +
  scale_fill_viridis_b(n.breaks = 11, breaks = seq(0.575,0.825,length.out = 11), limits = c(0.575,0.825), option
= "turbo", direction = -1, labels = c("0.575", "", "0.625", " ", "0.675", " ", "0.725", " ", "0.775", " ", "0.82
5")) +
  xlab("Pop. Density (KDE)") +
  ylab("Socio-Pol. Complexity") +
  labs(fill = "Prob. of Peace \n(no trauma)") +
  scale_x_continuous(breaks = round(seq(min(POP.3d.p$Pop.KDE), max(POP.3d.p$Pop.KDE), len = 7))) +
 # scale_y_continuous(breaks = round(seq(min(SST.3d.p$SPC), max(SST.3d.p$SPC), len = 7),4)) +
  geom_vline(xintercept = mean(pdp3d_spc.NC$KDE.SPC.PDP.NC.pop.KDE), col = "grey95", size = 1.5) +
  geom_hline(yintercept = 1.5, col = "grey95", size = 1.5)+
  geom_hline(yintercept = 2.5, col = "grey95", size = 1.5)

NC.KDE.3d
```

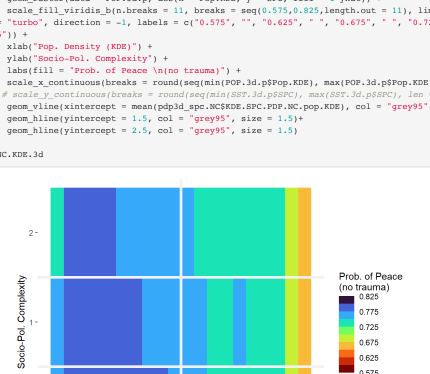

Supplemental Figure 58. Partial response of the probability of peace to variation in socipolitical complexity and relative population size for non-coastal individuals while all other predictor variables' effects are held constant.

Taphonomic Correction

Here we replicate the main portion of the study above employing taphonomically corrected KDEs using the Bluhm and Surovell (2019) updated, continuous, global taphonomic correction. For computational time sake, we run a 10% sample, generating 100 rather than 1000 RF models. The 10% sample of 100 RF models using taphonomically corrected KDEs produces analytically the same results as the 1000 models using the uncorrected KDEs.

First step in redoing the analysis is we need to resample the data to obtain the corrected KDEs.

```
cluster <- parallel::makeCluster(20, type = "PSOCK")
doParallel::registerDoParallel(cl = cluster)#setup the cluster of cores for use in parallel processing
foreach::getDoParRegistered()#TRUE means cluster is registered
foreach::getDoParWorkers()#Returned value should match # cores (20 in this case)

#make sure we have the individual data
indv_data <- read.csv("./data/indv_data.csv")

start_time <- Sys.time()#track when procedure begins

#Create a loop to extract individual values and iterate 100 times
indv.population <- foreach(i = 1:length(indv_data$RecordID), .combine = rbind, .packages = "data.table") %dopar%
{
  #each session needs to load the indv data
    indv_data <- read.csv("./data/indv_data.csv")
    #select the focal individual
    indv <- indv_data[i,]
    #make a blank dataframe to hold the individual's population reconstruction
    pop.result <- data.frame()

    #Function to load an Rdata file and assign it a new name so that we can load either CKDE as the necessary one
for the individual
    loadRData <- function(fileName){
    #loads an RData file, and returns it
    load(fileName)
    get(ls()[ls() != "fileName"])
    }
  #select the appropriate ckde data (coast or non-coast)
    if (indv$Region == "Far S coast" |indv$Region == "C coast" |indv$Region == "N coast" |indv$Region == "S coas
t" |indv$Region == "Ecuador coast"){
      Andes.ckde <- Coast.ckde.corr } else {
      Andes.ckde <- NCoast.ckde.corr
      }

    ckde.df <- as.data.frame(Andes.ckde$res.matrix) #pull out the data by year

    #loop to get 100 samples per individual
    for(j in 1:100) { #repeat the steps in the loop 100 times for each individual)
    kde.sample <- ckde.df[sample(ncol(ckde.df[1:1000]), 1)] #randomly grab 1 KDE fit from the 1000 we generated
    kde.vals <- data.frame("pop" = kde.sample, "year" = seq(20000, 0, -1)) #make a dataframe of that single KDE
    colnames(kde.vals) <- c("pop", "year")#make sure the columns are named correctly
    rownames(kde.vals) <- c()#drop row names
    population <- subset(kde.vals, year >=indv$end.date.bp & year <=indv$start.date.bp, select = pop) #Subset the
KDE for the individual's time window
    pop.val <- population[sample(nrow(population), 1),] #get one value which corresponds to one value from one ye
ar and add it to an output dataframe
    pop.result <- rbind(pop.result, pop.val)
    }

  #reset the column name for eas of use
  colnames(pop.result) <- "pop.KDE"

  return(pop.result)
  }

stopCluster(cluster)

#create an object for all the population data
indv.data.corr.pop.resampled <- indv.population

#save the population data
save(indv.data.corr.pop.resampled, file = "./data/indv.data.corr.pop.resampled.Rdata")

#record when procedure ended
end_time <- Sys.time()
#check how long it took to run
end_time - start_time
```

Then link the climate and trauma data per individual (100 samples) with their taphonomically corrected KDE values (100 samples).

```
#load the climate data for the first of the 100 randomly sampled sets of data
indv.data.resampled.corr.pop <- read.fst(path = "./data/indv.data.full.fst", from = 1, to = 799200)

#load the taphonomically corrected population sample (100 sets)
load("./data/indv.data.corr.pop.resampled.Rdata")

#for the population data, assign a unique run number to each of the 100 iterations per indiviudal
indv.data.corr.pop.resampled$Run <- rep(seq(1:100), 7992)

#order this so each individual's 1st sample is next to each other, their 2nd sample next to each other, etc. This
means the first 7992 rows of the dataset are all unique individuals as is the second set of 7992 and so on until
we have all 100 replicates. This also puts the taphonomically corrected KDE data in the exact same order as the r
est of our data per individual so we can link the datasets.
indv.data.corr.pop.resampled <- indv.data.corr.pop.resampled[order(indv.data.corr.pop.resampled$Run),]

#add the taphonomically corrected KDE to the dataset for all individuals (100 iterations)
indv.data.resampled.corr.pop$corr.pop.KDE <- indv.data.corr.pop.resampled$pop.KDE
```

Next, we get the min and max values for predictor variables as we will need those for getting partial response plots.

```
#lets get min and max data for our variables
variable <- read.fst("./data/indv.data.full.fst", column = c("MNtemp20km", "Region_Fact")) #MNT
minMNT.C <- min(variable$MNtemp20km[which(variable$Region_Fact == "coastal")])
medMNT.C <- median(variable$MNtemp20km[which(variable$Region_Fact == "coastal")])
maxMNT.C <- max(variable$MNtemp20km[which(variable$Region_Fact == "coastal")])

minMNT.NC <- min(variable$MNtemp20km[which(variable$Region_Fact == "non-coastal")])
medMNT.NC <- median(variable$MNtemp20km[which(variable$Region_Fact == "non-coastal")])
maxMNT.NC <- max(variable$MNtemp20km[which(variable$Region_Fact == "non-coastal")])

variable <- read.fst("./data/indv.data.full.fst", column = c("SDtemp20km", "Region_Fact")) #SDT
minSDT.NC <- min(variable$SDtemp20km[which(variable$Region_Fact == "non-coastal")])
medSDT.NC <- median(variable$SDtemp20km[which(variable$Region_Fact == "non-coastal")])
maxSDT.NC <- max(variable$SDtemp20km[which(variable$Region_Fact == "non-coastal")])

variable <- read.fst("./data/indv.data.full.fst", column = c("MNprcp20km", "Region_Fact")) #MNP
minMNP.C <- min(variable$MNprcp20km[which(variable$Region_Fact == "coastal")])
medMNP.C <- median(variable$MNprcp20km[which(variable$Region_Fact == "coastal")])
maxMNP.C <- max(variable$MNprcp20km[which(variable$Region_Fact == "coastal")])

minMNP.NC <- min(variable$MNprcp20km[which(variable$Region_Fact == "non-coastal")])
medMNP.NC <- median(variable$MNprcp20km[which(variable$Region_Fact == "non-coastal")])
maxMNP.NC <- max(variable$MNprcp20km[which(variable$Region_Fact == "non-coastal")])

#use the taphonomically corrected data for population
minPOP.C <- min(indv.data.resampled.corr.pop$corr.pop.KDE[which(indv.data.resampled.corr.pop$Region_Fact == "coas
tal")])
medPOP.C <- median(indv.data.resampled.corr.pop$corr.pop.KDE[which(indv.data.resampled.corr.pop$Region_Fact == "c
oastal")])
maxPOP.C <- max(indv.data.resampled.corr.pop$corr.pop.KDE[which(indv.data.resampled.corr.pop$Region_Fact == "coas
tal")])

minPOP.NC <- min(indv.data.resampled.corr.pop$corr.pop.KDE[which(indv.data.resampled.corr.pop$Region_Fact == "non
-coastal")])
medPOP.NC <- median(indv.data.resampled.corr.pop$corr.pop.KDE[which(indv.data.resampled.corr.pop$Region_Fact == "
non-coastal")])
maxPOP.NC <- max(indv.data.resampled.corr.pop$corr.pop.KDE[which(indv.data.resampled.corr.pop$Region_Fact == "non
-coastal")])

variable <- read.fst("./data/indv.data.full.fst", column = c("SST", "Region_Fact")) #CVP
minSST.C <- min(variable$SST[which(variable$Region_Fact == "coastal")])
medSST.C <- median(variable$SST[which(variable$Region_Fact == "coastal")])
maxSST.C <- max(variable$SST[which(variable$Region_Fact == "coastal")])

variable <- read.fst("./data/indv.data.full.fst", column = c("ENSO", "Region_Fact")) #CVP
minENS.C <- min(variable$ENSO[which(variable$Region_Fact == "coastal")])
medENS.C <- median(variable$ENSO[which(variable$Region_Fact == "coastal")])
maxENS.C <- max(variable$ENSO[which(variable$Region_Fact == "coastal")])

minENS.NC <- min(variable$ENSO[which(variable$Region_Fact == "non-coastal")])
medENS.NC <- median(variable$ENSO[which(variable$Region_Fact == "non-coastal")])
maxENS.NC <- max(variable$ENSO[which(variable$Region_Fact == "non-coastal")])

rm(variable) #don't hang on to 79 million rows of data - just keep the min/max values we need for predicted parti
al grids
```

For the taphonomically replicated study, to save on computational time and intensity, we generate just the 2 dimensional partial dependence plots. If the 2d plots using taphonomically corrected KDEs directly emulate the 2d plots using uncorrected KDEs the output of the models is functionally the same and the 3d plots would also look similar (the 3d plots with uncorrected KDEs visualize the same data as the 2d plots but with one indpendent variable on the x axis, one on the y axis, and the predicted response in color).

```
#Make the grids for 2d partial response (so we can get the influence of each variables while all other variables
are held at their average effect)
PDP.MNT.C <- data.frame(MNtemp20km = seq(minMNT.C, maxMNT.C, length.out = 21))
PDP.MNT.NC <- data.frame(MNtemp20km = seq(minMNT.NC, maxMNT.NC, length.out = 21))

PDP.SDT.NC <- data.frame(SDtemp20km = seq(minSDT.NC, maxSDT.NC, length.out = 21))

PDP.MNP.C <- data.frame(MNprcp20km = seq(minMNP.C, maxMNP.C, length.out = 21))
PDP.MNP.NC <- data.frame(MNprcp20km = seq(minMNP.NC, maxMNP.NC, length.out = 21))

PDP.POP.C <- data.frame(corr.pop.KDE = seq(minPOP.C, maxPOP.C, length.out = 21))
PDP.POP.NC <- data.frame(corr.pop.KDE = seq(minPOP.NC, maxPOP.NC, length.out = 21))

PDP.SST.C <- data.frame(SST = seq(minSST.C, maxSST.C, length.out = 21))

PDP.ENS.C <- data.frame(ENSO = seq(minENS.C, maxENS.C, length.out = 21))
PDP.ENS.NC <- data.frame(ENSO = seq(minENS.NC, maxENS.NC, length.out = 21))

PDP.STT.C <- data.frame(Individuals_state = c(rep(0, times = 7), rep(1, times = 7), rep(2, times = 7)))
PDP.STT.NC <- data.frame(Individuals_state = c(rep(0, times = 7), rep(1, times = 7), rep(2, times = 7)))

#Make the grids for 2d partial response (so we can get the influence of each variables while all other variables
are held at their average effect)
PDP.MNT.C <- data.frame(MNtemp20km = seq(minMNT.C, maxMNT.C, length.out = 21))
PDP.MNT.NC <- data.frame(MNtemp20km = seq(minMNT.NC, maxMNT.NC, length.out = 21))

PDP.SDT.NC <- data.frame(SDtemp20km = seq(minSDT.NC, maxSDT.NC, length.out = 21))

PDP.MNP.C <- data.frame(MNprcp20km = seq(minMNP.C, maxMNP.C, length.out = 21))
PDP.MNP.NC <- data.frame(MNprcp20km = seq(minMNP.NC, maxMNP.NC, length.out = 21))

PDP.POP.C <- data.frame(pop.KDE = seq(minPOP.C, maxPOP.C, length.out = 21))
PDP.POP.NC <- data.frame(pop.KDE = seq(minPOP.NC, maxPOP.NC, length.out = 21))

PDP.SST.C <- data.frame(SST = seq(minSST.C, maxSST.C, length.out = 21))

PDP.ENS.C <- data.frame(ENSO = seq(minENS.C, maxENS.C, length.out = 21))
PDP.ENS.NC <- data.frame(ENSO = seq(minENS.NC, maxENS.NC, length.out = 21))

PDP.STT.C <- data.frame(Individuals_state = c(rep(0, times = 7), rep(1, times = 7), rep(2, times = 7)))
PDP.STT.NC <- data.frame(Individuals_state = c(rep(0, times = 7), rep(1, times = 7), rep(2, times = 7)))
```

As part of model evaluation, we will calculate the Precision Recall AUC score. To do so we need to use the RF models we create to predict yes or no trauma for each individual in the dataset. Predict with ranger is a non-standard predict function, so we will write our own to use for this.

```
pred <- function(model, newdata)  {
  results <- as.data.frame(predict(model, newdata)$predictions)
  return(results)
}
```

Next, run the RF models.

The model analysis process is a memory (RAM) intensive process and is slow. The code below will work on a sufficiently powerful home computing device but may take a while to run. If running on a home device be sure to adjust the number of cores (ncores) accordingly.

```
#We are going to run 100 RF models to capture the range of variability in climate and population reconstructions.
Setting this sequence number lets us read in just the rows we need for each RF model to save on RAM.
from.seq <- seq(from = 1, to = 799200, by = 7992)
to.seq <- seq(from = 7992, to = 799200, by = 7992)

start <- Sys.time()

ncores <- 16
my.cluster <- makeCluster(ncores)
doParallel::registerDoParallel(cl = my.cluster)
foreach(i = 1:100, .packages = c("ranger","fst", "pROC", "data.table", "pdp", "dplyr", "yardstick", "caTools")) %
dopar%
  {
    #Get data in batches of 7992 (total individuals) so that each batch is 1 run
    indv.data <- indv.data.resampled.corr.pop[from.seq[i]:to.seq[i],
                                              c("Site.and.location", "total_trauma", "MNtemp20km","MNprcp20km", "
corr.pop.KDE",
                                                "Individuals_state","Region_Fact", "SST", "ENSO")]
#To run this without subsampling the sites - simply remove the code generating n.obs thorugh the indv.data %>% sa
mple_n(frq[1])

    #get the number of individuals per site
    n.obs <- indv.data %>%
      group_by(Site.and.location) %>%
      count(Site.and.location)
    #arrange the dataset by site.and.location to match with the output from count()
    indv.data <- indv.data %>%
      group_by(Site.and.location) %>%
      arrange(Site.and.location)
    #set a new column to be frq where that value is the number of individuals at the site
    indv.data$frq <- rep(n.obs$n, n.obs$n)
    indv.data$frq[which(indv.data$frq > 30)] <- 30
    #sample the dataframe taking no more than 30 individuals from a site (less if there are less than 30)
    indv.data <- indv.data %>%
      sample_n(frq[1])
    #
    #Select the individuals just from one region
    indv.data <- indv.data[which(indv.data$Region_Fact == "coastal"),]

    #Remove the Region_Fact variable
    indv.data <- subset(indv.data, select = -c(Region_Fact, Site.and.location, frq))
    #indv.data <- subset(indv.data, select = -c(Region_Fact, Site.and.location))

    indv.data <- indv.data[which(!is.na(indv.data$total_trauma)),]#drop NAs for trauma
    #indv.data$total_trauma <- factor(indv.data$total_trauma, levels = c("0", "1"))#set trauma factor level
    indv.data$Individuals_state <- factor(indv.data$Individuals_state, levels = c(0,1,2))#set state var as a fact
or level

    #Split the sample, preserving relative ratios of presence / absence of trauma
    split <- sample.split(indv.data$total_trauma, SplitRatio = 0.8) #train on 80% of data following standard cust
oms

    #add split TRUE / FALSE to the data
    indv.data$split <- split

    #Split the data into training and testing subsets
    data.train <- subset(indv.data, split == "TRUE")
    data.test <- subset(indv.data, split == "FALSE")

    #Remove the Split column
    data.train <- data.train[,-8]
    data.test <- data.test[,-8]

    #drop Split column from indv data
    indv.data <- indv.data[,-8]

    #regression with ranger
    RF.rang <- ranger(total_trauma ~ ., data = data.train, num.trees = 3000, mtry = 2, replace = TRUE, importance
= "permutation", min.node.size = 10)#General heuristics for choosing mtry value are square root of total # featur
es, 1/3 of the total # features, or log base 2 of the total # features. These all give a value between 1.98 and
2.5 - so we will do mtry of 2.

    #use the train data model to predict test data for model performance
    RF.rang.pred <- pred(RF.rang, newdata = data.test)

    #generate the reciever operating characteristic
    roctest <- roc(data.test$total_trauma, RF.rang.pred$`predict(model, newdata)$predictions`)
    #extract the area under the curve value
    aucRF <- pROC::auc(roctest)

    #calculate model logloss
    mod.logloss <-  ModelMetrics::logLoss(actual = data.test$total_trauma, pred = RF.rang.pred$`predict(model, ne
wdata)$predictions`)
    #conver model logloss to probability
   mod.logloss.prob <- exp(-ModelMetrics::logLoss(actual = data.test$total_trauma, pred = RF.rang.pred$`predict(m
odel, newdata)$predictions`))

    #run the model with all data
    RF.rang.all <- ranger(total_trauma ~ ., data = indv.data, num.trees = 3000, mtry = 2, replace = TRUE, importa
nce = "permutation", min.node.size = 10)

    #predict output for model evaluation on logloss
    RF.rang.pred.all <- pred(RF.rang, newdata = indv.data)

    #obtain model roc
    roctest.all <- roc(indv.data$total_trauma, RF.rang.pred.all$`predict(model, newdata)$predictions`)
    #extract model auc
    aucRF.all <- pROC::auc(roctest.all)

    #calculate the logloss of the model with all data included to measure overall model accuracy
    mod.logloss.all <-  ModelMetrics::logLoss(actual = indv.data$total_trauma, pred = RF.rang.pred.all$`predict(m
odel, newdata)$predictions`)
    #get the probability of correct class assignment
    mod.logloss.prob.all <- exp(-ModelMetrics::logLoss(actual = indv.data$total_trauma, pred = RF.rang.pred.all$`
predict(model, newdata)$predictions`))

    #generate the 2-dimensional partial response plots
    PDP.MNT.partial.C <- pdp::partial(RF.rang.all, pred.var = c("MNtemp20km"), pred.grid = PDP.MNT.C, prob = TRU
E)
    PDP.MNP.partial.C <- pdp::partial(RF.rang.all, pred.var = c("MNprcp20km"), pred.grid = PDP.MNP.C, prob = TRU
E)
    PDP.POP.partial.C <- pdp::partial(RF.rang.all, pred.var = c("corr.pop.KDE"), pred.grid = PDP.POP.C, prob = TR
UE)
    PDP.SST.partial.C <- pdp::partial(RF.rang.all, pred.var = c("SST"), pred.grid = PDP.SST.C, prob = TRUE)
    PDP.ENS.partial.C <- pdp::partial(RF.rang.all, pred.var = c("ENSO"), pred.grid = PDP.ENS.C, prob = TRUE)
    PDP.STT.partial.C <- pdp::partial(RF.rang.all, pred.var = c("Individuals_state"), pred.grid = PDP.STT.C, prob
= TRUE)

    #Put all the model evaluation data into one dataframe
    RF.results <- data.table(
      MSE.prederr = RF.rang$prediction.error,
      AUC = aucRF,
      AUC.all = aucRF.all,
      mod.logloss = mod.logloss,
      mod.logloss.prob = mod.logloss.prob,
      MSE.prederr.all = RF.rang.all$prediction.error,
      mod.logloss.all = mod.logloss.all,
      mod.logloss.prob.all = mod.logloss.prob.all,
      MNT_Imp = RF.rang$variable.importance[1],
```

```
    MNP_Imp = RF.rang$variable.importance[2],
    POP_Imp = RF.rang$variable.importance[3],
    SST_Imp = RF.rang$variable.importance[4],
    ENS_Imp = RF.rang$variable.importance[5],
    STT_Imp = RF.rang$variable.importance[6])

  #make a df of single variable partial responses
  PDP.partials <- data.frame(
        PDP.MNT.C = PDP.MNT.partial.C,
        PDP.MNP.C = PDP.MNP.partial.C,
        PDP.POP.C = PDP.POP.partial.C,
        PDP.SST.C = PDP.SST.partial.C,
        PDP.ENS.C = PDP.ENS.partial.C,
        PDP.STT.C = PDP.STT.partial.C
                              )
  #Make file path for model performance data
  mod_perf.file <- paste("./output/Performance_C_corr/model_perf_corrpop_", i, ".Rdata", sep = "")
  #make file path for single var partial response data
  mod_pdp_part.file <- paste("./output/PDP_Partials_C_corr/model_part_corrpop_",i,".Rdata", sep = "")
  #save performance and single partials
  save(PDP.partials, file = mod_pdp_part.file)
  save(RF.results, file = mod_perf.file)

  #clean the environment to hopefully cut down on the RAM used which is causing failures
  rm(list = c("indv.data", "RF.rang", "RF.rang.all", "RF.rang.pred", "RF.rang.pred.all", "roctest", "aucRF", "r
octest.all", "aucRF.all", "RF.results", "PDP.MNT.partial.C", "PDP.MNP.partial.C", "PDP.POP.partial.C", "PDP.SST.p
artial.C", "PDP.ENS.partial.C", "PDP.STT.partial.C", "mod_perf.file", "mod_pdp_part.file", "mod.logloss", "mod.lo
gloss.prob", "mod.logloss.all", "mod.logloss.prob.all"))

  gc(verbose = FALSE)#garbage collect to free up cleaned memory space


 }
stopCluster(my.cluster)

#Repeat for non-coastal

ncores <- 16
my.cluster <- makeCluster(ncores)
doParallel::registerDoParallel(cl = my.cluster)
foreach(i = 1:100, .packages = c("ranger","fst", "pROC", "data.table", "pdp", "dplyr", "yardstick", "caTools")) %
dopar% #"pdp"
 {

  #Get data in batches of 7992 (total individuals) so that each batch is 1 run
  indv.data <- indv.data.resampled.corr.pop[from.seq[i]:to.seq[i],
                                          c("Site.and.location", "total_trauma", "MNtemp20km","MNprcp20km", "
corr.pop.KDE",
                                            "Individuals_state","Region_Fact", "SDtemp20km", "ENSO")]

#To run this without subsampling the sites - simply remove the code generating n.obs thorugh the indv.data %>% sa
mple_n(frq[1])

  # get the number of individuals per site
  n.obs <- indv.data %>%
    group_by(Site.and.location) %>%
    count(Site.and.location)
  #arrange the dataset by site.and.location to match with the output from count()
  indv.data <- indv.data %>%
    group_by(Site.and.location) %>%
    arrange(Site.and.location)
  #set a new column to be frq where that value is the number of individuals at the site
  indv.data$frq <- rep(n.obs$n, n.obs$n)
  indv.data$frq[which(indv.data$frq > 30)] <- 30
  #sample the dataframe taking no more than 30 individuals from a site (less if there are less than 30)
  indv.data <- indv.data %>%
    sample_n(frq[1])

  #Select the individuals just from one region
  indv.data <- indv.data[which(indv.data$Region_Fact == "non-coastal"),]

  #Remove the Region_Fact variable
  indv.data <- subset(indv.data, select = -c(Region_Fact, Site.and.location, frq))
  #indv.data <- subset(indv.data, select = -c(Region_Fact, Site.and.location))

  indv.data <- indv.data[which(!is.na(indv.data$total_trauma)),]#drop NAs for trauma
 # indv.data$total_trauma <- factor(indv.data$total_trauma, levels = c("0", "1"))#set trauma factor level
  indv.data$Individuals_state <- factor(indv.data$Individuals_state, levels = c(0,1,2))#set state var as a fact
or level

  #Split the sample, preserving relative ratios of presence / absence of trauma
  split <- sample.split(indv.data$total_trauma, SplitRatio = 0.8) #train on 80% of data following standard cust
oms

  #add split TRUE / FALSE to the data
  indv.data$split <- split

  #Split the data into training and testing subsets
  data.train <- subset(indv.data, split == "TRUE")
  data.test <- subset(indv.data, split == "FALSE")

  #Remove the Split column
  data.train <- data.train[,-8]
  data.test <- data.test[,-8]

  #drop Split column from indv data
  indv.data <- indv.data[,-8]


  RF.rang <- ranger(total_trauma ~ ., data = data.train, num.trees = 3000, mtry = 2, replace = TRUE, importance
= "permutation", min.node.size = 10)#General heuristics for choosing mtry value are square root of total # featur
es, 1/3 of the total # features, or log base 2 of the total # features. These all give a value between 1.98 and
2.5 - so we will do mtry of 2.

  #predict
  RF.rang.pred <- pred(RF.rang, newdata = data.test)

  #get roc
  roctest <- roc(data.test$total_trauma, RF.rang.pred$`predict(model, newdata)$predictions`)
  #extract auc
  aucRF <- pROC::auc(roctest)

  #calculate logloss
   mod.logloss <-  ModelMetrics::logLoss(actual = data.test$total_trauma, pred = RF.rang.pred$`predict(model, n
ewdata)$predictions`)
 #convert to probability correct prediction
   mod.logloss.prob <- exp(-ModelMetrics::logLoss(actual = data.test$total_trauma, pred = RF.rang.pred$`predic
t(model, newdata)$predictions`))

  #rerun with all data
  RF.rang.all <- ranger(total_trauma ~ ., data = indv.data, num.trees = 3000, mtry = 2, replace = TRUE, importa
nce = "permutation", min.node.size = 10)

  #predict
  RF.rang.pred.all <- pred(RF.rang.all, newdata = indv.data)

  #calculate roc
  roctest.all <- roc(indv.data$total_trauma, RF.rang.pred.all$`predict(model, newdata)$predictions`)
  #extract auc
  aucRF.all <- pROC::auc(roctest.all)

  #calculate logloss for model accuracy
  mod.logloss.all <-  ModelMetrics::logLoss(actual = indv.data$total_trauma, pred = RF.rang.pred.all$`predict(m
odel, newdata)$predictions`)
  #convert logloss to probability of accurate prediction
  mod.logloss.prob.all <- exp(-ModelMetrics::logLoss(actual = indv.data$total_trauma, pred = RF.rang.pred.all$`
predict(model, newdata)$predictions`))

  #generate 2-dimensional partial response data
  PDP.MNT.partial.NC <- pdp::partial(RF.rang.all, pred.var = c("MNtemp20km"), pred.grid = PDP.MNT.NC, prob = TR
UE)
  PDP.MNP.partial.NC <- pdp::partial(RF.rang.all, pred.var = c("MNprcp20km"), pred.grid = PDP.MNP.NC, prob = TR
UE)
  PDP.POP.partial.NC <- pdp::partial(RF.rang.all, pred.var = c("corr.pop.KDE"), pred.grid = PDP.POP.NC, prob =
TRUE)
  PDP.SDT.partial.NC <- pdp::partial(RF.rang.all, pred.var = c("SDtemp20km"), pred.grid = PDP.SDT.NC, prob = TR
UE)
  PDP.ENS.partial.NC <- pdp::partial(RF.rang.all, pred.var = c("ENSO"), pred.grid = PDP.ENS.NC, prob = TRUE)
  PDP.STT.partial.NC <- pdp::partial(RF.rang.all, pred.var = c("Individuals_state"), pred.grid = PDP.STT.NC, pr
ob = TRUE)

  #Put all the model evaluation data into one dataframe
  RF.results <- data.table(
    MSE.prederr = RF.rang$prediction.error,
    AUC = aucRF,
    AUC.all = aucRF.all,
    mod.logloss = mod.logloss,
    mod.logloss.prob = mod.logloss.prob,
    MSE.prederr.all = RF.rang.all$prediction.error,
    mod.logloss.all = mod.logloss.all,
    mod.logloss.prob.all = mod.logloss.prob.all,
    MNT_Imp = RF.rang$variable.importance[1],
    MNP_Imp = RF.rang$variable.importance[2],
    POP_Imp = RF.rang$variable.importance[3],
    SDT_Imp = RF.rang$variable.importance[4],
    ENS_Imp = RF.rang$variable.importance[5],
    STT_Imp = RF.rang$variable.importance[6])

  #make a df of single variable partial responses
  PDP.partials <- data.frame(
        PDP.MNT.NC = PDP.MNT.partial.NC,
        PDP.MNP.NC = PDP.MNP.partial.NC,
        PDP.POP.NC = PDP.POP.partial.NC,
        PDP.SDT.NC = PDP.SDT.partial.NC,
        PDP.ENS.NC = PDP.ENS.partial.NC,
        PDP.STT.NC = PDP.STT.partial.NC
                              )
  #Make file path for model performance data
  mod_perf.file <- paste("./output/Performance_NC_corr/model_perf_corrpop_", i, ".Rdata", sep = "")
  #make file path for single var partial response data
  mod_pdp_part.file <- paste("./output/PDP_Partials_NC_corr/model_part_corrpop_",i,".Rdata", sep = "")
  #save performance and single partials
  save(PDP.partials, file = mod_pdp_part.file)
  save(RF.results, file = mod_perf.file)

  #clean the environment to hopefully cut down on the RAM used which is causing failures
  rm(list = c("indv.data", "RF.rang", "RF.rang.all", "RF.rang.pred", "RF.rang.pred.all", "roctest", "aucRF", "r
octest.all", "aucRF.all", "RF.results", "PDP.MNT.partial.NC", "PDP.MNP.partial.NC", "PDP.POP.partial.NC", "PDP.SD
T.partial.NC", "PDP.ENS.partial.NC", "PDP.STT.partial.NC", "mod_perf.file", "mod_pdp_part.file", "mod.logloss", "
mod.logloss.prob", "mod.logloss.all", "mod.logloss.prob.all"))

  gc(verbose = FALSE)#garbage collect to free up cleaned memory space

#    end <- Sys.time()
#time.total <- end - start
#time.total
 }

stopCluster(my.cluster)

  end <- Sys.time()
time.total <- end - start
time.total
```

We saved the output from each of the 100 model runs as its own file for ease of computation. Next, combine the relevant files together so that everything is in one place.

```
#Read in the paths for each Rdata partial response file
fn <- list.files(path = "./output/PDP_Partials_C_corr",
                 pattern = "Rdata",
                 full.names = TRUE)#obtain a list of all of the files (individual tifs) in the folder and place t
hem in a single vector

pdp_partials.corr.pop.C <- data.table() #make an empty data frame to store the 1k set of partial responses

for (i in 1:length(fn)) {
   load(fn[i])
   RF.partials <- data.table(PDP.partials)
   to.bind <- list(pdp_partials.corr.pop.C, RF.partials)
   pdp_partials.corr.pop.C <- rbindlist(to.bind)
}

#repeat for non-coastal output
fn <- list.files(path = "./output/PDP_Partials_NC_corr",
                 pattern = "Rdata",
                 full.names = TRUE)

pdp_partials.corr.pop.NC <- data.table()

for (i in 1:length(fn)) {
   load(fn[i])
   RF.partials <- data.table(PDP.partials)
   to.bind <- list(pdp_partials.corr.pop.NC, RF.partials)
   pdp_partials.corr.pop.NC <- rbindlist(to.bind)
}

#Repeat for model performance
fn2 <- list.files(path = "./output/Performance_C_corr",
                 pattern = "Rdata",
                 full.names = TRUE)

all_performance.corr.pop.C <- data.table()

for (i in 1:length(fn2)) {
   load(fn2[i])
   RF.results <- data.table(RF.results)
   to.bind <- list(all_performance.corr.pop.C, RF.results)
   all_performance.corr.pop.C <- rbindlist(to.bind)
}

#now non-coastal model performance
fn2 <- list.files(path = "./output/Performance_NC_corr",
                 pattern = "Rdata",
                 full.names = TRUE)

all_performance.corr.pop.NC <- data.table() #make an empty data frame to store the 10k set of partial responses

for (i in 1:length(fn2)) {
   load(fn2[i])
   RF.results <- data.table(RF.results)
   to.bind <- list(all_performance.corr.pop.NC, RF.results)
   all_performance.corr.pop.NC <- rbindlist(to.bind)
}

#Save the aggregated outputs
#save(all_performance.corr.pop.C, file = "./output/all_performance.corr.prob.pop.C.Rdata")
#save(all_performance.corr.pop.NC, file = "./output/all_performance.corr.prob.pop.NC.Rdata")

#save(pdp_partials.corr.pop.C, file = "./output/pdp_partials.corr.prob.pop.C.Rdata")
#save(pdp_partials.corr.pop.NC, file = "./output/pdp_partials.corr.pop.NC.Rdata")
```

Check the model performance values.

```
par(mfrow = c(2,2))
#par(pty = "s")
hist(all_performance.corr.pop.C$MSE.prederr, main = "", xlab = "MSE Pred. Error", breaks = 15, col = "#2D708EFF",
border = "white")
mtext("Coastal", side = 3, line = 1)
hist(all_performance.corr.pop.NC$MSE.prederr, main = "", xlab = "MSE Pred. Error", breaks = 15, col = "#73D055F
F", border = "white")
mtext("Non-Coastal", side = 3, line = 1)
hist(all_performance.corr.pop.C$AUC, main = "", xlab = "AUC", breaks = 15, col = "#2D708EFF", border = "white")
hist(all_performance.corr.pop.NC$AUC, main = "", xlab = "AUC", breaks = 15, col = "#73D055FF", border = "white")
```

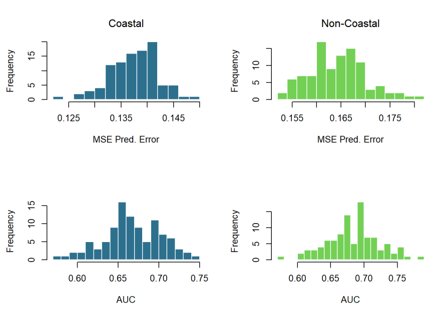

Supplemental Figure 59. Mean square error and area under the curve (AUC) model performance values for each of the 100 RF models for the coast (left, in blue) and non-coastal (right, in green) individuals with taphonomically corrected KDE values. AUC values suggest that the models for coast and non-coastal individuals perform acceptably well in predicting the absence or presence of trauma associated with violence and peace. Model outputs are normally distributed suggesting the central tendency of model performance accurately captures how strongly the climate and population data predict trauma.

Following standard practices, to capture total model accuracy performance we present the log-loss and model log-loss as probability of correct prediction from the models using all data.

```
par(mfrow = c(2,2))
#par(pty = "s")
hist(all_performance.corr.pop.C$mod.logloss.all, main = "", xlab = "Log-Loss", breaks = 15, col = "#2D708EFF", bo
rder = "white", xlim = c(0.24,0.29))
mtext("Coastal", side = 3, line = 1)
hist(all_performance.corr.pop.NC$mod.logloss.all, main = "", xlab = "Log-Loss", breaks = 15, col = "#73D055FF", b
order = "white", xlim = c(0.24,0.29))
mtext("Non-Coastal", side = 3, line = 1)
hist(all_performance.corr.pop.C$mod.logloss.prob.all, main = "", xlab = "Log-Loss as Probability", breaks = 15, c
ol = "#2D708EFF", border = "white", xlim = c(0.745,0.79))
hist(all_performance.corr.pop.NC$mod.logloss.prob.all, main = "", xlab = "Log-Loss as Probability", breaks = 15,
col = "#73D055FF", border = "white", xlim = c(0.745,0.79))
```

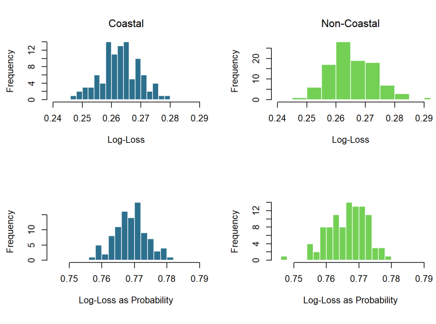

Supplemental Figure 60. In line with the AUC and MSE results, the log-loss results suggest the model performs acceptably well. We correctly predict trauma / no-trauma with around 77% accuracy; this is strong model performance given the data and the amount of uncertainty built into the models to capture temporal uncertainty, limit influence of large sites, and sample randomly for each split of the RF tree in each RF model. It is also the same explanatory power as using non-taphonomically corrected radiocarbon dates.

Next we look at variable importance.

```
MNT_Imp <- mean(all_performance.corr.pop.C$MNT_Imp)#1st
MNT_t <- t.test(all_performance.corr.pop.C$MNT_Imp)

MNP_Imp <- mean(all_performance.corr.pop.C$MNP_Imp)#2nd
MNP_t <- t.test(all_performance.corr.pop.C$MNP_Imp)

POP_Imp <- mean(all_performance.corr.pop.C$POP_Imp)#5th
POP_t <- t.test(all_performance.corr.pop.C$POP_Imp)

SST_Imp <- mean(all_performance.corr.pop.C$SST_Imp)#6th
SST_t <- t.test(all_performance.corr.pop.C$SST_Imp)

ENS_Imp <- mean(all_performance.corr.pop.C$ENS_Imp)#3rd
ENS_t <- t.test(all_performance.corr.pop.C$ENS_Imp)

STT_Imp <- mean(all_performance.corr.pop.C$STT_Imp)#4th
STT_t <- t.test(all_performance.corr.pop.C$STT_Imp)

#png(filename = "Coastal.VarImp.png", height = 5, width = 5, units = "in", res = 300)
par(pty = "s", oma = c(2,2,0,0))
plot(x = MNT_Imp, y = 6, xlim = c(0, 0.04), ylim = c(0,6), yaxt = "n", ylab = "", pch = 19, xlab = "", col = "#48
2576FF", cex = 1.5)
lines(x = c(MNT_t$conf.int[1], MNT_t$conf.int[2]), y = c(6,6), col = "white", lwd = 1)
points(x = MNP_Imp, y = 5, pch = 19, cex = 1.5, col = "#31668EFF")
lines(x = c(MNP_t$conf.int[1], MNP_t$conf.int[2]), y = c(5,5), col = "white")
points(x = ENS_Imp, y = 4, pch = 19, cex = 1.5, col = "#6BCD5AFF")
lines(x = c(ENS_t$conf.int[1], ENS_t$conf.int[2]), y = c(4,4), col = "white")
points(x = STT_Imp, y = 3, pch = 19, cex = 1.5, col = "black")
lines(x = c(STT_t$conf.int[1], STT_t$conf.int[2]), y = c(3,3), col = "white")
points(x = POP_Imp, y = 2, pch = 19, cex = 1.5, col = "#FDE725FF")
lines(x = c(POP_t$conf.int[1], POP_t$conf.int[2]), y = c(2,2), col = "white")
points(x = SST_Imp, y = 1, pch = 19, cex = 1.5, col = "#1E9C89FF")
lines(x = c(SST_t$conf.int[1], SST_t$conf.int[2]), y = c(1,1), col = "white")
axis(2, at = c(1,2,3,4,5,6), labels = FALSE, tick = TRUE)
mtext("Mean Temp.", at = 6, side = 2, line = 1, las = 1)
mtext("Mean Precip.", at = 5, side = 2, line = 1, las = 1)
mtext("ENSO Freq.", at = 4, side = 2, line = 1, las = 1)
mtext("State", at = 3, side = 2, line = 1, las = 1)
mtext("Population (KDE)", at = 2, side = 2, line = 1, las = 1)
mtext("Sea Surf. Temp.", at = 1, side = 2, line = 1, las = 1)
mtext("Variable Importance", side = 1, line = 2)
```

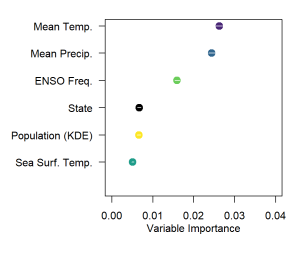


```
#dev.off()
```

Supplemental Figure 61. Variable importance for coastal models using taphonomically corrected KDEs. Colored dots provide the central tendency value for variable importance from the aggregate 100 models while the white line provides the confidence interval.

Next, we visualize the partial dependence results.

Mean Precip

```
#Get the aggregated predicted responses and make info for a confidence interval for precip
MNP.list.C <- list(pdp_partials.corr.pop.C$PDP.MNP.C.MNprcp20km)
MNP.resp.ST1.C <- aggregate(1-pdp_partials.corr.pop.C$PDP.MNP.C.yhat, by = MNP.list.C, FUN = mean)#Get the averag
e predicted probability no trauma for each precip value
MNP.resp.ST1.sdE.C <- aggregate(1-pdp_partials.corr.pop.C$PDP.MNP.C.yhat, by = MNP.list.C, FUN = sd)#Get the st d
ev in predicted probability no trauma for each precip value
MNP.margin.ST1.C <- qt(0.975, df = length(MNP.resp.ST1.C$Group.1)-1) * MNP.resp.ST1.sdE.C$x/sqrt(length(MNP.resp.
ST1.C$Group.1))#Calculate 95% CIs
MNP.lower.int.ST1.C <- MNP.resp.ST1.C$x - MNP.margin.ST1.C#get the lower 95% CI boundary
MNP.upper.int.ST1.C <- MNP.resp.ST1.C$x + MNP.margin.ST1.C#get the upper 95% CI boundary

from.seq.plot <- seq(1, 21000, 21)
to.seq.plot <- seq(21, 21000, 21)

par(pty = "s")
plot(MNP.resp.ST1.C$x~MNP.resp.ST1.C$Group.1, type = "l", ylab = "Prob. of Peace \n(no trauma)", xlab = "Mean Pre
cip.", ylim = c(0.65, 0.875), col = "#31668EFF")
for (i in 1:length(from.seq.plot)) {
  lines(1-pdp_partials.corr.pop.C$PDP.MNP.C.yhat[from.seq.plot[i]:to.seq.plot[i]]~pdp_partials.corr.pop.C$PDP.MN
P.C.MNprcp20km[1:21], col = alpha("grey50", alpha = 0.15), lwd = 0.5)
}
lines(MNP.resp.ST1.C$x~MNP.resp.ST1.C$Group.1, col = "#31668EFF")
polygon(x = c(MNP.resp.ST1.C$Group.1, rev(MNP.resp.ST1.C$Group.1)), y = c(MNP.lower.int.ST1.C, rev(MNP.upper.int.
ST1.C)), col = alpha("#31668EFF", alpha = 0.5), border = NA)
```

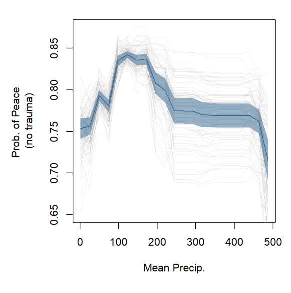

Supplemental Figure 62. Partial response of the probability of peace to variation in mean precipitation on the coast while all other predictor variables' effects are held constant.

Mean Temp

```
MNT.list.C <- list(pdp_partials.corr.pop.C$PDP.MNT.C.MNtemp20km)
MNT.resp.ST1.C <- aggregate(1-pdp_partials.corr.pop.C$PDP.MNT.C.yhat, by = MNT.list.C, FUN = mean)
MNT.resp.ST1.sdE.C <- aggregate(1-pdp_partials.corr.pop.C$PDP.MNT.C.yhat, by = MNT.list.C, FUN = sd)
MNT.margin.ST1.C <- qt(0.975, df = length(MNT.resp.ST1.C$Group.1)-1) * MNT.resp.ST1.sdE.C$x/sqrt(length(MNT.resp.
ST1.C$Group.1))#Calculate 95% CIs
MNT.lower.int.ST1.C <- MNT.resp.ST1.C$x - MNT.margin.ST1.C#get the lower 95% CI boundary
MNT.upper.int.ST1.C <- MNT.resp.ST1.C$x + MNT.margin.ST1.C#get the upper 95% CI boundary

from.seq.plot <- seq(1, 21000, 21)
to.seq.plot <- seq(21, 21000, 21)

par(pty = "s")
plot(MNT.resp.ST1.C$x~MNT.resp.ST1.C$Group.1, type = "l", ylab = "Prob. of Peace \n(no trauma)", xlab = "Mean Tem
p.", ylim = c(0.65, 0.875), col = "#482576FF")
for (i in 1:length(from.seq.plot)) {
  lines(1-pdp_partials.corr.pop.C$PDP.MNT.C.yhat[from.seq.plot[i]:to.seq.plot[i]]~pdp_partials.corr.pop.C$PDP.MN
T.C.MNtemp20km[1:21], col = alpha("grey50", alpha = 0.15), lwd = 0.5)
}
lines(MNT.resp.ST1.C$x~MNT.resp.ST1.C$Group.1, col = "#482576FF")
polygon(x = c(MNT.resp.ST1.C$Group.1, rev(MNT.resp.ST1.C$Group.1)), y = c(MNT.lower.int.ST1.C, rev(MNT.upper.int.
ST1.C)), col = alpha("#482576FF", alpha = 0.5), border = NA)
```

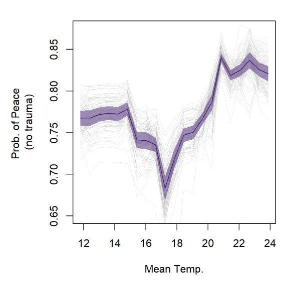

Supplemental Figure 63. Partial response of the probability of peace to variation in mean temperature on the coast while all other predictor variables' effects are held constant.

SST

```
SST.list.C <- list(pdp_partials.corr.pop.C$PDP.SST.C.SST)
SST.resp.ST1.C <- aggregate(1-pdp_partials.corr.pop.C$PDP.SST.C.yhat, by = SST.list.C, FUN = mean)
SST.resp.ST1.sdE.C <- aggregate(1-pdp_partials.corr.pop.C$PDP.SST.C.yhat, by = SST.list.C, FUN = sd)
SST.margin.ST1.C <- qt(0.975, df = length(SST.resp.ST1.C$Group.1)-1) * SST.resp.ST1.sdE.C$x/sqrt(length(SST.resp.
ST1.C$Group.1))#Calculate 95% CIs
SST.lower.int.ST1.C <- SST.resp.ST1.C$x - SST.margin.ST1.C#get the lower 95% CI boundary
SST.upper.int.ST1.C <- SST.resp.ST1.C$x + SST.margin.ST1.C#get the upper 95% CI boundary

from.seq.plot <- seq(1, 21000, 21)
to.seq.plot <- seq(21, 21000, 21)

par(pty = "s")
plot(SST.resp.ST1.C$x~SST.resp.ST1.C$Group.1, type = "l", ylab = "Prob. of Peace \n(no trauma)", xlab = "SST", yl
im = c(0.65, 0.875), col = "#1E9C89FF")
for (i in 1:length(from.seq.plot)) {
  lines(1-pdp_partials.corr.pop.C$PDP.SST.C.yhat[from.seq.plot[i]:to.seq.plot[i]]~pdp_partials.corr.pop.C$PDP.SS
T.C.SST[1:21], col = alpha("grey50", alpha = 0.15), lwd = 0.5)
}
lines(SST.resp.ST1.C$x~SST.resp.ST1.C$Group.1, col = "#1E9C89FF")
polygon(x = c(SST.resp.ST1.C$Group.1, rev(SST.resp.ST1.C$Group.1)), y = c(SST.lower.int.ST1.C, rev(SST.upper.int.
ST1.C)), col = alpha("#1E9C89FF", alpha = 0.5), border = NA)
```

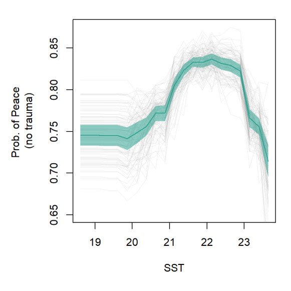

Supplemental Figure 64. Partial response of the probability of peace to variation in sea surface temperature on the coast while all other predictor variables' effects are held constant.

Population

```
POP.list.C <- list(pdp_partials.corr.pop.C$PDP.POP.C.corr.pop.KDE)
POP.resp.ST1.C <- aggregate(1-pdp_partials.corr.pop.C$PDP.POP.C.yhat, by = POP.list.C, FUN = mean)
POP.resp.ST1.sdE.C <- aggregate(1-pdp_partials.corr.pop.C$PDP.POP.C.yhat, by = POP.list.C, FUN = sd)
POP.margin.ST1.C <- qt(0.975, df = length(POP.resp.ST1.C$Group.1)-1) * POP.resp.ST1.sdE.C$x/sqrt(length(POP.resp.
ST1.C$Group.1))#Calculate 95% CIs
POP.lower.int.ST1.C <- POP.resp.ST1.C$x - POP.margin.ST1.C#get the lower 95% CI boundary
POP.upper.int.ST1.C <- POP.resp.ST1.C$x + POP.margin.ST1.C#get the upper 95% CI boundary

from.seq.plot <- seq(1, 21000, 21)
to.seq.plot <- seq(21, 21000, 21)

par(pty = "s")
plot(POP.resp.ST1.C$x~POP.resp.ST1.C$Group.1, type = "l", ylab = "Prob. of Peace \n(no trauma)", xlab = "corr. Po
pulation (KDE)", ylim = c(0.65, 0.875), col = "#FDE725FF")
for (i in 1:length(from.seq.plot)) {
  lines(1-pdp_partials.corr.pop.C$PDP.POP.C.yhat[from.seq.plot[i]:to.seq.plot[i]]~pdp_partials.corr.pop.C$PDP.PO
P.C.corr.pop.KDE[1:21], col = alpha("grey50", alpha =  0.15), lwd = 0.5)
}
lines(POP.resp.ST1.C$x~POP.resp.ST1.C$Group.1, col = "#FDE725FF")
polygon(x = c(POP.resp.ST1.C$Group.1, rev(POP.resp.ST1.C$Group.1)), y = c(POP.lower.int.ST1.C, rev(POP.upper.int.
ST1.C)), col = alpha("#FDE725FF", alpha = 0.5), border = NA)
```

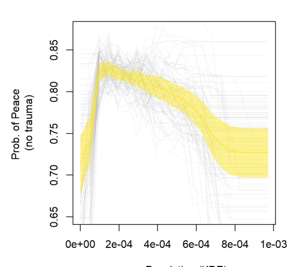

Supplemental Figure 65. Partial response of the probability of peace to variation in relative population size on the coast while all other predictor variables' effects are held constant.

ENSO

```
ENS.list.C <- list(pdp_partials.corr.pop.C$PDP.ENS.C.ENSO)
ENS.resp.ST1.C <- aggregate(1-pdp_partials.corr.pop.C$PDP.ENS.C.yhat, by = ENS.list.C, FUN = mean)
ENS.resp.ST1.sdE.C <- aggregate(1-pdp_partials.corr.pop.C$PDP.ENS.C.yhat, by = ENS.list.C, FUN = sd)
ENS.margin.ST1.C <- qt(0.975, df = length(ENS.resp.ST1.C$Group.1)-1) * ENS.resp.ST1.sdE.C$x/sqrt(length(ENS.resp.
ST1.C$Group.1))#Calculate 95% CIs
ENS.lower.int.ST1.C <- ENS.resp.ST1.C$x - ENS.margin.ST1.C#get the lower 95% CI boundary
ENS.upper.int.ST1.C <- ENS.resp.ST1.C$x + ENS.margin.ST1.C#get the upper 95% CI boundary

from.seq.plot <- seq(1, 21000, 21)
to.seq.plot <- seq(21, 21000, 21)

par(pty = "s")
plot(ENS.resp.ST1.C$x~ENS.resp.ST1.C$Group.1, type = "l", ylab = "Prob. of Peace \n(no trauma)", xlab = "ENSO Fre
q", ylim = c(0.65, 0.875), col = "#6BCD5AFF")
for (i in 1:length(from.seq.plot)) {
  lines(1-pdp_partials.corr.pop.C$PDP.ENS.C.yhat[from.seq.plot[i]:to.seq.plot[i]]~pdp_partials.corr.pop.C$PDP.EN
S.C.ENSO[1:21], col = alpha("grey50", alpha = 0.15), lwd = 0.5)
}
lines(ENS.resp.ST1.C$x~ENS.resp.ST1.C$Group.1, col = "#6BCD5AFF")
polygon(x = c(ENS.resp.ST1.C$Group.1, rev(ENS.resp.ST1.C$Group.1)), y = c(ENS.lower.int.ST1.C, rev(ENS.upper.int.
ST1.C)), col = alpha("#6BCD5AFF", alpha = 0.5), border = NA)
```

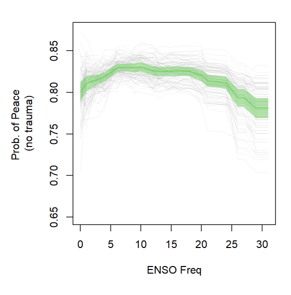

Supplemental Figure 66. Partial response of the probability of peace to variation in ENSO frequency on the coast while all other predictor variables' effects are held constant.

State

```
State.list.C <- list(pdp_partials.corr.pop.C$PDP.STT.C.Individuals_state)
State.resp.ST1.C <- aggregate(1-pdp_partials.corr.pop.C$PDP.STT.C.yhat, by = State.list.C, FUN = mean)
State.resp.ST1.sdE.C <- aggregate(1-pdp_partials.corr.pop.C$PDP.STT.C.yhat, by = State.list.C, FUN = sd)
State.margin.ST1.C <- qt(0.975, df = length(State.resp.ST1.C$Group.1)-1) * State.resp.ST1.sdE.C$x/sqrt(length(Sta
te.resp.ST1.C$Group.1))#Calculate 95% CIs
State.lower.int.ST1.C <- State.resp.ST1.C$x - State.margin.ST1.C#get the lower 95% CI boundary
State.upper.int.ST1.C <- State.resp.ST1.C$x + State.margin.ST1.C#get the upper 95% CI boundary

par(pty = "s")
plot(y = State.resp.ST1.C$x, x = as.numeric(State.resp.ST1.C$Group.1), pch = 19, col = c("black","black","blac
k"), ylab = "Prob. of Peace \n(no trauma)", xlab = "State Presence", ylim = c(0.65, 0.875), xaxt = "n")
for (i in 1:length(from.seq.plot)) {
  lines(1-pdp_partials.corr.pop.C$PDP.STT.C.yhat[from.seq.plot[i]:to.seq.plot[i]]~pdp_partials.corr.pop.C$PDP.ST
T.C.Individuals_state[1:21], col = alpha("grey50", alpha = 0.15), lwd = 0.5)
}
lines(State.resp.ST1.C$x~State.resp.ST1.C$Group.1, col = alpha("black", alpha = 0.5), lwd = 2)
points(y = State.resp.ST1.C$x, x = as.numeric(State.resp.ST1.C$Group.1), pch = 19, col = c("black","black","blac
k"), cex = 1.5)
axis(side = 1, at = c(1,2,3), labels = c(0,1,2), tick = TRUE)
```

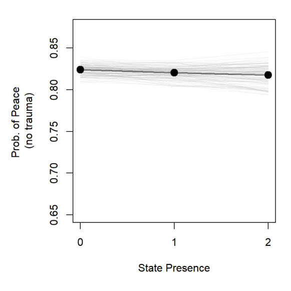

Supplemental Figure 67. Partial response of the probability of peace to variation in sociopolitical complexity on the coast while all other predictor variables' effects are held constant.

Now make a full plot for all 2D partial plots from the models made using taphonomically corrected KDE values for relative population estimation on the coasts.

```
from.seq.plot <- seq(1, 21000, 21)
to.seq.plot <- seq(21, 21000, 21)

par(mfrow = c(2,3))
par(pty = "s")
plot(MNT.resp.ST1.C$x~MNT.resp.ST1.C$Group.1, type = "l", ylab = "Prob. of Peace \n(no trauma)", xlab = "Mean Tem
p.", ylim = c(0.65, 0.875), col = "#482576FF")
for (i in 1:length(from.seq.plot)) {
  lines(1-pdp_partials.corr.pop.C$PDP.MNT.C.yhat[from.seq.plot[i]:to.seq.plot[i]]~pdp_partials.corr.pop.C$PDP.MN
T.C.MNtemp20km[1:21], col = alpha("grey50", alpha = 0.15), lwd = 0.5)
}

lines(MNT.resp.ST1.C$x~MNT.resp.ST1.C$Group.1, col = "#482576FF")
polygon(x = c(MNT.resp.ST1.C$Group.1, rev(MNT.resp.ST1.C$Group.1)), y = c(MNT.lower.int.ST1.C, rev(MNT.upper.int.
ST1.C)), col = alpha("#482576FF", alpha = 0.5), border = NA)

plot(MNP.resp.ST1.C$x~MNP.resp.ST1.C$Group.1, type = "l", ylab = "", yaxt = "n", xlab = "Mean Precip.", ylim = c(
0.65, 0.875), col = "#31668EFF")
for (i in 1:length(from.seq.plot)) {
  lines(1-pdp_partials.corr.pop.C$PDP.MNP.C.yhat[from.seq.plot[i]:to.seq.plot[i]]~pdp_partials.corr.pop.C$PDP.MN
P.C.MNprcp20km[1:21], col = alpha("grey50", alpha = 0.15), lwd = 0.5)
}

lines(MNP.resp.ST1.C$x~MNP.resp.ST1.C$Group.1, col = "#31668EFF")
```

```
polygon(x = c(MNP.resp.ST1.C$Group.1, rev(MNP.resp.ST1.C$Group.1)), y = c(MNP.lower.int.ST1.C, rev(MNP.upper.int.
ST1.C)), col = alpha("#31668EFF", alpha = 0.5), border = NA)

par(pty = "s")
plot(ENS.resp.ST1.C$x~ENS.resp.ST1.C$Group.1, type = "l", ylab = "", yaxt = "n", xlab = "ENSO Freq.", ylim = c(0.
65, 0.875), col = "#6BCD5AFF")
for (i in 1:length(from.seq.plot)) {
  lines(1-pdp_partials.corr.pop.C$PDP.ENS.C.yhat[from.seq.plot[i]:to.seq.plot[i]]~pdp_partials.corr.pop.C$PDP.EN
S.C.ENSO[1:21], col = alpha("grey50", alpha = 0.15), lwd = 0.5)
}

lines(ENS.resp.ST1.C$x~ENS.resp.ST1.C$Group.1, col = "#6BCD5AFF")
polygon(x = c(ENS.resp.ST1.C$Group.1, rev(ENS.resp.ST1.C$Group.1)), y = c(ENS.lower.int.ST1.C, rev(ENS.upper.int.
ST1.C)), col = alpha("#6BCD5AFF", alpha = 0.5), border = NA)

par(pty = "s")
plot(y = State.resp.ST1.C$x, x = as.numeric(State.resp.ST1.C$Group.1), pch = 19, col = "black", ylab = "Prob. of
Peace \n(no trauma)", xlab = "State Presence", ylim = c(0.65, 0.875), xaxt = "n")
for (i in 1:length(from.seq.plot)) {
  lines(1-pdp_partials.corr.pop.C$PDP.STT.C.yhat[from.seq.plot[i]:to.seq.plot[i]]~pdp_partials.corr.pop.C$PDP.ST
T.C.Individuals_state[1:21], col = alpha("grey50", alpha = 0.15), lwd = 0.5)
}
lines(State.resp.ST1.C$x~State.resp.ST1.C$Group.1, col = alpha("black", alpha = 0.5), lwd = 2)
points(y = State.resp.ST1.C$x, x = as.numeric(State.resp.ST1.C$Group.1), pch = 19, col = "black", cex = 1.5)
axis(side = 1, at = c(1,2,3), labels = c(0,1,2), tick = TRUE)

par(pty = "s")
plot(POP.resp.ST1.C$x~POP.resp.ST1.C$Group.1, type = "l", ylab = "", yaxt = "n", xlab = "corr. Population (KDE)",
ylim = c(0.65, 0.875), col = "#FDE725FF")
for (i in 1:length(from.seq.plot)) {
  lines(1-pdp_partials.corr.pop.C$PDP.POP.C.yhat[from.seq.plot[i]:to.seq.plot[i]]~pdp_partials.corr.pop.C$PDP.PO
P.C.corr.pop.KDE[1:21], col = alpha("grey50", alpha = 0.15), lwd = 0.5)
}

lines(POP.resp.ST1.C$x~POP.resp.ST1.C$Group.1, col = "#FDE725FF")
polygon(x = c(POP.resp.ST1.C$Group.1, rev(POP.resp.ST1.C$Group.1)), y = c(POP.lower.int.ST1.C, rev(POP.upper.int.
ST1.C)), col = alpha("#FDE725FF", alpha = 0.5), border = NA)

par(pty = "s")
plot(SST.resp.ST1.C$x~SST.resp.ST1.C$Group.1, type = "l", ylab = "", yaxt = "n", xlab = "SST", ylim = c(0.65, 0.8
75), col = "#1E9C89FF")
for (i in 1:length(from.seq.plot)) {
  lines(1-pdp_partials.corr.pop.C$PDP.SST.C.yhat[from.seq.plot[i]:to.seq.plot[i]]~pdp_partials.corr.pop.C$PDP.SS
T.C.SST[1:21], col = alpha("grey50", alpha = 0.15), lwd = 0.5)
}

lines(SST.resp.ST1.C$x~SST.resp.ST1.C$Group.1, col = "#1E9C89FF")
polygon(x = c(SST.resp.ST1.C$Group.1, rev(SST.resp.ST1.C$Group.1)), y = c(SST.lower.int.ST1.C, rev(SST.upper.int.
ST1.C)), col = alpha("#1E9C89FF", alpha = 0.5), border = NA)
```

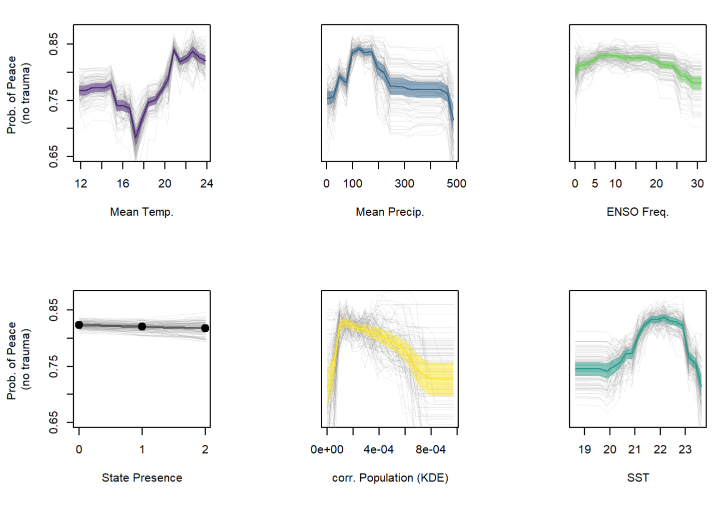

Supplemental Figure 68. Partial response plots for the probability of trauma to all predictor variables for coastal individuals. The partial response plots using the taphonomically corrected KDEs replicate the interpretive results of the models using the uncorrected KDEs precisely (compare with Supplemental Figure 30). This strongly suggests that use of taphonomically corrected or uncorrected population estimates has no impact on the model output. This result comes about because a) climate variables have higher variable importance, and therefore have more effect on model performance, and b) the majority of our data come from within the past 4000 years when taphonomic correction has less effect and the relative patterns in population over the past 4000 years are broadly consistent when using corrected or uncorrected population estimates from dates as data in the region. While on the coast prior to 4000 yBP with taphonomically corrected data produces a large population spike, after that point the relative patterns are quite consistent as with uncorrected patterns. As dates as data provides a relative estimate (we interpret simply as larger or smaller population rather than absolute population sizes) the consistency in relative pattern in the data (corrected or not) produces the same analytical pattern results.

Non-coastal Model Outcomes using taphonomically corrected KDEs.

Now we repeat all of the model evaluation for non-coastal individuals, beginning with variable importance.

```
MNT_Imp <- mean(all_performance.corr.pop.NC$MNT_Imp)#3rd
MNT_t <- t.test(all_performance.corr.pop.NC$MNT_Imp)

MNP_Imp <- mean(all_performance.corr.pop.NC$MNP_Imp)#1st
MNP_t <- t.test(all_performance.corr.pop.NC$MNP_Imp)

POP_Imp <- mean(all_performance.corr.pop.NC$POP_Imp)#5th
POP_t <- t.test(all_performance.corr.pop.NC$POP_Imp)

SDT_Imp <- mean(all_performance.corr.pop.NC$SDT_Imp)#6th
SDT_t <- t.test(all_performance.corr.pop.NC$SDT_Imp)

ENS_Imp <- mean(all_performance.corr.pop.NC$ENS_Imp)#2nd
ENS_t <- t.test(all_performance.corr.pop.NC$ENS_Imp)

STT_Imp <- mean(all_performance.corr.pop.NC$STT_Imp)#4th
STT_t <- t.test(all_performance.corr.pop.NC$STT_Imp)

#png(filename = "Inland.VarImp.png", height = 5, width = 5, units = "in", res = 300)
par(pty = "s", oma = c(2,2,0,0))
plot(x = MNP_Imp, y = 6, xlim = c(-0.001, 0.05), ylim = c(0,6), yaxt = "n", ylab = "", pch = 19, xlab = "", cex =
1.5, col = "#31668EFF")
lines(x = c(MNP_t$conf.int[1], MNP_t$conf.int[2]), y = c(6,6), col = "white")
points(x = SDT_Imp, y = 1, pch = 19, cex = 1.5,col = "#1E9C89FF")
lines(x = c(SDT_t$conf.int[1], SDT_t$conf.int[2]), y = c(1,1), col = "white")
points(x = MNT_Imp, y = 4, pch = 19, cex = 1.5,col = "#482576FF")
lines(x = c(MNT_t$conf.int[1], MNT_t$conf.int[2]), y = c(4,4), col = "white")
points(x = POP_Imp, y = 2, pch = 19, cex = 1.5,col = "#FDE725FF")
lines(x = c(POP_t$conf.int[1], POP_t$conf.int[2]), y = c(2,2), col = "white")
points(x = ENS_Imp, y = 5, pch = 19, cex = 1.5,col = "#6BCD5AFF")
lines(x = c(ENS_t$conf.int[1], ENS_t$conf.int[2]), y = c(5,5), col = "white")
points(x = STT_Imp, y = 3, pch = 19, cex = 1.5,col = "black")
lines(x = c(STT_t$conf.int[1], STT_t$conf.int[2]), y = c(3,3), col = "white")
axis(2, at = c(1,2,3,4,5,6), labels = FALSE, tick = TRUE)
mtext("Mean Precip.", at = 6, side = 2, line = 1, las = 1)
mtext("Mean Temp.", at = 4, side = 2, line = 1, las = 1)
mtext("Population (KDE)", at = 2, side = 2, line = 1, las = 1)
mtext("ENSO Freq.", at = 5, side = 2, line = 1, las = 1)
mtext("State", at = 3, side = 2, line = 1, las = 1)
mtext("St. Dev. Temp.", at = 1, side = 2, line = 1, las = 1)
mtext("Variable Importance", side = 1, line = 2)
```

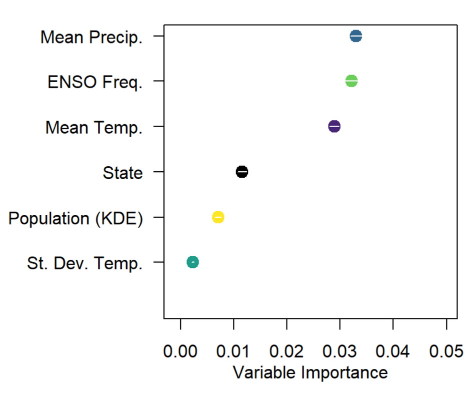


```
#dev.off()
```

Supplemental Figure 69. Variable importance for non-coastal models. Colored dots provide the central tendency value for variable importance from the aggregate 100 models while the white line provides the confidence interval. Like coastal outputs, the variable importance suggests that climate is more important than demographic and social variables in predicting the presence/absence of trauma. There is a slight change of order, with the standard deviation in temp reducing in variable importance while ENSO rose.

Mean Precip

```
MNP.list.NC <- list(pdp_partials.corr.pop.NC$PDP.MNP.NC.MNprcp20km)
MNP.resp.ST1.NC <- aggregate(1-pdp_partials.corr.pop.NC$PDP.MNP.NC.yhat, by = MNP.list.NC, FUN = mean)
MNP.resp.ST1.sdE.NC <- aggregate(1-pdp_partials.corr.pop.NC$PDP.MNP.NC.yhat, by = MNP.list.NC, FUN = sd)
MNP.margin.ST1.NC <- qt(0.975, df = length(MNP.resp.ST1.NC$Group.1)-1) * MNP.resp.ST1.sdE.NC$x/sqrt(length(MNP.re
sp.ST1.NC$Group.1))#Calculate 95% CIs
MNP.lower.int.ST1.NC <- MNP.resp.ST1.NC$x - MNP.margin.ST1.NC#get the lower 95% CI boundary
MNP.upper.int.ST1.NC <- MNP.resp.ST1.NC$x + MNP.margin.ST1.NC#get the upper 95% CI boundary

from.seq.plot <- seq(1, 21000, 21)
to.seq.plot <- seq(21, 21000, 21)

par(pty = "s")
plot(MNP.resp.ST1.NC$x~MNP.resp.ST1.NC$Group.1, type = "l", ylab = "Prob. of Peace \n(no trauma)", xlab = "Mean P
recip.", ylim = c(0.6, 0.825), col = "#31668EFF")
for (i in 1:length(from.seq.plot)) {
  lines(1-pdp_partials.corr.pop.NC$PDP.MNP.NC.yhat[from.seq.plot[i]:to.seq.plot[i]]~pdp_partials.corr.pop.NC$PDP.
MNP.NC.MNprcp20km[1:21], col = alpha("grey50", alpha = 0.15), lwd = 0.5)
}
lines(MNP.resp.ST1.NC$x~MNP.resp.ST1.NC$Group.1, col = "#31668EFF")
polygon(x = c(MNP.resp.ST1.NC$Group.1, rev(MNP.resp.ST1.NC$Group.1)), y = c(MNP.lower.int.ST1.NC, rev(MNP.upper.i
nt.ST1.NC)), col = alpha("#31668EFF", alpha = 0.5), border = NA)
```

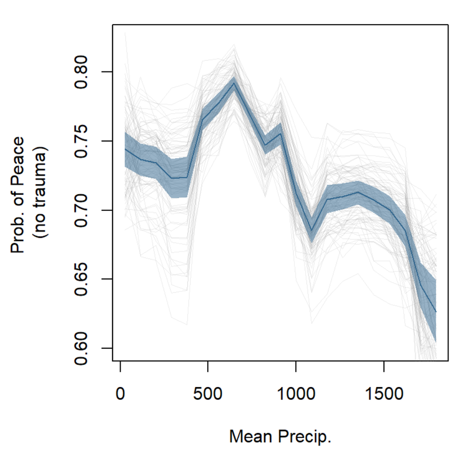

Supplemental Figure 70. Partial response of the probability of peace to variation in mean precipitation for non-coastal individuals while all other predictor variables' effects are held constant.

Partial for Mean Temp

```
MNT.list.NC <- list(pdp_partials.corr.pop.NC$PDP.MNT.NC.MNtemp20km)
MNT.resp.ST1.NC <- aggregate(1-pdp_partials.corr.pop.NC$PDP.MNT.NC.yhat, by = MNT.list.NC, FUN = mean)
MNT.resp.ST1.sdE.NC <- aggregate(1-pdp_partials.corr.pop.NC$PDP.MNT.NC.yhat, by = MNT.list.NC, FUN = sd)
MNT.margin.ST1.NC <- qt(0.975, df = length(MNT.resp.ST1.NC$Group.1)-1) * MNT.resp.ST1.sdE.NC$x/sqrt(length(MNT.re
sp.ST1.NC$Group.1))#Calculate 95% CIs
MNT.lower.int.ST1.NC <- MNT.resp.ST1.NC$x - MNT.margin.ST1.NC#get the lower 95% CI boundary
MNT.upper.int.ST1.NC <- MNT.resp.ST1.NC$x + MNT.margin.ST1.NC#get the upper 95% CI boundary

from.seq.plot <- seq(1, 21000, 21)
to.seq.plot <- seq(21, 21000, 21)

par(pty = "s")
plot(MNT.resp.ST1.NC$x~MNT.resp.ST1.NC$Group.1, type = "l", ylab = "Prob. of Peace \n(no trauma)", xlab = "Mean T
emp.", ylim = c(0.6, 0.825), col = "#482576FF")
for (i in 1:length(from.seq.plot)) {
  lines(1-pdp_partials.corr.pop.NC$PDP.MNT.NC.yhat[from.seq.plot[i]:to.seq.plot[i]]~pdp_partials.corr.pop.NC$PDP.
MNT.NC.MNtemp20km[1:21], col = alpha("grey50", alpha = 0.15), lwd = 0.5)
}
lines(MNT.resp.ST1.NC$x~MNT.resp.ST1.NC$Group.1, col = "#482576FF")
polygon(x = c(MNT.resp.ST1.NC$Group.1, rev(MNT.resp.ST1.NC$Group.1)), y = c(MNT.lower.int.ST1.NC, rev(MNT.upper.i
nt.ST1.NC)), col = alpha("#482576FF", alpha = 0.5), border = NA)
```

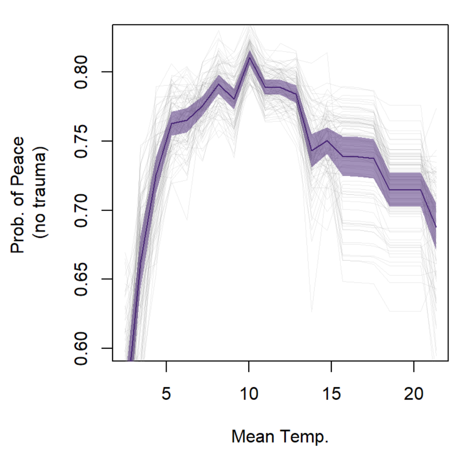

Supplemental Figure 71. Partial response of the probability of peace to variation in mean temperature for non-coastal individuals while all other predictor variables' effects are held constant.

St. Dev. Temp

```
SDT.list.NC <- list(pdp_partials.corr.pop.NC$PDP.SDT.NC.SDtemp20km)
SDT.resp.ST1.NC <- aggregate(1-pdp_partials.corr.pop.NC$PDP.SDT.NC.yhat, by = SDT.list.NC, FUN = mean)
SDT.resp.ST1.sdE.NC <- aggregate(1-pdp_partials.corr.pop.NC$PDP.SDT.NC.yhat, by = SDT.list.NC, FUN = sd)
SDT.margin.ST1.NC <- qt(0.975, df = length(SDT.resp.ST1.NC$Group.1)-1) * SDT.resp.ST1.sdE.NC$x/sqrt(length(SDT.re
sp.ST1.NC$Group.1))#Calculate 95% CIs
SDT.lower.int.ST1.NC <- SDT.resp.ST1.NC$x - SDT.margin.ST1.NC#get the lower 95% CI boundary
SDT.upper.int.ST1.NC <- SDT.resp.ST1.NC$x + SDT.margin.ST1.NC#get the upper 95% CI boundary

from.seq.plot <- seq(1, 21000, 21)
to.seq.plot <- seq(21, 21000, 21)

par(pty = "s")
plot(SDT.resp.ST1.NC$x~SDT.resp.ST1.NC$Group.1, type = "l", ylab = "Prob. of Peace \n(no trauma)", xlab = "SDT",
ylim = c(0.6, 0.825), col = "#1E9C89FF")
for (i in 1:length(from.seq.plot)) {
  lines(1-pdp_partials.corr.pop.NC$PDP.SDT.NC.yhat[from.seq.plot[i]:to.seq.plot[i]]~pdp_partials.corr.pop.NC$PDP.
SDT.NC.SDtemp20km[1:21], col = alpha("grey50", alpha = 0.15), lwd = 0.5)
}
lines(SDT.resp.ST1.NC$x~SDT.resp.ST1.NC$Group.1, col = "#1E9C89FF")
polygon(x = c(SDT.resp.ST1.NC$Group.1, rev(SDT.resp.ST1.NC$Group.1)), y = c(SDT.lower.int.ST1.NC, rev(SDT.upper.i
nt.ST1.NC)), col = alpha("#1E9C89FF", alpha = 0.5), border = NA)
```

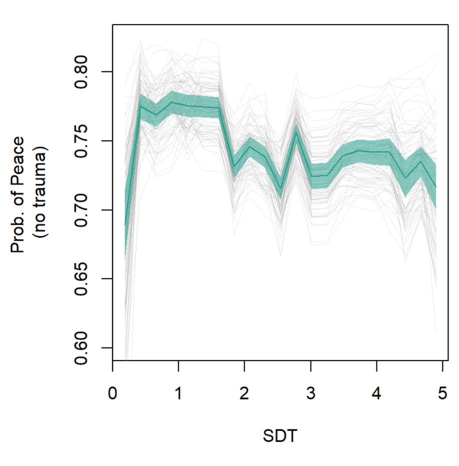

Supplemental Figure 72. Partial response of the probability of peace to variation in standard deviation in temperature for non-coastal individuals while all other predictor variables' effects are held constant.

Population Density

```
POP.list.NC <- list(pdp_partials.corr.pop.NC$PDP.POP.NC.corr.pop.KDE)
POP.resp.ST1.NC <- aggregate(1-pdp_partials.corr.pop.NC$PDP.POP.NC.yhat, by = POP.list.NC, FUN = mean)
POP.resp.ST1.sdE.NC <- aggregate(1-pdp_partials.corr.pop.NC$PDP.POP.NC.yhat, by = POP.list.NC, FUN = sd)
POP.margin.ST1.NC <- qt(0.975, df = length(POP.resp.ST1.NC$Group.1)-1) * POP.resp.ST1.sdE.NC$x/sqrt(length(POP.re
sp.ST1.NC$Group.1))#Calculate 95% CIs
POP.lower.int.ST1.NC <- POP.resp.ST1.NC$x - POP.margin.ST1.NC#get the lower 95% CI boundary
POP.upper.int.ST1.NC <- POP.resp.ST1.NC$x + POP.margin.ST1.NC#get the upper 95% CI boundary

from.seq.plot <- seq(1, 21000, 21)
to.seq.plot <- seq(21, 21000, 21)

par(pty = "s")
plot(POP.resp.ST1.NC$x~POP.resp.ST1.NC$Group.1, type = "l", ylab = "Prob. of Peace \n(no trauma)", xlab = "Popula
tion Size", ylim = c(0.6, 0.82525), col = "#FDE725FF")
for (i in 1:length(from.seq.plot)) {
  lines(1-pdp_partials.corr.pop.NC$PDP.POP.NC.yhat[from.seq.plot[i]:to.seq.plot[i]]~pdp_partials.corr.pop.NC$PDP.
POP.NC.corr.pop.KDE[1:21], col = alpha("grey50", alpha =  0.15), lwd = 0.5)
}
lines(POP.resp.ST1.NC$x~POP.resp.ST1.NC$Group.1, col = "#FDE725FF")
polygon(x = c(POP.resp.ST1.NC$Group.1, rev(POP.resp.ST1.NC$Group.1)), y = c(POP.lower.int.ST1.NC, rev(POP.upper.i
nt.ST1.NC)), col = alpha("#FDE725FF", alpha = 0.5), border = NA)
```

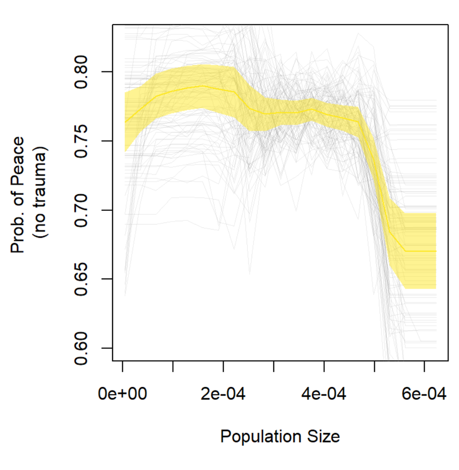

Supplemental Figure 73. Partial response of the probability of peace to variation in relative population size for non-coastal individuals while all other predictor variables' effects are held constant.

ENSO

```
ENS.list.NC <- list(pdp_partials.corr.pop.NC$PDP.ENS.NC.ENSO)
ENS.resp.ST1.NC <- aggregate(1-pdp_partials.corr.pop.NC$PDP.ENS.NC.yhat, by = ENS.list.NC, FUN = mean)
ENS.resp.ST1.sdE.NC <- aggregate(1-pdp_partials.corr.pop.NC$PDP.ENS.NC.yhat, by = ENS.list.NC, FUN = sd)
ENS.margin.ST1.NC <- qt(0.975, df = length(ENS.resp.ST1.NC$Group.1)-1) * ENS.resp.ST1.sdE.NC$x/sqrt(length(ENS.re
sp.ST1.NC$Group.1))#Calculate 95% CIs
ENS.lower.int.ST1.NC <- ENS.resp.ST1.NC$x - ENS.margin.ST1.NC#get the lower 95% CI boundary
ENS.upper.int.ST1.NC <- ENS.resp.ST1.NC$x + ENS.margin.ST1.NC#get the upper 95% CI boundary

from.seq.plot <- seq(1, 21000, 21)
to.seq.plot <- seq(21, 21000, 21)

par(pty = "s")
plot(ENS.resp.ST1.NC$x~ENS.resp.ST1.NC$Group.1, type = "l", ylab = "Prob. of Peace \n(no trauma)", xlab = "ENSO F
req", ylim = c(0.6, 0.825), col = "#6BCD5AFF")
for (i in 1:length(from.seq.plot)) {
  lines(1-pdp_partials.corr.pop.NC$PDP.ENS.NC.yhat[from.seq.plot[i]:to.seq.plot[i]]~pdp_partials.corr.pop.NC$PDP.
ENS.NC.ENSO[1:21], col = alpha("grey50", alpha = 0.15), lwd = 0.5)
}
lines(ENS.resp.ST1.NC$x~ENS.resp.ST1.NC$Group.1, col = "#6BCD5AFF")
polygon(x = c(ENS.resp.ST1.NC$Group.1, rev(ENS.resp.ST1.NC$Group.1)), y = c(ENS.lower.int.ST1.NC, rev(ENS.upper.i
nt.ST1.NC)), col = alpha("#6BCD5AFF", alpha = 0.5), border = NA)
```

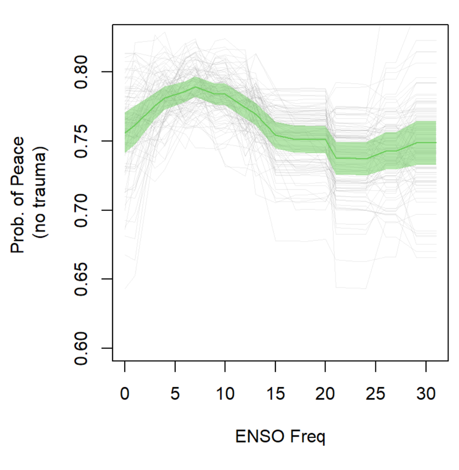

Supplemental Figure 74. Partial response of the probability of peace to variation in ENSO frequency per century for non-coastal individuals while all other predictor variables' effects are held constant.

State

```
State.list.NC <- list(pdp_partials.corr.pop.NC$PDP.STT.NC.Individuals_state)
State.resp.ST1.NC <- aggregate(1-pdp_partials.corr.pop.NC$PDP.STT.NC.yhat, by = State.list.NC, FUN = mean)
State.resp.ST1.sdE.NC <- aggregate(1-pdp_partials.corr.pop.NC$PDP.STT.NC.yhat, by = State.list.NC, FUN = sd)
State.margin.ST1.NC <- qt(0.975, df = length(State.resp.ST1.NC$Group.1)-1) * State.resp.ST1.sdE.NC$x/sqrt(length(
State.resp.ST1.NC$Group.1))#Calculate 95% CIs
State.lower.int.ST1.NC <- State.resp.ST1.NC$x - State.margin.ST1.NC#get the lower 95% CI boundary
State.upper.int.ST1.NC <- State.resp.ST1.NC$x + State.margin.ST1.NC#get the upper 95% CI boundary

par(pty = "s")
plot(y = State.resp.ST1.NC$x, x = as.numeric(State.resp.ST1.NC$Group.1), pch = 19, col = c("black","black","blac
k"), ylab = "Prob. of Peace \n(no trauma)", xlab = "State Presence", ylim = c(0.6, 0.825), xaxt = "n")
for (i in 1:length(from.seq.plot)) {
  lines(1-pdp_partials.corr.pop.NC$PDP.STT.NC.yhat[from.seq.plot[i]:to.seq.plot[i]]~pdp_partials.corr.pop.NC$PDP.
STT.NC.Individuals_state[1:21], col = alpha("grey50", alpha = 0.15), lwd = 0.5)
}
lines(State.resp.ST1.NC$x~State.resp.ST1.NC$Group.1, col = alpha("black", alpha = 0.5), lwd = 2)
points(y = State.resp.ST1.NC$x, x = as.numeric(State.resp.ST1.NC$Group.1), pch = 19, col = c("black","black","bla
ck"), cex = 1.5)
axis(side = 1, at = c(1,2,3), labels = c(0,1,2), tick = TRUE)
```

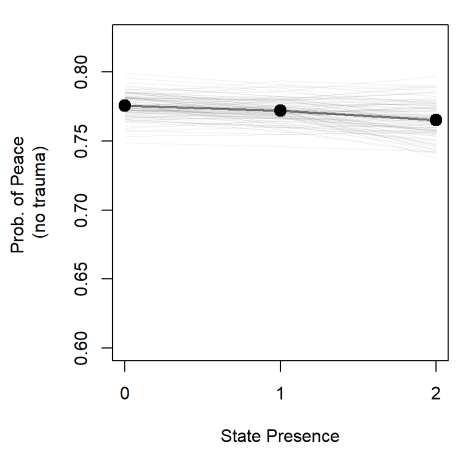

Supplemental Figure 75. Partial response of the probability of peace to variation in sociopolitical complexity for non-coastal individuals while all other predictor variables' effects are held constant.

Now we make a full plot of all 2D partials from the 100 RF models for non-coastal individuals using the taphonomically corrected versions of KDEs for relative population estimation.

```
from.seq.plot <- seq(1, 21000, 21)
to.seq.plot <- seq(21, 21000, 21)

par(mfrow = c(2,3))
#7AD151FF
par(pty = "s")
plot(MNP.resp.ST1.NC$x~MNP.resp.ST1.NC$Group.1, type = "l", ylab = "Prob. of Peace \n(no trauma)", xlab = "Mean P
recip.", ylim = c(0.6, 0.825), col = "#31668EFF")
for (i in 1:length(from.seq.plot)) {
  lines(1-pdp_partials.corr.pop.NC$PDP.MNP.NC.yhat[from.seq.plot[i]:to.seq.plot[i]]~pdp_partials.corr.pop.NC$PDP.
MNP.NC.MNprcp20km[1:21], col = alpha("grey50", alpha = 0.15), lwd = 0.5)
}

lines(MNP.resp.ST1.NC$x~MNP.resp.ST1.NC$Group.1, col = "#31668EFF")
polygon(x = c(MNP.resp.ST1.NC$Group.1, rev(MNP.resp.ST1.NC$Group.1)), y = c(MNP.lower.int.ST1.NC, rev(MNP.upper.i
nt.ST1.NC)), col = alpha("#31668EFF", alpha = 0.5), border = NA)

par(pty = "s")
plot(SDT.resp.ST1.NC$x~SDT.resp.ST1.NC$Group.1, type = "l", ylab = "", yaxt = "n", xlab = "SD. Dev. Temp", ylim =
c(0.6, 0.825), col = "#1E9C89FF")
for (i in 1:length(from.seq.plot)) {
  lines(1-pdp_partials.corr.pop.NC$PDP.SDT.NC.yhat[from.seq.plot[i]:to.seq.plot[i]]~pdp_partials.corr.pop.NC$PDP.
SDT.NC.SDtemp20km[1:21], col = alpha("grey50", alpha = 0.15), lwd = 0.5)
}

lines(SDT.resp.ST1.NC$x~SDT.resp.ST1.NC$Group.1, col = "#1E9C89FF")
polygon(x = c(SDT.resp.ST1.NC$Group.1, rev(SDT.resp.ST1.NC$Group.1)), y = c(SDT.lower.int.ST1.NC, rev(SDT.upper.i
nt.ST1.NC)), col = alpha("#1E9C89FF", alpha = 0.5), border = NA)

par(pty = "s")
plot(MNT.resp.ST1.NC$x~MNT.resp.ST1.NC$Group.1, type = "l", ylab = "", yaxt = "n", xlab = "Mean Temp.", ylim = c(
0.6, 0.825), col = "#482576FF")
for (i in 1:length(from.seq.plot)) {
  lines(1-pdp_partials.corr.pop.NC$PDP.MNT.NC.yhat[from.seq.plot[i]:to.seq.plot[i]]~pdp_partials.corr.pop.NC$PDP.
MNT.NC.MNtemp20km[1:21], col = alpha("grey50", alpha = 0.15), lwd = 0.5)
}

lines(MNT.resp.ST1.NC$x~MNT.resp.ST1.NC$Group.1, col = "#482576FF")
polygon(x = c(MNT.resp.ST1.NC$Group.1, rev(MNT.resp.ST1.NC$Group.1)), y = c(MNT.lower.int.ST1.NC, rev(MNT.upper.i
nt.ST1.NC)), col = alpha("#482576FF", alpha = 0.5), border = NA)

par(pty = "s")
plot(POP.resp.ST1.NC$x~POP.resp.ST1.NC$Group.1, type = "l", ylab = "Prob. of Peace \n(no trauma)", xlab = "Popula
tion (KDE)", ylim = c(0.6, 0.825), col = "#FDE725FF")
for (i in 1:length(from.seq.plot)) {
  lines(1-pdp_partials.corr.pop.NC$PDP.POP.NC.yhat[from.seq.plot[i]:to.seq.plot[i]]~pdp_partials.corr.pop.NC$PDP.
POP.NC.corr.pop.KDE[1:21], col = alpha("grey50", alpha = 0.15), lwd = 0.5)
}

lines(POP.resp.ST1.NC$x~POP.resp.ST1.NC$Group.1, col = "#FDE725FF")
polygon(x = c(POP.resp.ST1.NC$Group.1, rev(POP.resp.ST1.NC$Group.1)), y = c(POP.lower.int.ST1.NC, rev(POP.upper.i
nt.ST1.NC)), col = alpha("#FDE725FF", alpha = 0.5), border = NA)

par(pty = "s")
plot(ENS.resp.ST1.NC$x~ENS.resp.ST1.NC$Group.1, type = "l", ylab = "", yaxt = "n", xlab = "ENSO Freq.", ylim = c(
0.6, 0.825), col = "#6BCD5AFF")
for (i in 1:length(from.seq.plot)) {
  lines(1-pdp_partials.corr.pop.NC$PDP.ENS.NC.yhat[from.seq.plot[i]:to.seq.plot[i]]~pdp_partials.corr.pop.NC$PDP.
ENS.NC.ENSO[1:21], col = alpha("grey50", alpha = 0.15), lwd = 0.5)
}

lines(ENS.resp.ST1.NC$x~ENS.resp.ST1.NC$Group.1, col = "#6BCD5AFF")
polygon(x = c(ENS.resp.ST1.NC$Group.1, rev(ENS.resp.ST1.NC$Group.1)), y = c(ENS.lower.int.ST1.NC, rev(ENS.upper.i
nt.ST1.NC)), col = alpha("#6BCD5AFF", alpha = 0.5), border = NA)

par(pty = "s") #c("#F7C5A5FF","#CB1B4FFF","#261433FF")
plot(y = State.resp.ST1.NC$x, x = as.numeric(State.resp.ST1.NC$Group.1), pch = 19, col = "black", ylab = "", yaxt
= "n", xlab = "State Presence", ylim = c(0.6, 0.825), xaxt = "n")
for (i in 1:length(from.seq.plot)) {
  lines(1-pdp_partials.corr.pop.NC$PDP.STT.NC.yhat[from.seq.plot[i]:to.seq.plot[i]]~pdp_partials.corr.pop.NC$PDP.
STT.NC.Individuals_state[1:21], col = alpha("grey50", alpha = 0.15), lwd = 0.5)
}
lines(State.resp.ST1.NC$x~State.resp.ST1.NC$Group.1, col = alpha("black", alpha = 0.5), lwd = 2)
points(y = State.resp.ST1.NC$x, x = as.numeric(State.resp.ST1.NC$Group.1), pch = 19, col = "black", cex = 1.5)
axis(side = 1, at = c(1,2,3), labels = c(0,1,2), tick = TRUE)
```

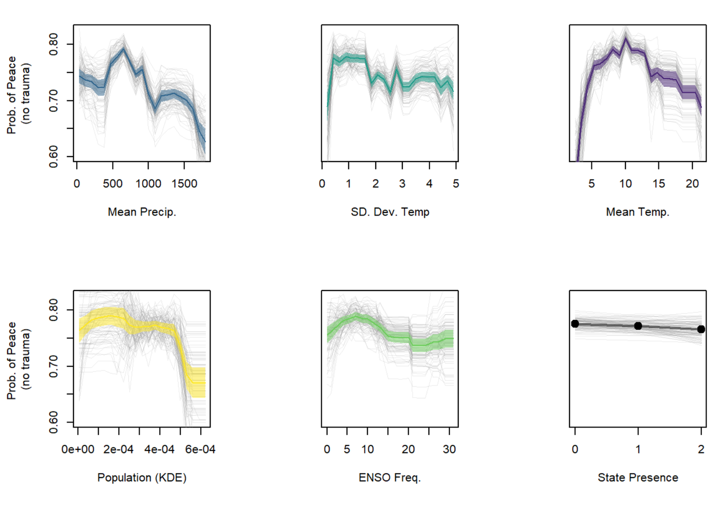

Supplemental Figure 76. Partial response plots for the probability of trauma to all predictor variables for non-coastal individuals. Like the coastal results, these replicate the results of the model made using the uncorrected KDEs. The method of processing the radiocarbon data for relative past population size estimation does not impact the interpretive results of the analyses.
